# Acupuncture therapy for post-stroke spasticity: a systematic review and exploratory network meta-analysis of clinical efficacy and dose–response relationship

**DOI:** 10.3389/fneur.2026.1782831

**Published:** 2026-05-25

**Authors:** Zhihao Xiong, Juwei Dong, Yini Hua, Yingying Zhu, Yue Song, Ziniu Zhang, Fengjia Xiao, Jinxia Ni, Liangxiao Ma, Jing Bai

**Affiliations:** 1 Dongzhimen Hospital, Beijing University of Chinese Medicine, Beijing, China; 2 School of Acupuncture-Moxibustion and Tuina, Beijing University of Chinese Medicine, Beijing, China; 3 The Key Unit of State Administration of Traditional Chines Medicine, Evaluation of Characteristic Acupuncture Therapy, Beijing, China

**Keywords:** acupuncture, efficacy, network meta-analysis, post-stroke spasticity, systematic review

## Abstract

**Background and purpose:**

Acupuncture has shown good therapeutic potential in post-stroke spasticity (PSS). We aimed to evaluate the clinical efficacy and optimal acupuncture type and dose of acupuncture for PSS.

**Methods:**

A comprehensive literature search was conducted across 8 databases (PubMed, Embase, Web of Science, Cochrane Library, Chinese National Knowledge Infrastructure (CNKI), Chinese Science and Technology Periodical Databases (VIP), Chinese Biomedical Literature database (Sinomed), and Wan Fang database) to search randomized controlled trials (RCTs) of acupuncture for PSS from their inception to December 1, 2025. Data analysis and network meta-analysis (NMA) were conducted using StataMP 18 software. The risk of bias was evaluated using RevMan 5.3 software. Adverse events (AEs) related to acupuncture were collected to assess the safety of acupuncture therapy. The optimal type and dose of acupuncture for anti-spasticity effects were evaluated by using the acupuncture dose-effect relationship evaluation model. The Grading of Recommendations, Assessment, Development, and Evaluation (GRADE) were employed to assess evidence reliability and certainty.

**Results:**

This meta-analysis included 34 trials and 3,383 PSS patients, with manual acupuncture (MA), warm acupuncture (WA), scalp acupuncture (SA), fire acupuncture (FA), and electro-acupuncture (EA). The findings showed that acupuncture significantly improved the Fugl-Meyer Assessment (FMA) [FMA-U: MD = 0.87, 95% CI (0.68, 1.07), *p* < 0.001, *I*^2^ = 84.97%; FMA-L: MD = 1.00, 95% CI (0.73, 1.27), *p* < 0.001, *I*^2^ = 87.57%], Modified Ashworth Scale (MAS) and Clinic Spasticity Index (CSI) [SMD = −1.03, 95% CI (−1.32, −0.75), *p* < 0.001, *I*^2^ = 87.53%], and Modified Barthel Index (MBI) [MD = 0.89, 95% CI (0.68, 1.10), *p* < 0.001, *I*^2^ = 81.78%] scores in PSS patients. AEs included mild pain, nausea, and dizziness. The NMA results indicated that WA [SMD = −1.66, 95% CI (−2.42 to −0.90), SUCRA = 94.6%] and high-dose [SMD = −1.11, 95% CI (−1.55 to −0.68), SUCRA = 78.5%] were the best intervention for improving spasticity scores.

**Conclusion:**

Acupuncture can improve the spasticity, motor function and activities of daily living of stroke patients; WA and high-dose showed the highest probability of being optimal intervention for spasticity; however, the certainty of evidence remains limited.

**Systematic review registration:**

https://www.crd.york.ac.uk/PROSPERO/view/CRD42025633455, identifier (CRD42025633455).

## Graphical abstract

**Figure:**
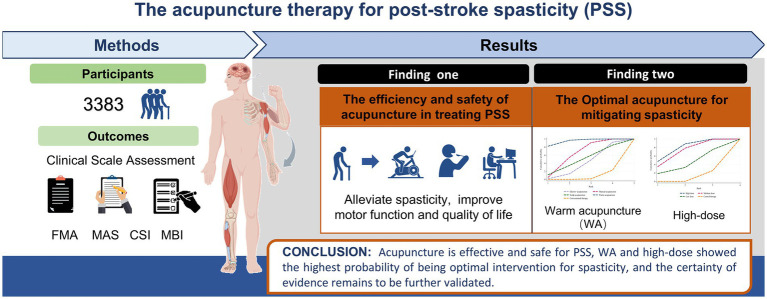

## 1 Introduction

Post-stroke spasticity (PSS), characterized by limb muscle stiffness, joint contracture, movement limitation, elevated muscle tone, and hyperactive tendon reflexes, is a hallmark manifestation of upper motor neuron syndrome (1) and severely impairs the quality of life of stroke survivors. With the acceleration of population aging and the growing prevalence of stroke, the incidence of PSS has risen steadily. It is estimated that approximately 25.3 to 39.5% of stroke survivors develop limb spasticity within 3 months post-onset (2), and the severity of spasticity tends to worsen over time (3). Thus, PSS is recognized as one of the most debilitating symptoms during stroke rehabilitation (4). Empirical evidence suggests that antispastic medications—including baclofen, tizanidine, diazepam, and dantrolene—play a crucial role in spasticity management (5). However, these agents may induce adverse effects such as fatigue, somnolence, muscle weakness, and even liver injury, which consequently reduce patient adherence to treatment.

Acupuncture serves as an effective complementary and alternative therapy for PSS, with its positive therapeutic effects having gained widespread recognition across 11 countries (6, 7). Recent research demonstrates that acupuncture can alleviate spasticity in PSS patients, enhance their motor function and quality of life, and has been designated as a Grade B recommended intervention for post-stroke spasticity in relevant clinical guidelines (8). Relevant animal experiments suggest that acupuncture may exert antispastic effects by regulating the GABAergic system and activating the Wnt/β-catenin signaling pathway (9). In clinical practice, the BAN2022 Guideline recommends acupuncture for improving post-stroke spasticity (10), while the Chinese Guidelines for Rehabilitation Management of Cerebrovascular Diseases (2nd Edition) explicitly states that acupuncture is beneficial for relieving spasticity (Class IIa recommendation, Grade B evidence) (11). However, previous studies have predominantly focused on verifying the efficacy of acupuncture for PSS and optimizing combinations of antispastic acupoints (12, 13). To date, there is still no systematic review that comprehensively evaluated the types and doses of acupuncture for antispasticity. Similarly, existing randomized controlled trials (RCTs) are constrained by inconsistent assessment criteria or small sample sizes, indicating that the effectiveness of acupuncture for PSS requires further validation (14). Therefore, we conducted this network meta-analysis.

In this study, we aimed to further evaluate the efficacy and safety of acupuncture in treating PSS, and to explore the effects of different acupuncture types and doses on alleviating spasticity. It also aimed to explore the optimal acupuncture method and dosage for anti-spasticity, providing evidence for the construction and optimization of clinical treatment plans.

## 2 Methods

The protocol of this meta-analysis was registered with the International Platform for the Registration of Systematic Review (PROSPERO) under registration number CRD42025633455. This study was conducted in strict adherence to the Preferred Reporting Items for Systematic Reviews and Meta-Analyses (PRISMA) guidelines (15, 16).

### 2.1 Search strategy

We systematically searched both Chinese and English databases, including Embase, PubMed, Cochrane Library, Web of Science, Chinese National Knowledge Infrastructure (CNKI), Chinese Science and Technology Periodical Databases (VIP), Chinese Biomedical Literature Database (Sinomed), and Wan Fang database, from the inception of each database to December 1, 2025. The search strategy comprises four components, with language restricted to English or Chinese. The components are as follows: (1) post-stroke spasticity (PSS or spastic hemiplegia); (2) acupuncture (including manual acupuncture, warm acupuncture, fire acupuncture, etc.); (3) clinical trials (e.g., randomized controlled trials, therapeutic effect observation, clinical research); (4) patients with stroke. The search strategy for each database is provided in Supplementary file 1.

### 2.2 Data extraction

All retrieved literatures were imported into NoteExpress. After deduplication, two independent reviewers (JD, FX) screened titles and abstracts for eligibility assessment; discrepancies were resolved by the third investigator (JX-N). Subsequently, reviewers conducted a comprehensive evaluation of full texts against the inclusion and exclusion criteria to confirm final eligibility. Finally, we extracted data from included literatures using Excel, covering the following general characteristics: author, country, publication year, patient age, stroke type, disease duration, intervention, outcome measures, and adverse events, among others.

### 2.3 Selection criteria

Inclusion criteria: (1) Participants: stroke patients (aged 35–85 years) diagnosed by CT or MRI, with increased muscle tone and hyperreflexia. (2) Interventions: various acupuncture therapies (manual acupuncture, warm acupuncture, scalp acupuncture, fire acupuncture, and electro-acupuncture), either alone or in combination with rehabilitation or conventional treatment. (3) Comparators: rehabilitation, conventional treatment, or sham acupuncture. (4) Outcomes: Fugl-Meyer Assessment (FMA), Modified Ashworth Scale (MAS), Modified Barthel Index (MBI), Clinical Spasticity Index (CSI), and Adverse Events (AEs). (5) Study design: Randomized controlled trials (RCTs). (6) Languages: Chinese and English.

Exclusion criteria: (1) Spasticity caused by non-stroke disease, such as spinal cord injury and traumatic brain injury. (2) Non-RCT, including animal experiments, case reports, reviews, and conference papers etc. (3) Total sample size <25. (4) Lack of valid outcome or data.

### 2.4 Data synthesis

This study assessed the effects of acupuncture on patients’ spasticity, motor function, and activities of daily living (ADL) by comparing baseline-to-post-intervention changes. Data processing followed the steps below: (1) Data extraction: For each trial, the mean and standard deviation (SD) of continuous outcomes (FMA, MAS, CSI, MBI) were extracted separately for the intervention and control groups at both baseline and post-intervention. If a study reported results at multiple follow-up time points, only data from the final time point were included in the analysis. (2) Data transformation: When SD was directly reported, the provided values were retained. Where SD was not available but standard error of the mean (SEM) or 95% confidence interval (95% CI) was provided, SD was imputed by using Equation 1 from the *Cochrane Handbook for Systematic Reviews of Interventions*. We estimated the standard deviation of the baseline-to-post-intervention change (SD_E,change_) by using the Equation 2 (17), where the correlation coefficient *R*-value was 0.5 (18).

$$\mathrm{SD}=(\text{Upper limit}-\text{lower limit})\times \sqrt{n}/3.92$$ (1)

$${\mathrm{SD}}_{E,\text{change}}=\sqrt{\begin{array}{l} {\mathrm{SD}}_{E,\text{baseline}}^{2}+{\mathrm{SD}}_{E,\text{final}}^{2}-\\ \left(2\times R\times {\mathrm{SD}}_{E,\text{baseline}}\times {\mathrm{SD}}_{E,\text{final}}\right) \end{array}}$$ (2)

### 2.5 Acupuncture dose-effect relationship evaluation

The Acupuncture Dose–Response Relationship Scoring System (16, 19) consists four core parameters: (1) number of acupuncture points; (2) “deqi” response; (3) weekly treatment frequency; (4) total treatment sessions. One trial is categorized as high dose if it satisfies any of the following criteria: (1) Above 9 acupuncture points; (2) Reporting “deqi” response; (3) Above 2 treatments per week; (4) Above 8 total treatment sessions. If a parameter is below these levels, it is defined as low dose (16). Based on the sum of the scores of each parameter, the acupuncture dose is classified into high, medium, and low levels. The assessment criteria are presented in Supplementary file 2.

### 2.6 Statistical analysis

The meta-analysis, publication bias assessment, subgroup analysis, and sensitivity analysis was performed using StataMP 18.0 software. Effect sizes were calculated using mean difference (MD) or standardized mean difference (SMD) and corresponding 95% confidence interval (CI) for continuous variables. Statistical significance was defined as *p* < 0.05. Heterogeneity of the included studies was evaluated using *I*^2^. When *I*^2^ ≤ 50%, it indicated acceptable heterogeneity and a fixed-effect model was used for meta-analysis; conversely, a random-effect model was used for meta-analysis and subgroup analysis was conducted (20). Sensitivity analysis was performed using leave-one-out. Publication bias was assessed using funnel plots and Egger’s test. The quality of evidence for the study results was evaluated using the GRADE guideline.

### 2.7 Risk of bias and quality assessment

The risk of bias and methodological quality of included studies were assessed using Review Manager (RevMan V.5.3) software, following the Cochrane Risk of Bias Tool which covers seven domains: random sequence generation, allocation concealment, blinding of participants and personnel, blinding of outcome assessors, incomplete outcome data, selective reporting, and other potential biases (21). Each domain was categorized into three risk levels: low risk of bias, unclear risk of bias, and high risk of bias. A study was defined as high-quality if all seven domains were rated as low risk of bias. Conversely, a study was considered to have low methodological quality if any domain was classified as unclear or high risk of bias.

### 2.8 Network meta-analysis

The network meta-analysis was conducted by using StataMP 18.0 software to compare the efficacy of different acupuncture types and doses for spasticity. A network plot with nodes and edges was constructed to visualize the included acupuncture types and doses, where node size was proportional to the total number of participants in the corresponding intervention, and edge thickness was proportional to the number of direct comparative studies between two interventions. Effect sizes were calculated as standardized mean differences (SMDs) with 95% confidence interval (CI). The relative efficacy of each acupuncture type and dose was ranked using the surface under the cumulative ranking curve (SUCRA). SUCRA values range from 0 to 100%: a higher SUCRA value indicates a greater probability of the intervention being the most effective, while a lower value indicates a lower probability of being the most effective (i.e., inferior therapeutic efficacy).

## 3 Results

### 3.1 Literature selection

**Figure 1:**
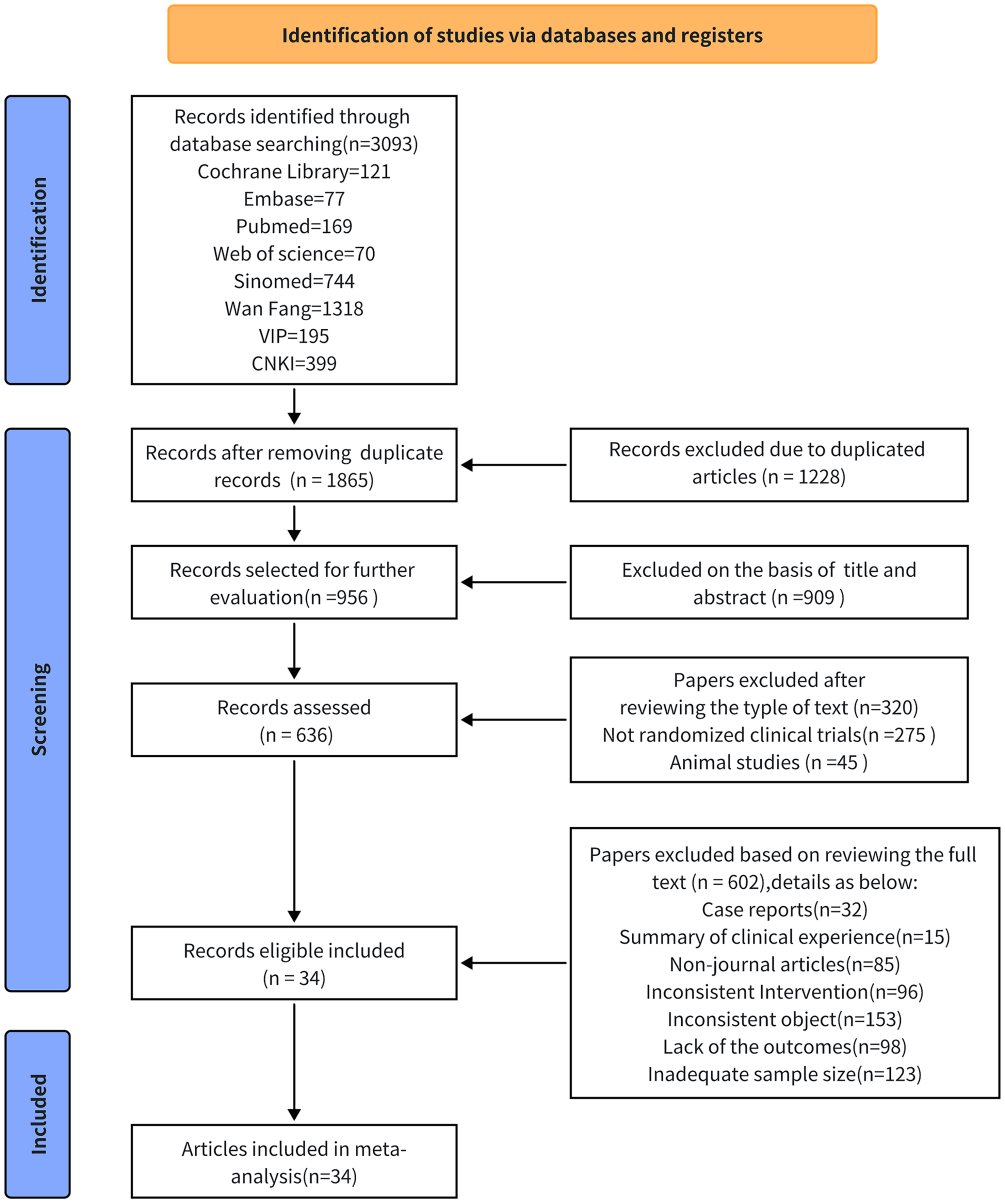
Flow chart of the literature screening process.

We initially retrieved 3,093 articles. After excluding 1,228 duplicate records and screening out 909 articles after reading title and abstract, 34 studies were ultimately included. The search flowchart is presented in Figure 1.

### 3.2 Characteristics of included literatures

Table 1 summarizes the baseline characteristics of the included studies. All included studies were conducted in China, among which 30 and 4 randomized controlled trials (RCTs) were published in Chinese and English, respectively, with publication years spanning from 2014 to 2024. A total of 3,383 participants were enrolled, including 1,693 in the experimental group and 1,690 in the control group, with a median sample size of 83 (IQR: 51–488). Twenty trials (22–41) reported stroke subtypes, with 1,313 patients diagnosed with cerebral infarction and 774 with intracerebral hemorrhage. The study participants were aged between 35 and 85 years, and the disease duration ranged from 72 h to 6 months. Acupuncture modalities included manual acupuncture (MA, 16 trials, *n* = 914), scalp acupuncture (SA, 5 trials, *n* = 226), fire acupuncture (FA, 3 trials, *n* = 123), electroacupuncture (EA, 5 trials, *n* = 219), and warm acupuncture (WA, 5 trials, *n* = 211). All trials reported Fugl-Meyer Assessment (FMA) scores, of which 28 trials (22–33, 35–39, 42–52) utilized the FMA-U score to evaluate upper limb motor function, and 22 trials (23, 25, 26, 28, 30, 31, 34–38, 40–42, 44–46, 48, 52–55) employed the FMA-L score to assess lower limb motor function. Sixteen trials assessed spasticity, of which 10 (29, 30, 32, 33, 37, 39, 43, 50, 51, 55) reported the Modified Ashworth Scale (MAS) scores and 6 trials (23, 25, 35, 48, 50, 54) reported the Clinical Spasticity Index (CSI) scores. Twenty-five trials (24, 27, 30–44, 47–49, 51–55) utilized the Modified Barthel Index (MBI) to evaluate the activities of daily living (ADL) of the study participants.

**Table 1:** Characteristic of included studies in meta-analysis.

Study	Participants, *n*		Mean age		Stroke subtypes		Disease course		Intervention	Comparison	Main outcomes	AEs
	TG	CG	TG	CG	IS	HS	TG	CG				
Chen et al. (42)	61	61	56.62 ± 5.68	55.21 ± 5.34	—		43.93 ± 8.87 (d)	42.34 ± 9.28 (d)	FA + RT	RT	FMA-U, FMA-L, MBI	—
Zhao and Zhang (22)	62	62	54.28 ± 19.72	54.12 ± 19.88	96	28	17.47 ± 5.53 (d)	17.55 ± 5.45 (d)	MA + RT	RT	FMA-U	—
Ma et al. (23)	42	42	61 ± 6	60 ± 6	63	21	27.8 ± 3.8 (d)	27.3 ± 3.6 (d)	MA + RT	RT	FMA-U, FMA-L, CSI	N
Xie et al. (43)	30	30	62.95 ± 3.36	63.37 ± 3.76	—		49.28 ± 12.49 (d)	46.22 ± 12.53 (d)	EA + RT	RT	FMA-U, MAS, MBI	—
Wang et al. (44)	90	90	35–85		—		72 (h)–6 (m)		MA + RT	RT	FMA-U, FMA-L, MBI	—
Sun et al. (24)	30	30	63 ± 6	64 ± 8	42	18	28.4 ± 5.6 (d)	29.1 ± 6.2 (d)	MA + RT	RT	FMA-U, MBI	N
Dai (25)	57	57	62.7 ± 8.6	62.9 ± 9.1	71	43	38.2 ± 10.4 (d)	36.7 ± 10.1 (d)	EA + RT	RT	FMA-U, FMA-L, CSI	—
Qiu et al. (45)	45	45	60.2 ± 10.4	59.6 ± 9.7	—		7.5 ± 1.2 (w)	7.2 ± 1.5 (w)	MA + RT	RT	FMA-U, FMA-L	—
Sun et al. (26, 63)	50	50	49.30 ± 10.61	50.40 ± 8.64	56	44	—		MA + RT	RT	FMA-U, FMA-L	N
Wang et al. (55)	30	29	56.7 ± 7.02	59 ± 7.51	—		59.53 ± 17.49 (d)	55.72 ± 15.78 (d)	MA + RT	RT	FMA-L, MAS, MBI	—
Xu et al. (46)	36	35	60 ± 10	65 ± 6	—		50.39 ± 22.52 (d)	47.75 ± 22.63 (d)	MA + RT	RT	FMA-U, FMA-L	N
Zhang et al. (13, 27, 59)	32	31	68 ± 8	66 ± 9	50	13	19.8 ± 3.8 (d)	21.6 ± 4.5 (d)	SA + RT	RT	FMA-U, MBI	N
Wang et al. (28)	30	30	54 ± 20	53 ± 21	46	14	253.4 ± 167.4 (d)	247.4 ± 171.1 (d)	FA + RT	RT	FMA-U, FMA-L	—
Zhang et al. (41)	70	70	51 ± 15	54 ± 16	78	62	36.22 ± 14.83 (d)	33.57 ± 14.76 (d)	SA + RT	RT	FMA-U, MAS, MBI	—
Han et al. (29)	244	244	51.2 ± 2.1	53.2 ± 1.9	325	163	13.3 ± 5.2 (d)	14.5 ± 5.0 (d)	MA + RT	RT	FMA-U, MAS	—
Tan (30)	44	44	54.93 ± 7.82	54.78 ± 7.69	42	39	44.86 ± 6.34 (d)	44.62 ± 6.28 (d)	MA + RT	RT	FMA-U, FMA-L, MAS, MBI	—
Chang et al. (47)	40	42	65.83 ± 6.20	65.65 ± 6.14	—		34.53 ± 5.67 (d)	34.65 ± 5.75 (d)	MA + RT	RT	FMA-U, MBI	—
Yin (48)	36	36	59.24 ± 5.71	58.83 ± 5.46	—		—		WA + RT	RT	FMA-U, FMA-L, CSI, MBI	—
Zhang et al. (40)	30	30	62.14 ± 8.03	59.36 ± 6.95	39	21	35.65 ± 10.31 (d)	37.20 ± 7.88 (d)	MA + RT	RT	FMA-L, MBI	—
Wang et al. (52, 69)	57	58	62.4 ± 9.0	59.5 ± 8.9	—		3.3 ± 1.6 (m)	3.0 ± 1.6 (m)	SA + RT	RT	FMA-U, FMA-L, MBI	3 cases of mild dizziness
Wang et al. (49)	44	44	61.87 ± 11.75	62.42 ± 12.37	—		2.27 ± 0.38 w	2.35 ± 0.43 w	MA + MT	MT	FMA-U, MBI	5 cases of mild discomfort
Wu (31)	32	33	60.33 ± 4.74	60.27 ± 4.78	17	48	—		FA + RT	RT	FMA-U, FMA-L, MBI	—
Zhu et al. (32)	42	41	59.2 ± 4.87	59.18 ± 4.66	65	18	87.36 ± 31.38 (d)	86.41 ± 30.28 (d)	MA + MT	MT	FMA-U, MAS, MBI	N
Qiu et al. (50)	45	45	62.31 ± 6.15	62.54 ± 6.28	—		1.85 ± 0.51 (y)	1.74 ± 0.46 (y)	MA + RT	RT	FMA-U, MAS, CSI	2 cases of hypersomnia, 2 cases of nausea and 1 case of pain
Liu et al. (33)	40	40	54 ± 12	55 ± 11	60	20	40.68 ± 10.01 (d)	38.34 ± 9.76 (d)	MA + RT	RT	FMA-U, MAS, MBI	N
Zhang et al. (39)	41	41	63.26 ± 2.56	63.15 ± 2.15	41	41	130.3 ± 14.5 (d)	133.5 ± 16.6 (d)	SA + RT	RT	FMA-L, MBI, Berg	1 case each of chest oppression, dizziness, and skin erythema and edema
Jia et al. (35)	26	25	63 ± 11	63 ± 11	38	13	41.4 ± 11.0 (d)	44.1 ± 13.2 (d)	SA + RT	RT	FMA-L, MBI, Berg	N
He (35)	47	47	51.1 ± 3.3	51.0 ± 3.3	53	41	1.54 ± 0.26 (m)	1.52 ± 0.27 (m)	EA + RT	RT	FMA-U, FMA-L, ADL, CSI, SF-36, NDS	—
Li et al. (36)	55	55	54.37 ± 5.63	53.86 ± 3.28	62	48	2.73 ± 0.65 (m)	2.62 ± 0.31 (m)	EA + RT	RT	FMA-U, FMA-L, ADL, MAS, sEMG	—
Liao (53)	30	30	65.18 ± 5.91		—		2.79 ± 1.28 (y)		EA + RT	RT	FMA-L, MBI, MAS	—
Rui (37)	35	33	58.6 ± 8.9	58.2 ± 9.4	33	35	3.3 ± 0.7 (m)	3.1 ± 0.8 (m)	WA + RT	RT	FMA-U, FMA-L, MBI, MAS	—
Zhu et al. (68)	60	60	65.0 ± 9.0	65.0 ± 8.0	—		33.0 ± 9.0 (d)	34.0 ± 7.0 (d)	WA + RT	RT	FMA-L, MBI, Berg, CSI, VEGF, Ang-1	—
Li and Ding (51)	40	40	54.21 ± 6.36	53.87 ± 6.23	—		61.3 ± 20.1 (d)	62.3 ± 20.1 (d)	WA + RT	RT	FMA-U, MAS, MBI, SSEP	—
Huang and Huang (38)	40	40	65.39 ± 7.34	67.79 ± 9.84	36	44	34.22 ± 12.10 (d)	37.91 ± 11.62 (d)	WA + RT	RT	FMA-U, FMA-L, MBI, SS-QOL	1 case of pain

TG, treatment group; CG, control group; IS, ischemic stroke; HS, hemorrhagic stroke; EA, electro-acupuncture; WA, warm acupuncture; SA, scalp acupuncture; MA, manual acupuncture; FA, fire acupuncture; RT, rehabilitation therapy; FMA-U, Fugl-Meyer Assessment of upper limb; FMA-L, Fugl-Meyer Assessment of lower limb; MAS, Modified Ashworth Scale; MBI, Modified Barthel Index; CSI, Clinic Spasticity Index; d, day; w, week; m, month; y, year; AEs, adverse events; 21. N, no reported.

### 3.3 Risk of bias assessment

#### 3.3.1 Random sequence generation

Thirty-four trials all reported the generation of random sequences. Among them, 32 trials (94.16%) were rated as low risk of bias (ROB) because they explicitly described the specific methods for random sequence generation: one trial employed random stratification (44), and 31 trials (22, 23, 25–31, 33–43, 45–55) utilized the random number table method. The remaining 2 trials (32, 45) were assessed as high risk due to the absence of any description regarding the random sequence generation process.

#### 3.3.2 Allocation concealment

Five trials (24, 26, 27, 39, 52) implemented allocation concealment and were thus rated as low ROB. In contrast, the remaining 29 trials (22, 23, 25, 28–38, 40–51, 53–55) did not report allocation concealment and were consequently assessed as unclear ROB.

#### 3.3.3 Blinding of participants with relevant personnel

Four trials (24, 26, 27, 52) were assessed as low ROB due to the implementation of blinding for participants, interveners, and outcome assessors. The remaining 30 trials (22, 23, 25, 28–51, 53–55) were rated as unclear ROB, as no mention was made of blinding for participants and outcome assessors.

#### 3.3.4 Complete outcome data and reporting bias

All 34 trials had complete outcome data with no missing values; therefore, selective reporting bias was assessed as low risk.

#### 3.3.5 Other bias

**Figure 2:**
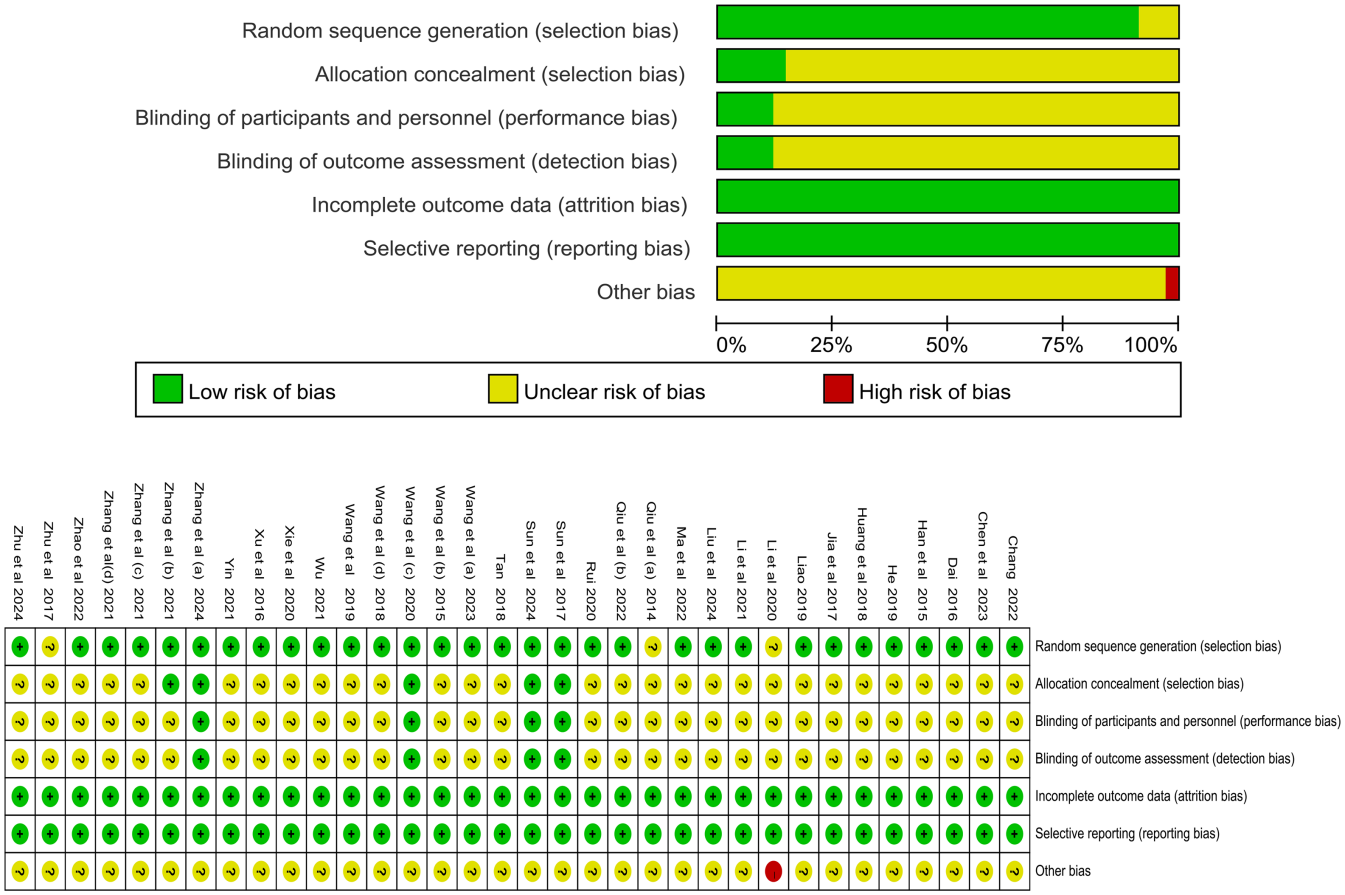
Risk of bias graph.

Given that the majority of the included trials were conducted in China and all reported positive outcomes, publication bias or language bias may exist due to the geographical concentration of the trials. Consequently, the 34 included trials were assessed as having unclear ROB, (see Figure 2).

### 3.4 Acupuncture dose evaluation

For all included trials, the treatment frequency ranged from once to 7 times per week, with 18 trials (24, 25, 27, 30, 34, 35, 38–40, 43–49, 52, 55) reported a frequency of 5 or 6 times weekly. The number of acupoints utilized varied from 4 to 17, and 15 trials (23, 26–28, 32, 33, 36–38, 43, 46–49, 55) used 9 to 15 acupoints. The overall treatment duration of the 34 trials ranged from 4 to 12 weeks, with 20 trials (22–28, 32, 34, 35, 38, 40, 42, 43, 46–50, 55) reported a treatment duration of 4 weeks. Twenty-six trials (22–28, 30, 32–38, 40, 43–47, 49, 50, 53–55) explicitly reported the “Deqi” response. The results of the acupuncture dose assessment indicated that 24 trials were classified as high-dose (70.59%), with the remaining 7 trials (20.59%) and 3 trials (8.82%) assessed as medium-dose and low-dose, respectively. For further details, see Supplementary file 3.

### 3.5 Efficacy evaluation

#### 3.5.1 Motor function

**Figure 3:**
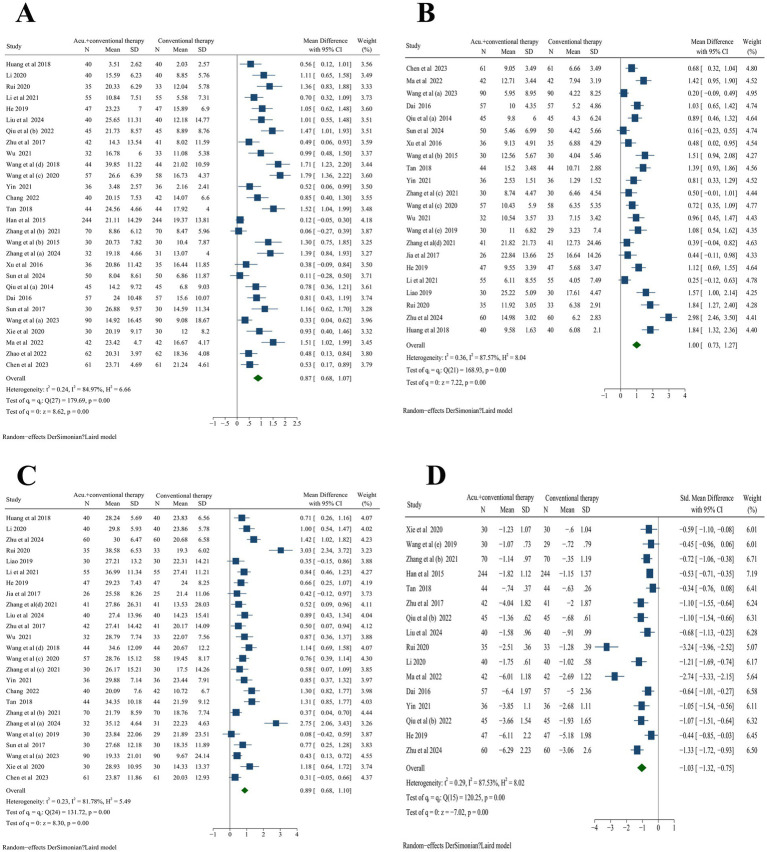
Efficacy evaluation. **(A)** FMA-U. **(B)** FMA-L. **(C)** MBI. **(D)** Spasticity.

Twenty-eight trials reported the FMA-U to evaluate upper limb motor function. A total of 2,951 patients were included for analysis, with 1,476 allocated to the acupuncture group and 1,475 to the conventional therapy group. Meta-analysis indicated that the acupuncture group demonstrated considerable improvements in FMA-U scores compared to conventional therapy group [MD = 0.87, 95% CI (0.68, 1.07), *p* < 0.001], with significant heterogeneity observed between the two groups (*I*^2^ = 84.97%, *p* < 0.001), (see Figure 3A). Twenty-two trials evaluate lower limb motor function using FMA-L scores, a total of 1,945 patients included, revealing a significant increase in FMA-L scores in the acupuncture group [MD = 1.00, 95% CI (0.73, 1.27), *p* < 0.001], with significant heterogeneity occurred between the two groups (*I*^2^ = 87.57%, *p* < 0.001), (see Figure 3B).

#### 3.5.2 Activities of daily living

Twenty-five trials used the MBI to evaluate activities of daily living (ADL), with a total of 2,162 patients included (acupuncture group: 1,082; conventional therapy group: 1,080). The results indicated that the acupuncture group exhibited a statistically significant improvement in MBI scores compared with the conventional treatment group [MD = 0.89, 95% CI (0.68, 1.10), *p* < 0.001], with considerable heterogeneity between the two groups (*I*^2^ = 81.78, *p* < 0.001), (see Figure 3C).

#### 3.5.3 Spasticity

Ten trials assessed spasticity using the MAS and the CSI, respectively, with a total of 1,810 patients included, with the acupuncture group (*n* = 907) and the conventional therapy group (*n* = 903). Given the inconsistency in measurement methods, the standardized mean difference (SMD) was used to quantify the effect size. Results indicated that the acupuncture group was significantly more effective in reducing spasticity compared with the conventional therapy group [SMD = −1.03, 95% CI (−1.32, −0.75), *p* < 0.001], with significant heterogeneity between the two groups (*I*^2^ = 87.53%, *p* < 0.001), (see Figure 3D).

#### 3.5.4 Subgroup analyses

Subgroup analyses were conducted based on the acupuncture types and doses, (see Supplementary files 4–6, Supplementary Figures S1–S9). For FMA-U, there were no significant differences in outcomes between the acupuncture types (EA vs. FA vs. MA vs. SA vs. WA, *p* = 1.00 for intergroup differences, Supplementary Figure S1) and doses (high vs. medium vs. low, *p* = 0.09 for intergroup differences, Supplementary Figure S5). For FMA-L, there were significant differences in outcomes among the acupuncture types (EA vs. FA vs. MA vs. SA vs. WA, *p* = 0.04 for intergroup differences, Supplementary Figure S2), but no significant differences among the doses (high vs. medium vs. low, *p* = 0.27 for intergroup differences, Supplementary Figure S6). For spasticity, there were significant differences in outcomes among the acupuncture types (*p* = 0.03 for intergroup differences, Supplementary Figure S4) and doses (*p* < 0.001 for intergroup differences, Supplementary Figure S8), but no significant differences among the assessment methods (CSI vs. MAS, *p* = 0.46 for intergroup differences, Supplementary Figure S9). For MBI, there were no significant differences in outcomes among the acupuncture types (EA vs. FA vs. MA vs. SA vs. WA, *p* = 0.40 for intergroup differences, Supplementary Figure S3) and doses (high vs. medium vs. low, *p* = 0.20 for intergroup differences, Supplementary Figure S7).

Subgroup analyses based on stroke type (ischemic vs. hemorrhagic), disease duration (<3 months vs. ≥3 months), and mean age (<60 years vs. ≥60 years) revealed that the *I*^2^ statistic remained high within each subgroup, ranging from 75 to 89%. This finding suggests that stratification by a single clinical characteristic is insufficient to account for the observed heterogeneity, which may reflect the inherent complexity of acupuncture intervention protocols (e.g., acupoint selection, stimulation parameters, treatment frequency) as well as individual variability in the pathophysiological mechanisms underlying PSS.

### 3.6 Safety evaluation

**Figure 4:**
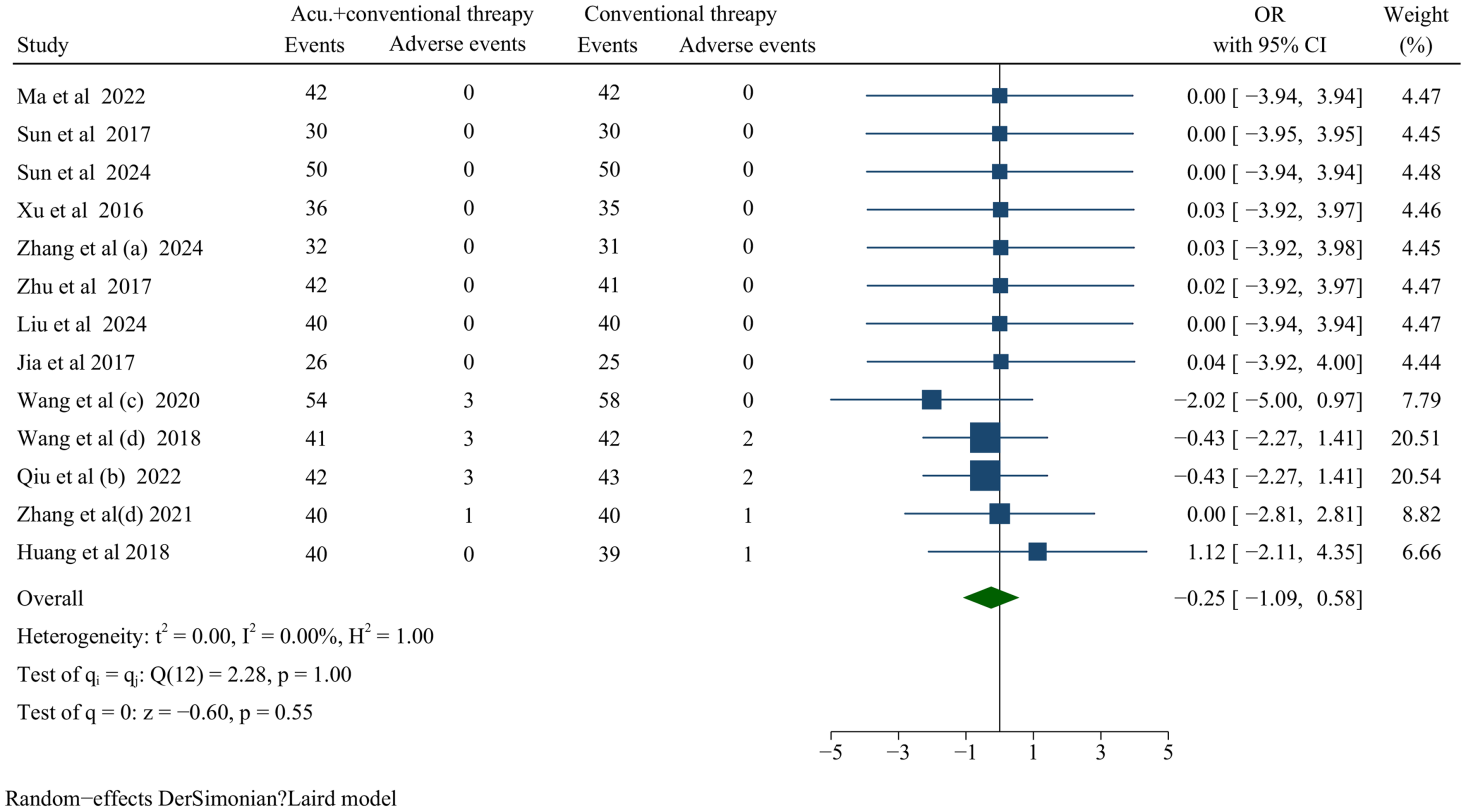
Safety evaluation.

Adverse events (AEs) were reported in 13 trials, with 8 trials explicitly noting the absence of any AEs throughout the study period. Among the remaining 5 trials, a total of 10 AEs occurred in the acupuncture group, including mild pain and dizziness, and these symptoms were well alleviated following rest. The conventional therapy group reported 6 AEs in total, including nausea, drowsiness, chest tightness, and dizziness. No statistically significant difference was observed in the rate of AEs between the two groups [OR = −0.25, 95% CI (−1.09, 0.58), *p* = 0.55], (see Figure 4).

### 3.7 Sensitivity analyses and publication bias

Leave-one-out sensitivity analysis revealed no significant changes in the overall effect sizes of FMA-U, FMA-L, spasticity, and MBI following the exclusion of any individual study (see Supplementary file 12, Supplementary Figures S26–S29). Funnel plots demonstrated missing studies on the left side of the FMA-U plot and on the right side of the FMA-L plot (see Supplementary file 13, Supplementary Figures S30, S32). Results of Egger’s test indicated potential small-sample effects for both FMA-U (*p* = 0.001) and FMA-L (*p* = 0.004). Trim-and-fill plots were applied to impute 7 studies for FMA-U and 4 studies for FMA-L, respectively (see Supplementary file 13, Supplementary Figures S31, S33). Funnel plots for MBI and spasticity were largely symmetrical, and Egger’s test did not detect small-sample effects for either outcome (MBI: *p* = 0.0973; spasticity: *p* = 0.0649) (see Supplementary file 13, Supplementary Figures S34–S37).

### 3.8 Quality of evidence

The certainty of evidence for acupuncture’s effects on FMA, MBI, and spasticity was systematically assessed using the GRADE system. Results demonstrated that the certainty of evidence across outcomes ranged from moderate to low ratings, of which only 1 outcome was graded as high certainty, 10 as moderate certainty, 10 as low certainty, and 12 as very low certainty. These findings primarily resulted from the methodological limitations in majority of studies, including publication bias, high statistical heterogeneity, and constraints in allocation concealment and blinding (see Supplementary file 14). Table 2 presents the quality assessment of each outcomes of included RCTs.

**Table 2:** Quality of evidence included RCTs by GRADE.

Outcomes	Number		Heterogeneity test results		Meta-analysis results		GRADE assessment
	Participants	Studies	*p*-value	*I* ^2^	Mean difference (MD)/Std. mean difference (SMD) and 95% CI	*p*-value	
FMA-U							
Type of acupuncture							
Electroacupuncture	378	4	*p* = 0.68	0.00%	MD = 0.85, 95% CI: (0.65, 1.06)	*p* < 0.001	High
Fire acupuncture	247	3	*p* = 0.05	65.99%	MD = 0.90, 95% CI: (0.44, 1.37)	*p* < 0.001	low
Manual acupuncture	1,708	14	*p* < 0.001	88.12%	MD = 0.83, 95% CI: (0.53, 1.13)	*p* < 0.001	Very low
Scalp acupuncture	318	3	*p* < 0.001	95.45%	MD = 1.07, 95% CI: (−0.08, 2.23)	*p* = 0.07	Moderate
Warm acupuncture	300	4	*p* = 0.04	63.78%	MD = 0.88, 95% CI: (0.48, 1.27)	*p* < 0.001	Low
Acupuncture dose							
High dose	1,851	20	*p* < 0.001	79.75%	MD = 0.82, 95% CI: (0.61, 1.03)	*p* < 0.001	Low
Medium dose	375	5	*p* = 0.63	0.00%	MD = 1.15, 95% CI: (0.94, 1.37)	*p* < 0.001	Moderate
Low dose	725	3	*p* < 0.001	96.00%	MD = 0.80, 95% CI: (−0.11, 1.70)	*p* = 0.08	Very low
FMA-L							
Type of acupuncture							
Electro-acupuncture	378	4	*p* < 0.001	83.46%	MD = 0.97, 95% CI: (0.44, 1.50)	*p* < 0.001	Very low
Fire acupuncture	247	3	*p* = 0.05	65.79%	MD = 1.01, 95% CI: (0.54, 1.48)	*p* < 0.001	Low
Manual acupuncture	732	8	*p* < 0.001	82.38%	MD = 0.75, 95% CI: (0.39, 1.11)	*p* < 0.001	Low
Scalp acupuncture	248	3	*p* = 0.48	0.00%	MD = 0.55, 95% CI: (0.30, 0.80)	*p* < 0.001	Moderate
Warm acupuncture	340	4	*p* < 0.001	91.81%	MD = 1.86, 95% CI: (0.96, 2.77)	*p* < 0.001	Very low
Acupuncture dose							
High dose	1,523	17	*p* < 0.001	89.95%	MD = 1.04, 95% CI: (0.69, 1.38)	*p* < 0.001	Low
Medium dose	185	3	*p* = 0.02	73.30%	MD = 1.00, 95% CI: (0.41, 1.59)	*p* < 0.001	Moderate
Low dose	237	2	*p* = 0.88	0.00%	MD = 0.70, 95% CI: (0.44, 0.96)	*p* < 0.001	Moderate
MBI							
Type of acupuncture							
Electro-acupuncture	324	4	*p* = 0.16	42.37%	MD = 0.75, 95% CI: (0.45, 1.05)	*p* < 0.001	Moderate
Fire acupuncture	187	2	*p* = 0.07	68.59%	MD = 0.56, 95% CI: (0.01, 1.10)	*p* = 0.05	Very low
Manual acupuncture	780	9	*p* < 0.001	70.54%	MD = 0.77, 95% CI: (0.50, 1.05)	*p* < 0.001	Low
Scalp acupuncture	451	5	*p* < 0.001	89.98%	MD = 0.92, 95% CI: (0.29, 1.55)	*p* < 0.001	Moderate
Warm acupuncture	420	5	*p* < 0.001	88.68%	MD = 1.36, 95% CI: (0.73, 2.00)	*p* < 0.001	Very low
Acupuncture dose							
High dose	1,520	17	*p* < 0.001	86.53%	MD = 0.99, 95% CI: (0.70, 1.29)	*p* < 0.001	Low
Medium dose	405	6	*p* = 0.47	0.00%	MD = 0.76, 95% CI: (0.56, 0.96)	*p* < 0.001	Moderate
Low dose	237	2	*p* = 0.08	66.41%	MD = 0.53, 95% CI: (0.08, 0.98)	*p* = 0.02	Very low
Spasticity							
Type of acupuncture							
Electro-acupuncture	268	3	*p* = 0.77	0.00%	SMD = −0.56, 95% CI: (−0.80, −0.32)	*p* < 0.001	Moderate
Manual acupuncture	1,062	8	*p* < 0.001	88.44%	SMD = −0.97, 95% CI: (−1.39, −0.56)	*p* < 0.001	Low
Scalp acupuncture	140	1	—	—	SMD = −0.72, 95% CI: (−1.06, −0.38)	*p* < 0.001	Low
Warm acupuncture	340	4	*p* < 0.001	89.21%	SMD = −1.66, 95% CI: (−2.42, −0.90)	*p* < 0.001	Very low
Acupuncture dose							
High dose	982	11	*p* < 0.001	90.21%	SMD = −1.11, 95% CI: (−1.55, −0.68)	*p* < 0.001	Very low
Medium dose	340	4	*p* = 0.39	1.34%	SMD = −1.01, 95% CI: (−1.24, −0.79)	*p* < 0.001	Moderate
Low dose	488	1	—	No show	SMD = −0.53, 95% CI: (−0.71, −0.35)	*p* < 0.001	Very low
Outcomes							
MAS	1,236	10	*p* < 0.001	86.21%	SMD = −0.94, 95% CI: (−1.28, −0.60)	*p* < 0.001	Very low
CSI	574	6	*p* < 0.001	89.23%	SMD = −1.19, 95% CI: (−1.73, −0.64)	*p* < 0.001	Very low

FMA-U, Fugl-Meyer Assessment of upper limb; FMA-L, Fugl-Meyer Assessment of lower limb; MAS, Modified Ashworth Scale; MBI, Modified Barthel Index; CSI, Clinic Spasticity Index; MD, mean difference; SMD, standardized mean difference.

### 3.9 Exploratory network meta-analysis

**Figure 5:**
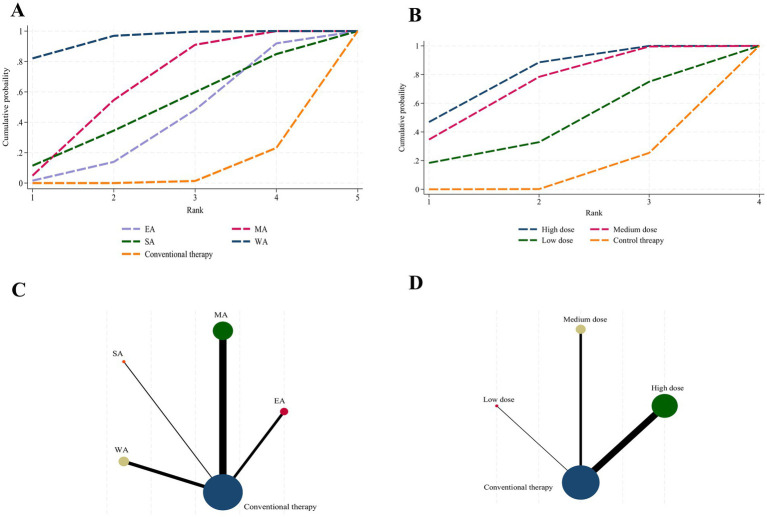
Network meta-analysis. **(A)** The SUCRA of acupuncture types. **(B)** The SUCRA values of acupuncture doses. **(C)** Network evidence plot of acupuncture types. **(D)** Network evidence plot of acupuncture doses.

The network evidence plot and the SUCRA probability rankings of different acupuncture types and doses for alleviating spasticity were shown in Figure 5. Among the acupuncture types, WA had the highest likelihood of being the optimal intervention, followed by MA, (see Figures 5A,C). For the three acupuncture dose, the high-dose showed the highest probability of being the optimal spasticity-alleviate dose, (see Figures 5B,D). SUCRA values estimated the probability of each acupuncture type and dose serving as the optimal intervention, with efficacy rankings as follows: WA (94.6%) > MA (62.6%) > SA (47.7%) > EA (38.9%) for types, and high dose (78.5%) > moderate dose (70.9%) > low dose (42.1%) for doses (see Supplementary files 15, 16). Beside, notably, the direct comparison of acupuncture types and doses with conventional therapy in the included studies impedes the construction of a closed-loop network, thereby preventing inconsistency testing.

## 4 Discussion

In this systematic review, we reviewed 34 RCTs evaluating acupuncture for PSS. Among these, 16 trials used MA, 5 used SA, 3 applied FA, 5 utilized EA, and 5 adopted WA. The results revealed that acupuncture therapy significantly improved scores on the FMA-U, FMA-L, and MBI, while reducing scores on the MAS and CSI for spasticity. These findings indicate that acupuncture may have superior effects in alleviating spasticity, enhancing motor function, and improving activities of daily living in patients with PSS. In terms of safety, the meta-analysis of 13 RCTs demonstrated no statistically significant difference in the incidence of AEs between groups. Notably, given that acupuncture-related AEs are generally minor and mild, acupuncture appears to be a relatively safer therapy for PSS. GRADE analysis revealed moderate-to-low evidence across the evaluated outcomes. Exploratory network meta-analysis identified WA and high-dose acupuncture (defined as ≥9 acupoints, ≥2 treatments per week, a treatment course of ≥8 weeks, and the requirement of “deqi” response) showed the highest probability of being optimal type and dose for alleviating spasticity respectively, however, the certainty of evidence remains limited. Taken together, these results held considerable implications for the clinical application of acupuncture in the management of PSS.

In recent years, acupuncture has gained growing attention in both research and clinical practice, with extensive applications in managing stroke-related sequelae such as post-stroke shoulder pain (56) and cognitive impairment (57), potentially attributable to its facilitation of neural plasticity within specific brain regions (58). The traditional Chinese medicine (TCM) believes that PSS is a complicated disease. Although previous studies have assessed the efficacy of acupuncture in PSS management and explored combinations of anti-spasticity acupoints, they have largely neglected the influence of acupuncture types and dose for PSS (12, 59). The absence of standardized protocols for acupoint selection, acupuncture types, and doses may poses significant challenges to the replication of clinical trials and hinders its broader acceptance as a mainstream therapy option. This study systematically evaluates the efficacy and safety of acupuncture for PSS and further exploring differential acupuncture types and doses for spasticity-alleviating. WA and MA demonstrated superior efficacy in alleviating spasticity (60, 61), with potential mechanisms involving upregulation of KCC2 expression and modulation of GABAAγ2 receptor activity (62), suppression of the NF-κB/NLRP3 signaling pathway, and attenuation of neuroinflammatory responses (63). Notably, WA combines the advantages of both acupuncture and moxibustion. By igniting moxa cones atop inserted needles, thermal energy is conducted deep into musculotendinous tissues, promoting the circulation of qi and blood, inducing local muscle relaxation, and thereby relieving regional spasticity (64). This finding may account for the more sustained and stable antispasticity effects with WA compared to MA alone.

The number of acupoint selection, treatment frequency, cumulative duration, and “deqi” response is another critical factor in acupuncture for PSS, closely linked to its promotion of motor networks and MR-driven sensorimotor integration (12, 65). These findings emphasized the importance of optimizing acupuncture dose to maximize neuroplastic changes and achieve clinically meaningful reductions in spasticity. The study revealed significant differences in spasticity improvement among high, medium, and low doses, with high-dose demonstrated superior efficacy in alleviating spasticity, reaching its maximum effect after at least 4 weeks of intervention, which aligns with the concept of acupuncture’s cumulative after-effect (66). Collectively, this study evaluate the role of acupuncture in PSS management, and to preliminarily summarize standardized acupuncture methods, providing practical references for patients and clinicians.

Previous studies have focused on optimizing combinations of antispasmodic acupoints to enhance the efficacy of acupuncture for PSS (67); however, no consensus has been reached regarding the optimal acupuncture modality and dosage for antispasmodic effects. A systematic review by Zhu et al. (68) revealed that EA combines warm acupuncture exhibited greater efficacy in spasticity reduction than traditional acupuncture [SMD = 1.35, 95% CI (0.57, 2.13) vs. SMD = 1.19, 95% CI (0.54, 1.83)]. In contrast, our study suggested that WA was the most effective in relieving spasticity, followed by MA (SCURA: 62.6%). This discrepancy may stem from three key factors: (1) differences in study populations (their study included mainly elderly patients, while ours covered both middle-aged and elderly patients); (2) variations in the number of included studies (4 vs. 16 in our study); and (3) differences in outcome measures (they used only MAS, while we conducted both MAS and CSI). Quantification of acupuncture interventions and establishment of standardized technical protocols may facilitate the transition of acupuncture therapy from empirical practice to evidence-based medicine (69). However, our study failed to find statistically significance between acupuncture types/doses and the FMA-U or MBI scores, indicating that the efficacy of acupuncture in improving motor function and activities of daily living (ADLs) may be relatively consistent. Relevant studies (7, 59, 70) have confirmed that acupuncture may facilitate or accelerate the recovery of post-stroke motor impairment and spasticity—findings consistent with the results of our study.

Our study had several strengths: (1) It further validates the efficacy and safety of acupuncture for PSS, with a focused on the effects of acupuncture types and doses on PSS, enhancing the clinical utility of the findings; (2) Compared with previous study, we found WA and high-dose showed the highest probability of being optimal intervention for spasticity; (3) A comprehensive literature search and data extraction were conducted across 8 databases, followed by rigorous evaluations of risk of bias, heterogeneity, and evidence quality; sensitivity analyses were additionally performed to confirm the robustness of the results.

This is an exploratory study rather than a confirmatory one. Thus, several limitations inherent to this study must be acknowledged. First, the geographic concentration of included trials (predominantly conducted in China, the origin of acupuncture) constrains the generalizability of the conclusions. Researchers in China may possess greater proficiency in acupoint selection, technical manipulation, and stimulation dosing; moreover, historical-cultural and genetic confounders may potentially influence patients’ acceptance of and adherence to acupuncture, elevating the risk of publication bias, the results of this article reflect the practice patterns, acupuncture techniques, and rehabilitation standards prevalent in China, and the observed rankings (e.g., WA as the highest-ranked intervention) should be interpreted within this context and may not be directly generalizable to treatment protocols or environment. Thus, cautious generalization of these findings to diverse populations is imperative. Second, implementing blinding in acupuncture trials presents inherent challenges: although scales such as the MAS, CSI, and MBI are widely used clinically, their assessment processes remain subjective (71); combined with potential placebo effects, this introduces biases that may compromise result reliability. Third, the heterogeneity of findings range from 75 to 89%, despite we have conducted subgroup analyses of relevant variables, which suggests that the diversity of acupuncture interventions (e.g., acupuncture techniques, retention time, cumulative dose) is a more dominant source of variation than patient-level factors. Besides, the limited number of studies reporting consistent covariates across all outcomes, along with insufficient data, precluded a reliable meta-regression analysis; performing it with the available data would risk spurious findings. Fourth, the certainty of evidence as rated by the GRADE is low, likely attributable to incomplete reporting of trial details (e.g., allocation concealment, blinding) in some studies. Finally, insufficient long-term follow-up data and in the included RCTs may hinder reliable conclusions of the long-term efficacy of acupuncture for PSS, and significant small-study effects (*p*_FMA-U_ = 0.001) and (*p*_FMA-L_ = 0.004) may could potentially overestimate treatment effects, which might influence the overall effect estimates.

Additionally, most of the included studies did not report baseline NIHSS scores, which limits our ability to directly assess the impact of stroke severity on treatment effects. However, all included studies were randomized controlled trials, and baseline FMA/MBI scores were comparable between groups in the studies that reported these measures, indirectly supporting baseline balance.

## 5 Conclusion

This meta-analysis demonstrates that acupuncture may exert a positive effect in alleviating spasticity, promoting motor function recovery and improving activities of daily living (ADLs), with considerable safety. WA and high-dose showed the highest probability of being optimal intervention for spasticity; however, the certainty of evidence remains limited. Further high-quality, large-sample RCTs are necessary to validate these findings and determine the optimal dose–response of acupuncture for PSS.

## Data Availability

The original contributions presented in the study are included in the article/Supplementary material, further inquiries can be directed to the corresponding author.
