# Supplementary material for: Acupuncture therapy for post-stroke spasticity: a systematic review and exploratory network meta-analysis of clinical efficacy and dose–response relationship
Source: Front Neurol. 2026 May 25;17:1782831. doi: 10.3389/fneur.2026.1782831 (PMC13243041; doi:10.3389/fneur.2026.1782831)
Supplement: Supplementary file 1 [file Data_Sheet_1.pdf]

Supplementary material

Table of contents

Table of contents 1
Supplemental file 1. Search strategies 3
Supplemental file 2. Quantitative assessment model of acupuncture dose 7
Table 1 Classification and Scoring System for Acupuncture dose 8
References： 9
Supplemental file 3. Quantitative evaluation form for acupuncture dose 10
Table 2. Summary of Acupuncture Dose Evaluation 10
Supplemental file 4.Subgroup analyses:Results stratified by acupuncture types 19
Figure S1. Mean effect sizes of FMA-U – under Electro-acupuncture vs. Fire acupuncture vs. Maunal acupuncture vs. Scalp acupuncture vs. Warm acupuncture 19
Figure S2. Mean effect sizes of FMA-L – under Electro-acupuncture vs. Fire acupuncture vs. Maunal acupuncture vs. Scalp acupuncture vs. Warm acupuncture 20
Figure S3. Mean effect sizes of MBI – under Electro-acupuncture vs. Fire acupuncture vs. Maunal acupuncture vs. Scalp acupuncture vs. Warm acupuncture 21
Figure S4. Standard mean effect sizes of Spasticity – under Electro-acupuncture vs. Maunal acupuncture vs. Scalp acupuncture vs. Warm acupuncture 22
Supplemental file 5.Subgroup analyses:Results stratified by acupuncture dose 23
Figure S5. Mean effect sizes of FMA-U – under High dose vs. Medium dose vs. Low dose 23
Figure S6. Mean effect sizes of FMA-L – under High dose vs. Medium dose vs. Low dose 24
Figure S7. Mean effect sizes of MBI – under High dose vs. Medium dose vs. Low dose 25
Figure S8. Standard mean effect sizes of Spasticity – under High dose vs. Medium dose vs. Low dose 26
Supplemental file 6.Subgroup analyses:Results stratified by Outcomes 27
Figure S9. Standard mean effect sizes of Spasticity – under MAS vs. CSI 27
Supplemnetal file 7 Subgroup analyses:Results stratified by Stroke subtypes 28
Figure S10. Standard mean effect sizes of FMA-U – under IS vs. IS+HS 28
Figure S11. Standard mean effect sizes of FMA-L – under IS vs. IS+HS 29
Figure S12. Standard mean effect sizes of MBI – under IS vs. IS+HS 30
Figure S13. Standard mean effect sizes of Spasticity – under IS vs. IS+HS 31
Supplemnetal file 8 Subgroup analyses:Results stratified by disease course 32
Figure S14. Standard mean effect sizes of FMA-U – 30~90 vs. ＞90 vs. ＜30 32
Figure S15. Standard mean effect sizes of FMA-L – 30~90 vs. ＞90 vs. ＜30 33
Figure S16. Standard mean effect sizes of MBI – 30~90 vs. ＞90 vs. ＜30 34
Figure S17. Standard mean effect sizes of Spasticity – 30~90 vs. ＞90 vs. ＜30 35
Supplemnetal file 9 Subgroup analyses:Results stratified by mean age 36
Figure S17. Standard mean effect sizes of FMA-U –＞60 vs. ＜60 36
Figure S18. Standard mean effect sizes of FMA-L –＞60 vs. ＜60 37
Figure S19. Standard mean effect sizes of MBI –＞60 vs. ＜60 38
Figure S20. Standard mean effect sizes of Spasticity –＞60 vs. ＜60 39
Supplemental file 10. Subgroup analyses:Results stratified by frequency (time/week) 40
Figure S21 Standard mean effect sizes of FMA-U with 3-5 times/week vs. 6-7 times/week 40

Figure S22 Standard mean effect sizes of FMA-L with 3-5 times/week vs. 6-7 times/week......41
Figure S22 Standard mean effect sizes of MBI with 3-5 times/week vs. 6-7 times/week......42
Figure S23 Standard mean effect sizes of Spasticity with 3-5 times/week vs. 6-7 times/week......43
Supplemental file 11. Subgroup analyses:Results stratified by course day (day)......44
Figure S24 Standard mean effect sizes of FMA-U with 20≤day<30 vs. 30≤day<40 vs. 40≤day......44
Figure S24 Standard mean effect sizes of FMA-L with 20≤day<30 vs. 30≤day<40 vs. 40≤day......45
Figure S25 Standard mean effect sizes of MBI with 20≤day<30 vs. 30≤day<40 vs. 40≤day......46
Figure S25 Standard mean effect sizes of Spasticity with 20≤day<30 vs. 30≤day<40 vs. 40≤day......47
Supplemental file 12 Sensitivity analyses......48
Figure S26. Sensitivity analysis of effect on FMA-U – leave-one-out analysis......48
Figure S27. Sensitivity analysis of effect on FMA-L – leave-one-out analysis......49
Figure S28. Sensitivity analysis of effect on MBI – leave-one-out analysis......50
Figure S29. Sensitivity analysis of effect on Spasticity – leave-one-out analysis......51
Supplemental file 13.Funnel plots and “trim and fill” plots......52
Figure S30.Fuuuel plot of the effect on FMA-U......52
Figure S31. Trim and fill plot for the effect on FMA-U......52
Figure S32.Fuuuel plot of the effect on FMA-L......53
Figure S33. Trim and fill plot for the effect on FMA-L......53
Figure S34.Fuuuel plot of the effect on MBI......54
Figure S35. Trim and fill plot for the effect on MBI......54
Figure S36.Fuuuel plot of the effect on Spasticity......55
Figure S37. Trim and fill plot for the effect on Spasticity......55
Supplemental file 14.Evaluation of Evidence Quality Based on GRADE......56
Table 3. Evaluation of the quality of research evidence based on the FMA-U......56
Table 4. Evaluation of the quality of research evidence based on the FMA-L......59
Table 5. Evaluation of the quality of research evidence based on the MBI......62
Table 6. Evaluation of the quality of research evidence based on the Spasticity......65
Supplemental file 15. The cumulative ranking curve rank by SCURA......68
Table 7. The SCURA based on different acupuncture types......68
Figure S38. Surface under the cumulative ranking curve rank for acupuncture types......68
Table 7. The SCURA based on different acupuncture dose......69
Figure S39. Surface under the cumulative ranking curve rank for acupuncture dose......69
Supplemental file 16. The League tables for the effect of acupuncture on spasticity with PSS patients......70
Supplemental file 17. Sensitivity analysis of effect on high-dose......71
Figure S40. Sensitivity analysis of effect on high-dose......71
Figure S41. Sensitivity analysis of effect on frequency≥2week......71
Figure S42. Sensitivity analysis of effect on sessions≥8times......72
Figure S43. Sensitivity analysis of effect on acupionts≥9......72
Figure S44. Sensitivity analysis of effect on Deqi......73

Supplemental file 1. Search strategies

Pubmed

#	Search strategies
#1	"Muscle Spasticity"[Mesh]
#2	(Hemiplegia, Spastic) OR (Spasticity after stroke)) OR (Spasticity Hemiplegia)) OR (Spastic Hemiplegias)) OR (poststroke spasticity)) OR (Limb spasm after storke)) OR (Muscle spasm after stroke) [Title/Abstract]
#3	#1 OR #2
#4	"Randomized Controlled Trials as Topic"[Mesh]
#5	(RCT) OR (Clinical Study)) OR (Clinical Trial) [Title/Abstract]
#6	#4 OR #5
#7	"Acupuncture"[Mesh]
#8	(Acupuncture Treatment) OR (Therapy, Acupuncture)) OR (Electroacupuncture)) OR (Warm needle)) OR (Fire needle)) OR (Manual acupuncture)) OR (Scalp acupuncture)) OR (Sham acupuncture)) OR (wrist-ankle acupuncture)) OR (Abdominal needle)) OR (Eye acupuncture)) OR) [Title/Abstract]
#9	#7 OR #8
#10	#3 AND #6 AND #9

Embase

#	Search strategies
#1	' spasticity after stroke '/exp OR ' spasticity hemiplegia '
#2	' hemiplegia, spastic ':ab,ti OR ' muscle spasticity ':ab,ti OR ' spastic hemiplegias ':ab,ti OR ' poststroke spasticity ':ab,ti OR ' limb spasm after storke ':ab,ti OR ' muscle spasm after stroke ':ab,ti
#3	#1 OR #2
#4	'acupuncture'/exp OR 'acupuncture treatment'
#5	'acupuncture therapy':ab,ti OR 'needle therapy':ab,ti OR 'electroacupuncture':ab,ti OR ' scalp acupuncture ':ab,ti OR ' manual acupuncture ':ab,ti OR 'warm acupuncture':ab,ti OR 'abdominal acupuncture':ab,ti OR ' sham acupuncture ':ab,ti OR ' wrist-ankle acupuncture ':ab,ti OR ' fire acupuncture ':ab,ti
#6	#4 OR #5
#7	' randomized controlled trial '/exp OR ' RCT '
#8	' clinical study ':ab,ti OR 'randomized':ab,ti OR ' clinical trial ':ab,ti
#9	#7 OR #8
#10	#3 AND #6 AND #9

Cochrane Library

#	Search strategies
#1	spasticity after stroke
#2	(spasticity hemiplegia):ti,ab,kw OR (hemiplegia, spastic):ti,ab,kw OR (spastic hemiplegias):ti,ab,kw OR (poststroke spasticity):ti,ab,kw OR (limb spasm after storke):ti,ab,kw OR (muscle spasm after stroke):ti,ab,kw OR (muscle spasticity):ti,ab,kw
#3	#1 OR #2
#4	acupuncture
#5	(acupuncture therapy):ti,ab,kw OR (electroacupuncture):ti,ab,kw OR (scalp acupuncture):ti,ab,kw OR (manual acupuncture):ti,ab,kw OR (warm acupuncture):ti,ab,kw OR (abdominal acupuncture):ti,ab,kw OR (acupuncture treatment):ti,ab,kw OR (fire acupuncture):ti,ab,kw OR (wrist-ankle acupuncture):ti,ab,kw OR (sham acupuncture):ti,ab,kw
#6	#4 OR #5
#7	(randomized controlled trial):ti,ab,kw OR (randomized):ti,ab,kw OR (clinical study):ti,ab,kw OR(RCT): ti,ab,kw OR(clinical trial) : ti,ab,kw OR (RCT):ti,ab,kw
#8	#3 AND #6 AND #7

Web of Science

#	Search strategies
#1	TS=(spasticity after stroke)
#2	TS=(spasticity hemiplegia) OR TS=(spastic hemiplegias) OR TS=(poststroke spasticity) OR TS=(limb spasm after storke) OR TS=(muscle spasm after stroke) OR TS=(muscle spasticity) and Preprint Citation Index (Exclude – Database)
#3	#1 OR #2
#4	TS= (acupuncture)) OR TS= (acupuncture therapy)
#5	TS=(electroacupuncture) OR TS=(scalp acupuncture) OR TS=(manual acupuncture) OR TS=(warm acupuncture)) OR TS=(abdominal acupuncture) OR TS=(abdominal acupuncture)) OR TS=(fire acupuncture) OR TS=(wrist-ankle acupuncture) OR TS=(sham acupuncture) OR TS=(acupuncture treatment)and Preprint Citation Index (Exclude-Database)
#6	#4 OR #5
#7	TS=(randomized controlled trial)
#8	TS=(randomized) OR TS=(RCT) OR TS=(clinical study) OR TS=(clinical trial) and Preprint Citation Index (Exclude – Database)
#9	#7 OR #8
#10	#3 AND #6 AND #9

frontiers

CNKI

#	Search strategies
#1	篇关摘=中英文拓展（中风后痉挛性偏瘫）
#2	篇关摘=中英文拓展（痉挛性偏瘫）
#3	篇关摘=中英文拓展（卒中）
#4	篇关摘=中英文拓展（中风）
#5	篇关摘=中英文拓展（肢体痉挛）
#6	篇关摘=中英文拓展（上肢痉挛）
#7	篇关摘=中英文拓展（下肢痉挛）
#8	篇关摘=中英文拓展（肌张力增高）
#9	篇关摘=中英文拓展（中风后痉挛）
#10	#1 OR #2 OR #3 OR #4 OR #5 OR #6 OR #7 OR #8 OR #9
#11	篇关摘=中英文拓展（针刺疗法）
#12	篇关摘=中英文拓展（针刺治疗）
#13	篇关摘=中英文拓展（头针）
#14	篇关摘=中英文拓展（火针）
#15	篇关摘=中英文拓展（温针）
#16	篇关摘=中英文拓展（电针）
#17	篇关摘=中英文拓展（手针）
#18	篇关摘=中英文拓展（假针）
#19	篇关摘=中英文拓展（安慰针）
#20	篇关摘=中英文拓展（针刺）
#21	#11 OR #12 OR #13 OR #14 OR #15 OR #16 OR #17 OR #18 OR #19 OR #20
#22	篇关摘=中英文拓展（随机对照试验）
#23	篇关摘=中英文拓展（临床研究）
#24	篇关摘=中英文拓展（临床试验）
#25	篇关摘=中英文拓展（RCT）
#26	#22 OR #23 OR #24 OR #25
#27	#10 AND #21 AND #26

VIP

#	Search strategies
#1	题名或关键词=中风后痉挛性偏瘫 OR 痉挛性偏瘫 OR 卒中 OR 中风 OR 肢体痉挛 OR 中风后痉挛 OR 上肢痉挛 OR 下肢痉挛 OR 肌张力增高 OR 痉挛性运动障碍
#2	题名或关键词=针刺疗法 OR 针刺治疗 OR 头针 OR 火针 OR 温针 OR 电针 OR 手针 OR 针刺 OR 假针 OR 安慰针
#3	题名或关键词=随机对照试验 OR 临床研究 OR 临床试验 OR RCT
#4	#1 AND #2 AND #3

WanFang

#	Search strategies
#1	主题=中风后痉挛性偏瘫 OR 痉挛性偏瘫 OR 卒中 OR 中风 OR 肢体痉挛 OR 中风后痉挛 OR 上肢痉挛 OR 下肢痉挛 OR 肌张力增高 OR 痉挛性运动障碍[智能：中英文拓展/主题词拓展]
#2	主题=针刺疗法 OR 针刺治疗 OR 头针 OR 火针 OR 温针 OR 电针 OR 手针 OR 针刺 OR 假针 OR 安慰针[智能：中英文拓展/主题词拓展]
#3	主题=随机对照试验 OR 临床研究 OR 临床试验 OR RCT[智能：中英文拓展/主题词拓展]
#4	#1 AND #2 AND #3

SinoMed

#	Search strategies
#1	“中风后痉挛性偏瘫”[常用字段:智能] OR “痉挛性偏瘫”[常用字段:智能] OR “卒中”[常用字段：智能] OR “中风”[常用字段：智能] OR “肢体痉挛”[常用字段：智能] OR “中风后痉挛”[常用字段：智能] OR “上肢痉挛” [常用字段：智能]OR “下肢痉挛”[常用字段：智能] OR “肌张力增高”[常用字段：智能] OR “痉挛性运动障碍”[常用字段：智能]
#2	“针刺疗法”[常用字段：智能] OR “针刺治疗”[常用字段：智能] OR “头针”[常用字段：智能] OR “火针”[常用字段：智能] OR “温针”[常用字段：智能] OR “电针”[常用字段：智能] OR “手针”[常用字段：智能] OR “针刺”[常用字段：智能] OR “假针”[常用字段：智能] OR “安慰针”[常用字段：智能]
#3	“随机对照试验”[常用字段：智能] OR “临床研究” [常用字段：智能] OR “临床试验” [常用字段：智能] OR “RCT” [常用字段：智能]
#4	#1 AND #2 AND #3

Supplemental file 2. Quantitative assessment model of acupuncture dose

The dose of acupuncture is considered to be a significant factor influencing the clinical efficacy of acupuncture, encompassing elements such as the number of treatments, the number of acupoints, the location of the needle insertion, and the intensity of stimulation [1]. In accordance with the Chinese standard clinical practice, Sun N et al. [2] have proposed four parameters for calculating the dose of acupuncture: (1) the number of acupoints stimulated during each treatment session; (2) the occurrence of "De qi" sensation; (3) Additionally, the number of treatments per week was considered, as well as the number of treatments. The term "De qi" is a necessary condition for the generation of analgesia or therapeutic effects and can be used to quantify the intensity of the stimulus[3]. Furthermore, Sun N et al. proposed that the median dose be taken as the mode of each parameter value, with each parameter defined as a high dose requiring at least: (1) nine acupoints; or (2) The occurrence of a sensation of numbness; or (3) a minimum of two treatments per week; or (4) a minimum of eight treatments in total. If the aforementioned levels are not reached, the parameter is defined as a low dose.

A rating system based on high/low (high/low) dose classification for the four parameters was constructed to determine the overall dose of whole-body acupuncture. Each high-dose parameter A score of +1 was allocated for each high-dose parameter, while a score of -1 was allocated for each low-dose parameter. Based on the total score, three categories of acupuncture treatment dose were defined: high dose (range 1 to 4), moderate dose (score 0) and low dose (-4 to -1). These are summarised in **Table 1**.

Table 1 Classification and Scoring System for Acupuncture dose

Parameter	Classification Criteria	Dosage Classification	Score	Total Score Range	Acupuncture Treatment Dosage
Number of Acupoints	≥9 acupoints	High dose	+1		
	<9 acupoints	Low dose	-1		
Deqi Response	Reported	High dose	+1		
	Not reported	Low dose	-1		
Treatment Frequency/Week	≥2 times/week	High dose	+1		
	<2 times/week	Low dose	-1		
Total Treatment Sessions	≥8 sessions	High dose	+1		
	<8 sessions	Low dose	-1		
**Total Score Range**				1 ~ 4	High dose
				0	Moderate dose
				-1 ~ -4	Low dose

***Note**: Each parameter is assigned a score of +1 or -1 depending on whether it meets the high-dose conditions. The total score is the sum of the four parameter scores and is used to determine the overall dosage level of the acupuncture treatment.

References:

[1] Yoon DE, Lee IS, Chae Y. Identifying Dose ComPonents of Manual AcuPuncture to Determine the Dose-Response Relationship of AcuPuncture Treatment: A Systematic Review. Am J Chin Med. 2022;50(3):653-671.

[2] Sun N, Tu JF, Lin LL, et al. Correlation between acuPuncture dose and effectiveness in the treatment of knee osteoarthritis: a systematic review. AcuPunct Med. 2019;37(5):261-267.

[3] Kong, J., R. Gollub, T. Huang, G. Polich, V. Napadow, K. Hui, M. Vangel, B. Rosen and T.J. Kaptchuk. Acupuncture de qi, from qualitative history to quantitative measurement. J. Altern. Complement. Med. 13: 1059–1070, 2007.

Supplemental file 3. Quantitative evaluation form for acupuncture dose

According to Supplemental file 2， The quantitative assessment of acupuncture dose for the 34 included trials in terms of treatment frequency/week, total treatment sessions, number of acupoints, and Deqi response,above of which was summarized in **Table 2**

Table 2. Summary of Acupuncture Dose Evaluation

Researchers, year	Participants TG	Participants CG	Frequency	Duration	Acupoints	Acupoint counts	De qi	Total score	Dose level
Chen et al 2023	61	61	Every other day, 1 session	4 weeks	Shoulder, elbow, knee, ankle joints around "Ah-Shi" points	8~10	/	-1	Low dose
Zhao et al 2022	62	62	Daily, 1 session, 30 minutes each	30 days	Jiquan(HT1)，Neiguan(PC6)，Chize(LU5)，Shuigou(DU26)，Yangchi(SJ4)	5	Yes	2	High dose
Ma et al 2022	42	42	Daily, 1 session, 30 minutes each	4 weeks	Jianliao(SJ14)，Tianjing(SJ10), Waiguan(SJ5), Yangchi(SJ4)，Shenmai(BL62)，Houxi(SI3)，Huantiao(GB30)，Chengshan(BL57)，Yanglingquan(GB34)，Qiu Xu(GB40)，Xuanzhong(GB39)	11	Yes	2	High dose
Xie et al 2020	30	30	Retain needle for 30 minutes, daily, 1 session, 6 times per week	4 weeks	Jianyu (LI15)，Jianliao (SJ14)，Naohui (SI10)，Qinglengyan (SI8)，Quchi (LI11)，Waiguan (SJ5)，Hegu (LI4)，Xuehai (SP10)，Liangqiu (ST34)，Zusanli	13	Yes	2	High dose

Researchers, year	Participants TG	CG	Frequency	Duration	Acupoints	Acupoint counts	De qi	Total score	Dose level
					(ST36)，Sanyinjiao (SP6)，Kunlun (BL60)，Taichong (LR3)				
Wang et al (a) 2023	90	90	30 minutes per session, daily, 1 session, 5 times per week	8 weeks	Dingzhongxian (EX-HN1)，Dingnieqianxieyan (EX-HN4)，Neiguan (PC6)，Zusanli (ST36)，Shuigou (DU26)，Sanyinjiao (SP6)，Chize (LU5)，Weizhong (BL40)	8	Yes	2	High dose
Sun et al 2017	30	30	Daily, 1 session, 5 times per week	4 weeks	Zhongfu (LU1)，Tianfu (LU3)，Chize (LU5)，Quchi (LI11)，Jianshi (PC5)，Daleng (PC7)	6	Yes	0	Medium dose
Dai 2016	57	57	30 minutes per session, daily, 1 session, 6 times per week	4 weeks	Jiquan (HT1)，Quchi (LI11)，Neiguan (PC6)，Shousanli (LI10)，Daleng (PC7)，Hegu (LI4)，Houxi (SI3)，Jìmài (EX-LE7) ，Yinlingquan (SP9)，Weizhong (BL40)，Sanyinjiao (SP6)，Fenglong (ST40)，Shangqiu (SP5)，Taichong (LR3)，Xiexi (ST41)，Qiuxu (GB40)	16	Yes	2	High dose
Qiu et al (a) 2014	45	45	30 minutes per session, daily, 1 session, 6 times per week	8 weeks	Baihui (DU20)，Sishencong (EX-HN1)，Fengchi (GB20)，Jianyu (LI15)，Binao (LI15)，Quchi (LI11)，Shousanli (LI10)，Waiguan	17	Yes	4	High dose

Researchers, year	Participants TG	CG	Frequency	Duration	Acupoints	Acupoint counts	De qi	Total score	Dose level
					(SJ5)，Hegu (LI4)，Huantiao (GB30)，Fengshi (GB31)，Weizhong (BL40)，Zusanli (ST36)，Yanglingquan (GB34)，Sanyinjiao (SP6)，Xuanzhong (GB39)，Taichong (LR3)				
Sun et al 2024	50	50	Weekly, 3 times	4 weeks	Quchi (LI11)，Shousanli (LI10)，Hegu (LI4)，Yanglingquan (GB34)，Xuanzhong (GB39)，Jianliao (SJ14)，Waiguan (SJ5)，Sanyinjiao (SP6)，Taixi (KI3)	9	Yes	2	High dose
Wang et al 2019	30	29	6 consecutive sessions per week for 4 weeks	4 weeks	Baihui(GV20) ，Taiyang (EX-HN5)，Yinmen (BL37)，Fuxi (BL38)， Xiyangguan(GB33)，Yanglingquan (GB34)， Zusanli (ST36)， Tiaokou (ST38)，Taichong (LR3)	9	Yes	2	High dose
Xu et al 2016	36	35	Daily, 1 session, 5 times per week	4 weeks	Jiquan (HT1)，Chize (LU5)，Neiguan (PC6)，Yinlingquan (SP9)，Sanyinjiao (SP6)，Yangxi (LI5)，Yangchi (SJ4)，Daleng (PC7)，Shuigou (DU26)，Yongquan (KI1)，Zhongchong (HT9)	11	Yes	2	High dose

Researchers, year	Participants TG	Participants CG	Frequency	Duration	Acupoints	Acupoint counts	De qi	Total score	Dose level
Zhang et al (a) 2024	32	31	Daily, 1 session, 6 times per week	4 weeks	Baihui (DU20)，Fengfu (DU16)，Dazhui (DU14)，Taodao (DU13)，Shen Zhu (DU12)，Zhiyang (DU9)，Mingmen (DU4)，Yaoyangguan (DU3)，C4-T1 Jiaji (EX-B2)	9~16	Yes	2	High dose
Wang et al (b) 2015	30	30	Every other day, 1 session, 15 treatments	30 days	Jianyu (LI15)，Binao (LI15)，Quchi (LI11)，Shousanli (LI10)，Waiguan (SJ5)，Yangchi (SJ4)，Houxi (SI3)， Hegu (LI4)，Baxie (EX-UE11) ， Yanglingquan (GB34)，Xuehai (SP10)，Sanyinjiao (SP6)，Yingu (KI2)，Taixi (KI3)，Zuqiaoyin(GB44)	15	Yes	2	High dose
Zhang et al (b) 2021	70	70	Daily, 1 session, 6 times per week	8 weeks	Sishencong (EX-HN1)，Xuanli (EX-HN5)，Baihui (DU20)，Qubin (GB1)	4	/	1	High dose
Han et al 2015	244	244	Daily, 1 session, 7 times per week	3 weeks	Jianjing (GB21)， Jianliao (SJ14)，Jianzhen (SI9)，Points of the Three Yang Meridians of the Hand	3~9	/	-1	Low dose
Tan 2018	44	44	Daily, 1 session, 6 times per week	12 weeks	Jianyu (LI15)，Binao (LI15)，Quchi (LI11)，Shaohai (HT3)，Waiguan (SJ5)，Neiguan (PC6)，Hegu (LI4)，Houxi (SI3)，	16	Yes	4	High dose

Researchers, year	Participants TG	Participants CG	Frequency	Duration	Acupoints	Acupoint counts	De qi	Total score	Dose level
					Yanglingquan (GB34)，Yinlingquan (SP9)，Juegu (GB39) ，Sanyinjiao (SP6)，Kunlun (BL60)，Taixi (KI3)，Taichong (LR3)，Yongquan (KI1)				
Chang 2022	40	42	Daily, 1 session, 5 times per week	4 weeks	Zhiyang (GV9)，Baihui(GV20)，Weizhong (BL40)，Chize (LU5)，Zhaohai(KI6)，Xiaohai(SI8)，Shenting (GV24)，Chengshan (BL57)，Yinlingquan (SP9)，Ximen (PC4)，Ximen (PC4)	11	Yes	3	High dose
Yin 2021	36	36	Daily, 1 session, 5 times per week	4 weeks	Jianqian(LI14)，Neiguan(PC6)，Quchi(LI11)，Hegu(LI4)，Chize(LU5)，Shousanli (LI10)，Fengshi(GB31)，Huanzhao(GB30)，Taichong(LR3)，Xuehai(SP10)，Qiuxu(GB40)，Sanyinjiao(SP6)，Yinlingquan (SP9)	13	/	1	High dose
Zhang et al (c) 2021	30	30	Daily, 2 sessions, 6 times per week	4 weeks	Baihui(GV20)，Bijuan(ST31)，Xuehai(SP10)，Yanglingquan(GB34)，Xuanzhong(GB39)，Jiexi(ST41)，Qiuxu(GB40)	7	Yes	0	Medium dose

Researchers, year	Participants TG	CG	Frequency	Duration	Acupoints	Acupoint counts	De qi	Total score	Dose level
Wang et al (c) 2020	57	58	Daily, 6 times per week	2 weeks	Bilateral anterior oblique line of vertex-temporal(MS6) ，Baihui(GV20)，Qianshencong (EX-HN1)，Xuanli (GB8)，Qianding (GV21)	4~7	/	-1	Low dose
Wang et al (d) 2018	44	44	Daily, 1 session, 6 times per week	4 weeks	Jianyu (LI15)，Jianliao (SJ14)，Shousanli (LI10)，Quchi (LI11)，Waiguan (SJ5)，Hegu (LI4)，Taichong (LR3)，Fenglong (ST40)，Zusanli (ST36)，Taixi (KI3)	10	Yes	2	High dose
Wu 2021	32	33	Every 10 minutes, every other day, 20 treatments	40 days	Jianliao(SJ14)，Binao(LI15)，Quchi (LI11)，Shousanli(LI10)，Waiguan(SJ5)，Hegu (LI4)，Huantiao(GB30), Biguan(ST31)，Fengshi(GB31)，Xiyangyan(EX-LE7)，Ququan(LR8)，Yanglingquan(GB34)，Sanyinjiao (SP6)，Zusanli (ST36)，Taichong (LR3)，Jiexi (ST41)	16	/	0	Medium dose
Zhu et al 2017	42	41	Every 10 minutes, every other day, 20 treatments	4 weeks	Neiguan(PC6)，Chize(LU5)，Jiquan(HT1)，Daling(PC7)，Chengshan(BL57)，Xuehai(SP10)，Futu(ST32) ，	15	Yes	2	High dose

Researchers, year	Participants TG	Participants CG	Frequency	Duration	Acupoints	Acupoint counts	De qi	Total score	Dose level
					Sanyinjiao(SP6)，Hegu(LI4)，Shousanli(LI10)，Naohui(TE14)，Waiguan(SJ5)，Yanglingquan(GB34)，Yinmen(BL37)， Jiexi(ST41)				
Qiu et al (b) 2022	45	45	Every 10 minutes, every other day, 20 treatments	30 days	Yangming Meridian Points，Yanglingquan(GB34)，Xuanzhong(GB39)，Zusanli (ST36)	4~10	Yes	0	Medium dose
Liu et al 2024	40	40	Daily, 1 session, 10 days per course	22 days	Jianliao(SJ14)，Binao(LI15)，Qinglengyuan(SI8)，Tianjing(TE10)，Shousanli(LI10)，Waiguan(SJ5)，Baxie(EX-UE11)，Tianfu(LU3)，Jianzhen(SI9)，Chize(LU5)，Daling (PC7)	11	Yes	0	Medium dose
Zhang et al(d) 2021	41	41	30 min each time, every other day	12w	anterior oblique line of vertex-temporal（the oblique line from Qianshencong (1 cun anterior to Baihui GV20) to Xuanli (GB6)），posterior oblique line of vertex-temporal（the oblique line from Baihui (GV20) to Qubin (GB7)）	4~6	/	1	High dose
Jia et al 2017	26	25	40 min each time, once daily, 5 times per week	4w	anterior oblique line of vertex-temporal（the oblique line from Qianshencong (1 cun anterior to	4~6	Yes	2	High dose

Researchers, year	Participants TG	CG	Frequency	Duration	Acupoints	Acupoint counts	De qi	Total score	Dose level
					Baihui GV20) to Xuanli (GB6)）, posterior oblique line of vertex-temporal（the oblique line from Baihui (GV20) to Qubin (GB7)）				
He 2019	47	47	30 min each time, 6 times per week	4w	Baihui(GV20)，Taiyang(EX-HN5)，Fengchi(GB20)，Fengfu(GV16)，Jiquan(HT1)，Quchi(LI11)，Neiguan(PC6)，Shousanli(LI10)，Daling(PC7)，Hegu (LI4)，Houxi(SI3)，Yinlingquan(SP9)，Weizhong (BL40)，Sanyinjiao(SP6)，Fenglong(ST40)，Shangqiu(SP5)，Taichong(LR3)，Jiexi(ST41)，Qiuxu(GB40)	19	Yes	4	High dose
Li et al 2021	55	55	30 min each time, once daily, 10 days per course	6w	Jiquan(HT1)，Waiguan(TE5)，Hegu(LI4)，Quchi(LI11)，Shousanli(LI10)，Daleng(PC7)，Huantiao(GB30)，Ququan(LR8)，Zusanli(ST36)，Sanyinjiao(SP6)，Taichong(LR3)	11	Yes	4	High dose
Liao 2019	30	30	30 min each time, once daily, 10 days per course	40d	Chengfu(BL37)，Yinmen(BL39)，Yinlingquan(SP9)，Zhaohai(KI6)	4	Yes	0	Medium dose

Researchers, year	Participants TG	CG	Frequency	Duration	Acupoints	Acupoint counts	De qi	Total score	Dose level
Rui 2020	35	33	20 min each time, once daily, 10 days per course	30d	Baihui(GV20)，Hegu(LI4)，Quchi(LI11)，Neiguan(PC6)，Waiguan(TE5)，Chize(LU5)，Weizhong(BL40)，Zusanli(ST36)，Yanglingquan(GB34)	9	Yes	4	High dose
Zhu et al 2024	60	60	30 minutes each time, 4 times per week	5w	Fengshi(GB31)，Taichong(LR3)，Huantiao(GB30)，Xuehai(SP10)，Yanglingquan(GB34)，Qiuxu(GB40)	6	Yes	2	High dose
Li et al 2020	40	40	45 minutes each time,once daily, 12 days per course	5w	Waiguan(TE5)，Jian Yu (LI15)，Shousanli(LI10)，Quchi(LI11)	4	/	0	Medium dose
Huang et al 2018	40	40	30 minntes each time, once daily, 5 times a week	4w	Jiaji(EX-B2)，Jianqian，Tianquan(HT2)，Chize(LU5)，Bizhong，Hegu(LI4)，Yinmen(BL37)，Liangqiu(ST34)，Futu(ST32)，Yinlingquan(SP9)，Sanyinjiao(SP6)	11	Yes	4	High dose

Supplemental file 4.Subgroup analyses:Results stratified by acupuncture types

Figure S1. Mean effect sizes of FMA-U – under Electro-acupuncture vs. Fire acupuncture vs. Maunal acupuncture vs. Scalp acupuncture vs. Warm acupuncture

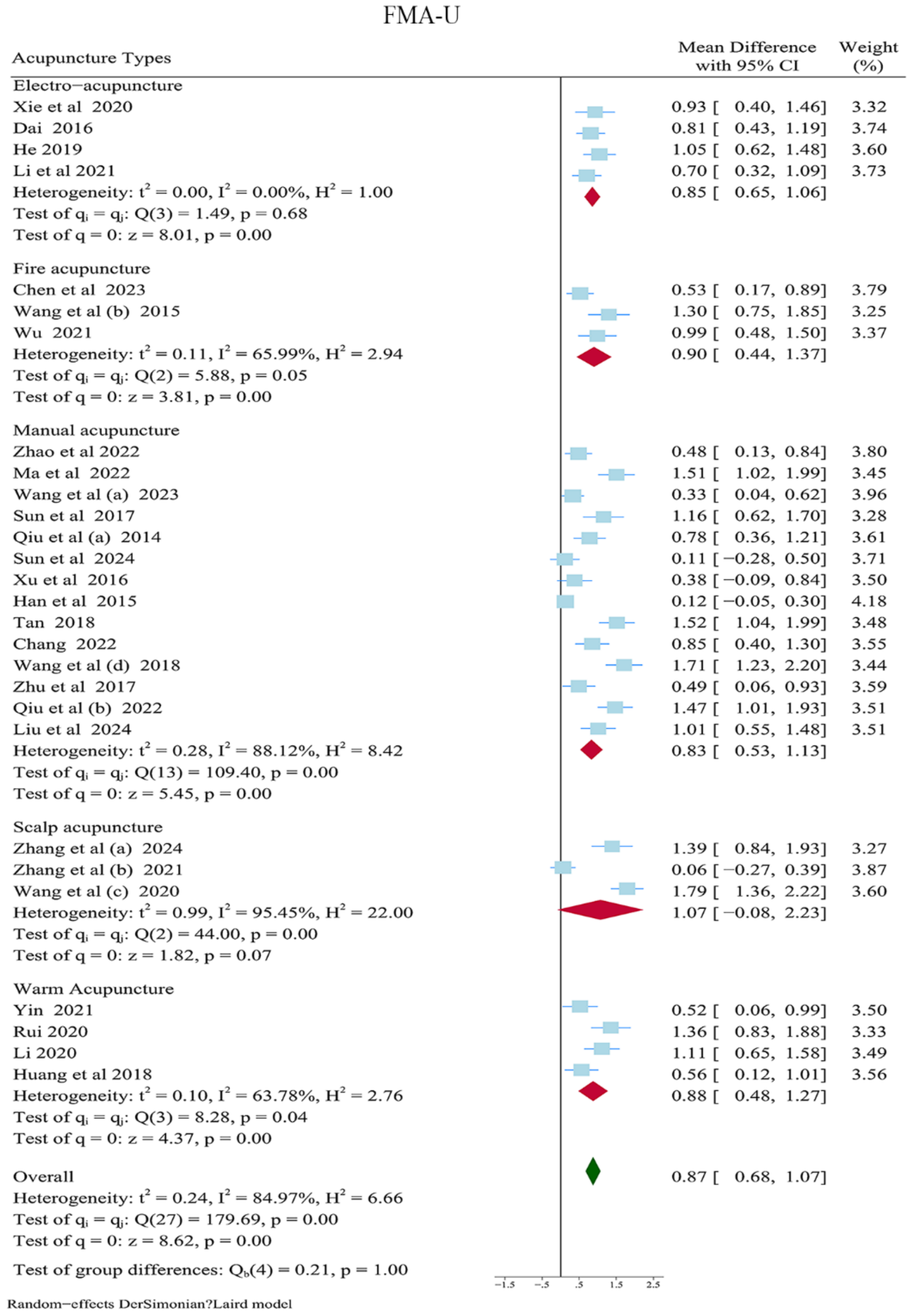

Based on 28 randomized trials. Calculated by using a random-effects model. Between-group difference: P=1.00, I^2=84.97%. CI=confidence interval.

Figure S2. Mean effect sizes of FMA-L – under Electro-acupuncture vs. Fire acupuncture vs. Maunal acupuncture vs. Scalp acupuncture vs. Warm acupuncture

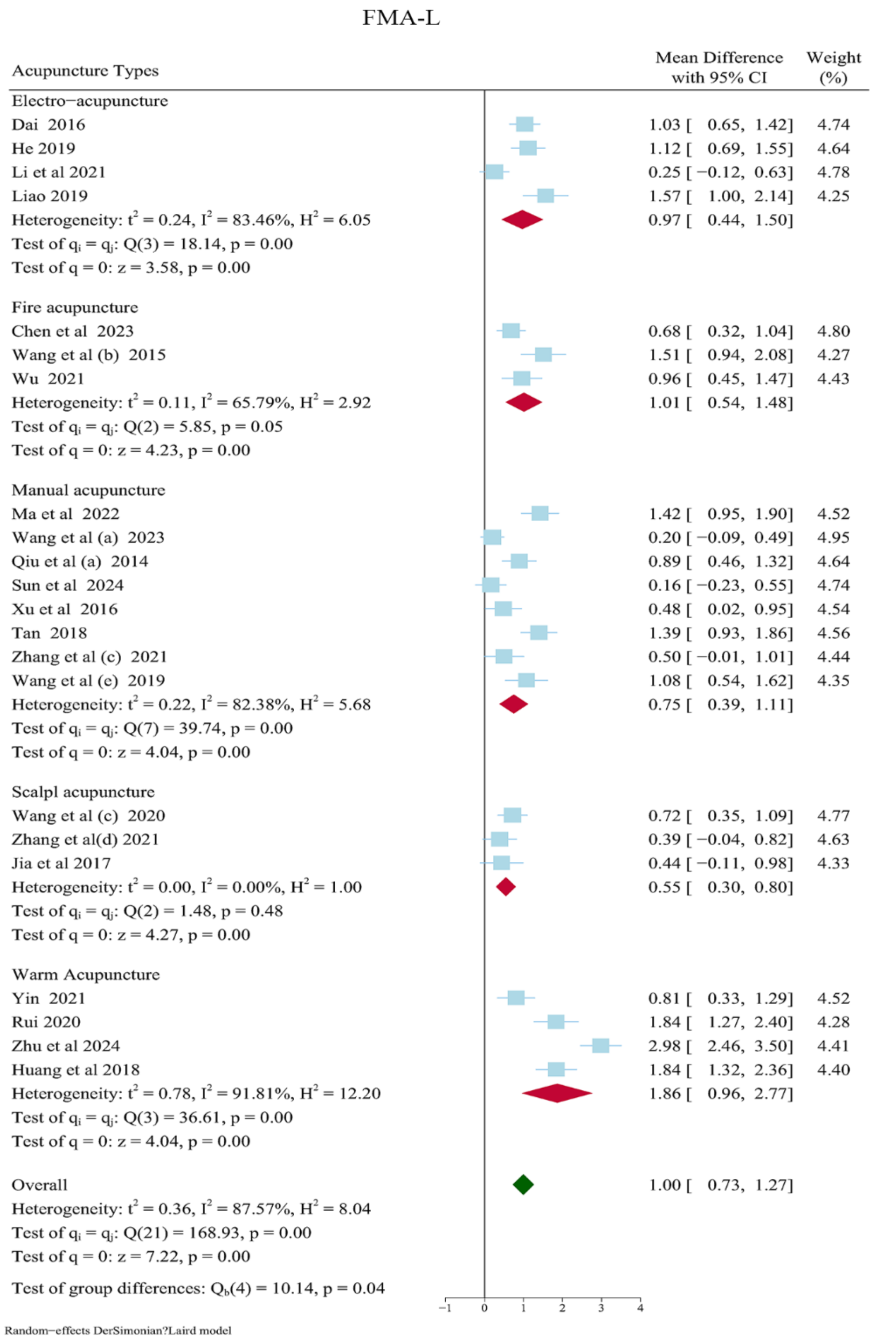

Based on 22 randomized trials. Calculated by using a random-effects model. Between-group difference: *P*=0.04, I^2=87.57%. CI=confidence interval.

Figure S3. Mean effect sizes of MBI – under Electro-acupuncture vs. Fire acupuncture vs. Maunal acupuncture vs. Scalp acupuncture vs. Warm acupuncture

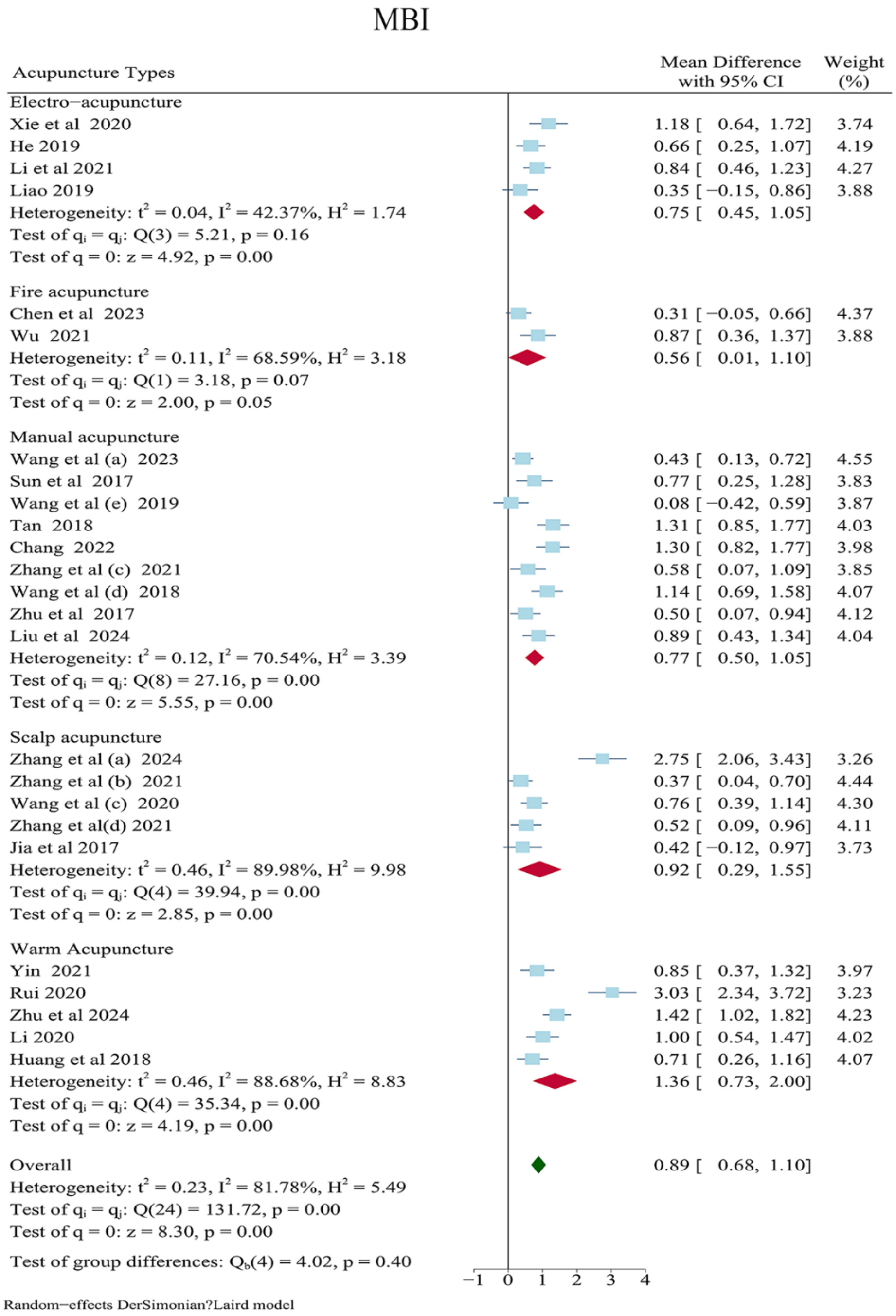

Based on 25 randomized trials. Calculated by using a random-effects model. Between-group difference: *P*=0.40, I^2=81.78%. CI=confidence interval.

Figure S4. Standard mean effect sizes of Spasticity – under Electro-acupuncture vs. Maunal acupuncture vs. Scalp acupuncture vs. Warm acupuncture

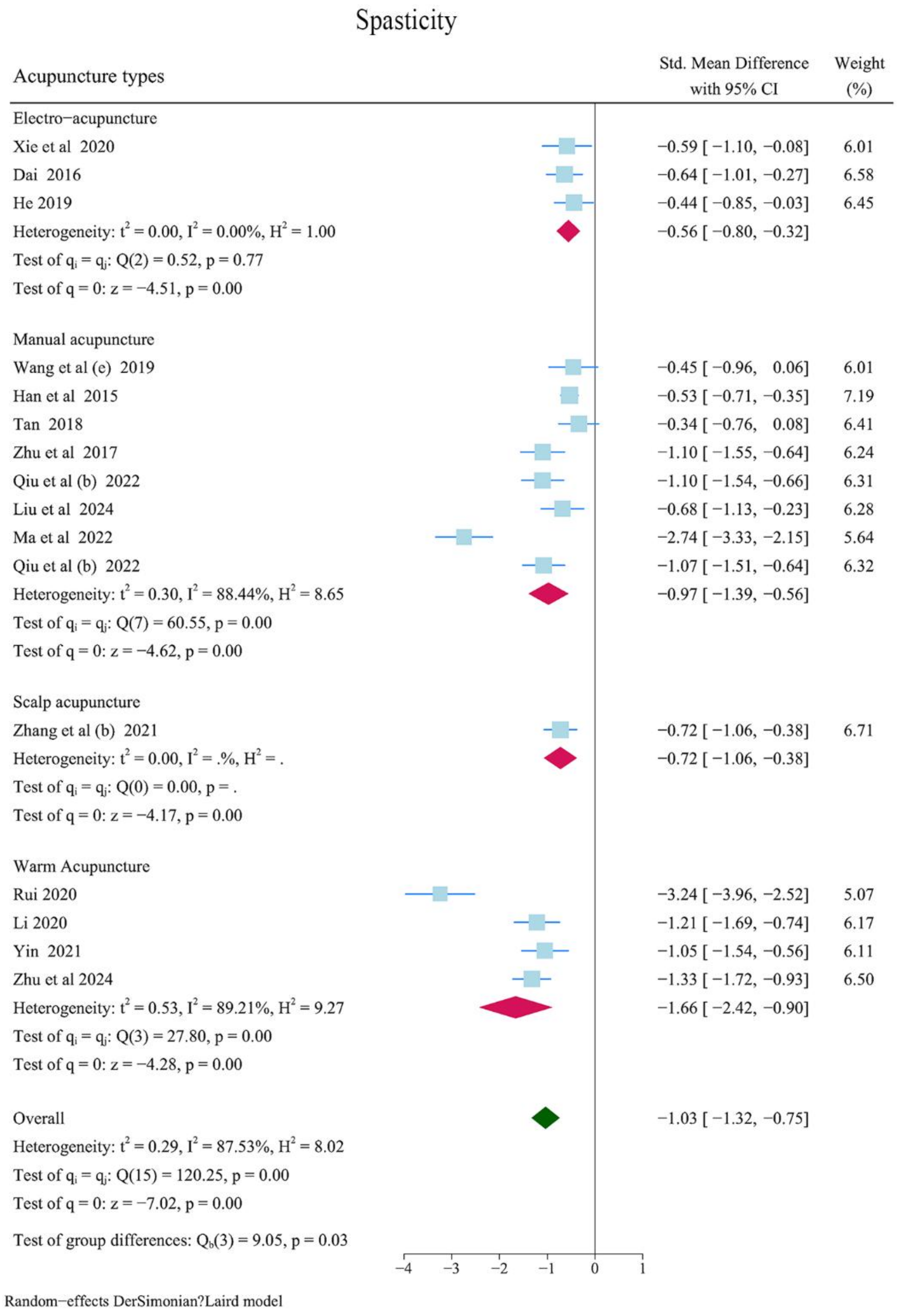

Based on 16 randomized trials. Calculated by using a random-effects model. Between-group difference: P=0.03, I^2=87.53%. CI=confidence interval. Std.=standard

Supplemental file 5.Subgroup analyses:Results stratified by acupuncture dose

Figure S5. Mean effect sizes of FMA-U – under High dose vs. Medium dose vs. Low dose

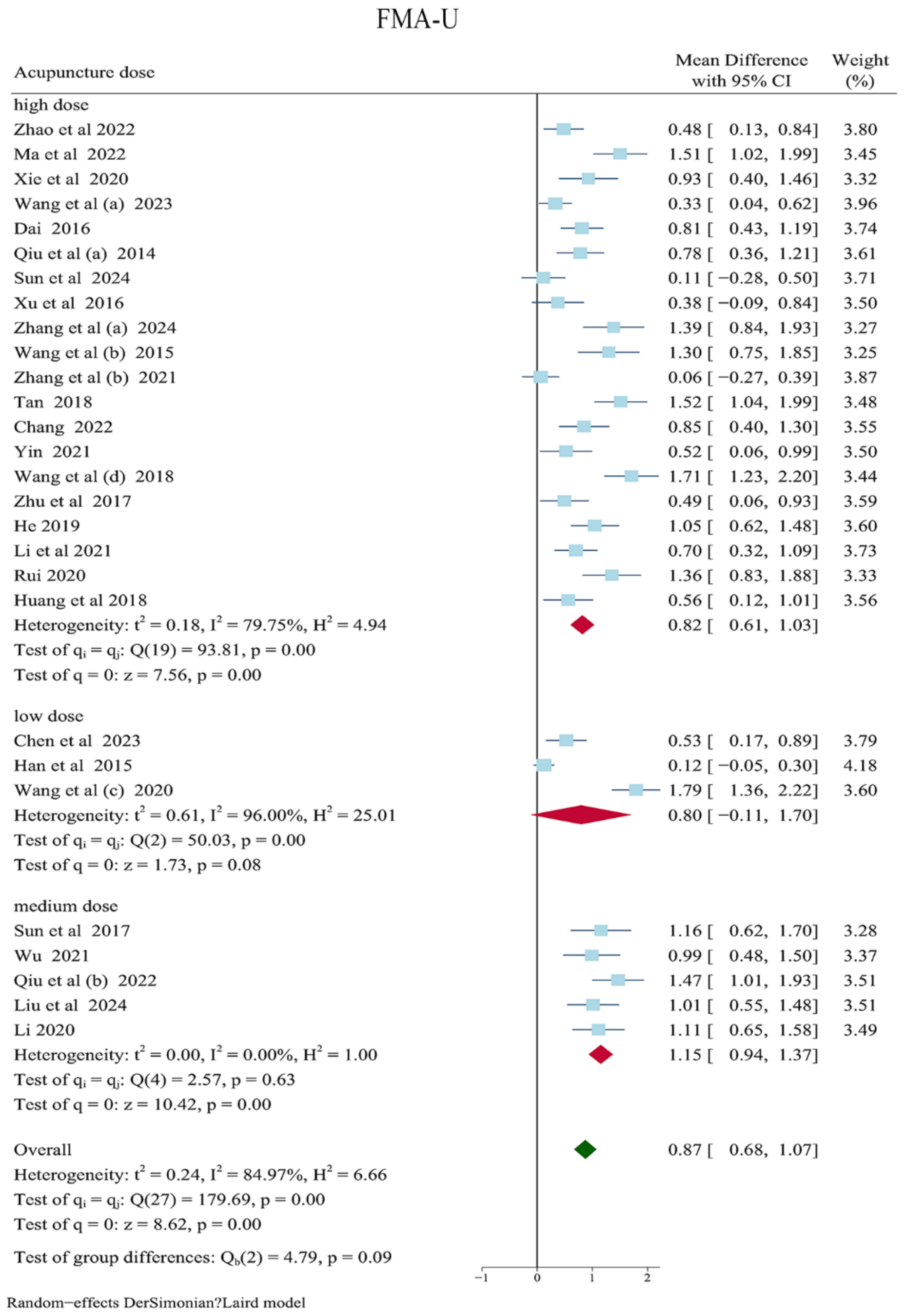

Based on 28 randomized trials. Calculated by using a random-effects model. Between-group difference: *P*=0.09, I^2=84.97%. CI=confidence interval.

Figure S6. Mean effect sizes of FMA-L – under High dose vs. Medium dose vs. Low dose

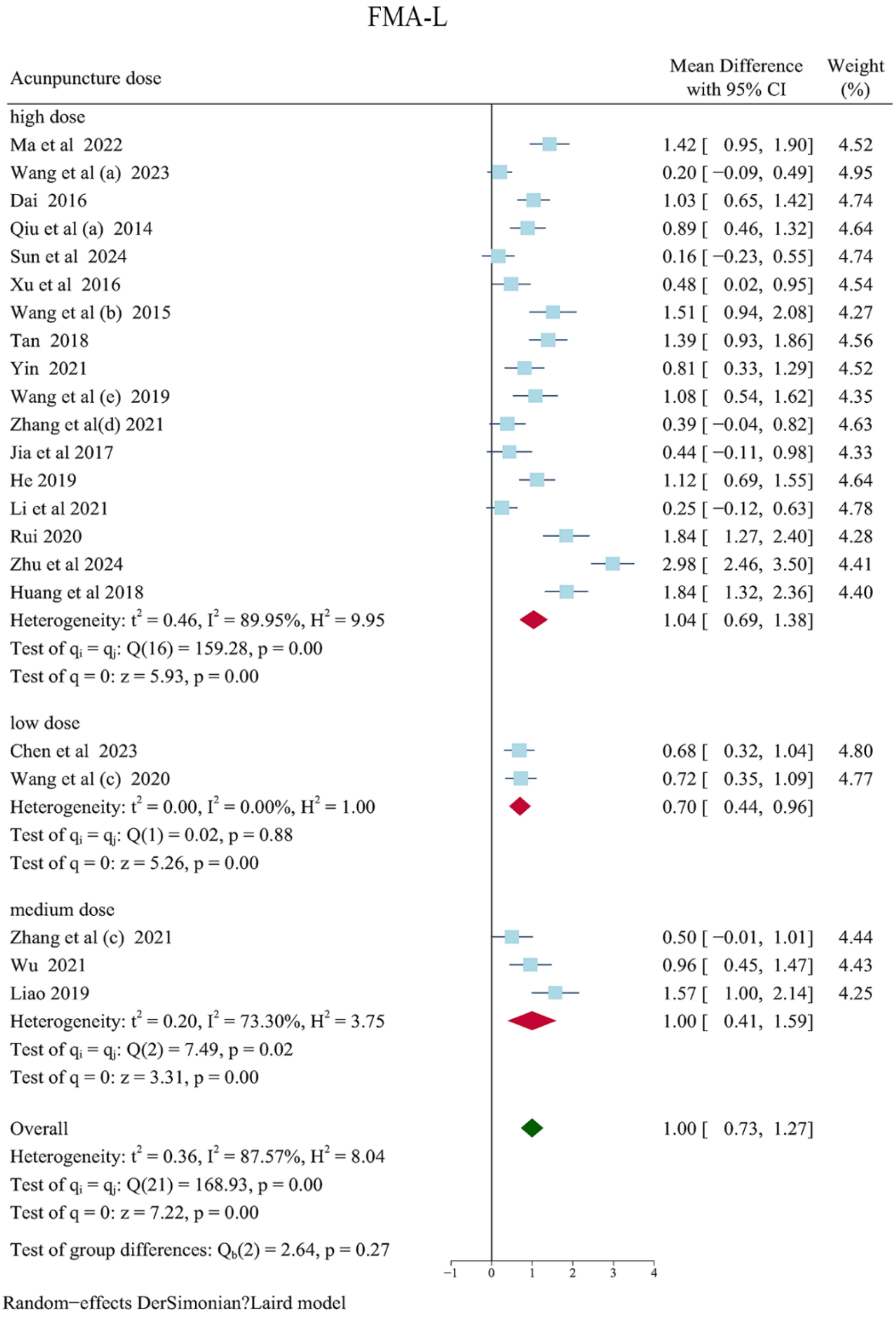

Based on 22 randomized trials. Calculated by using a random-effects model. Between-group difference: *P*=0.27, I^2=87.57%. CI=confidence interval.

Figure S7. Mean effect sizes of MBI – under High dose vs. Medium dose vs. Low dose

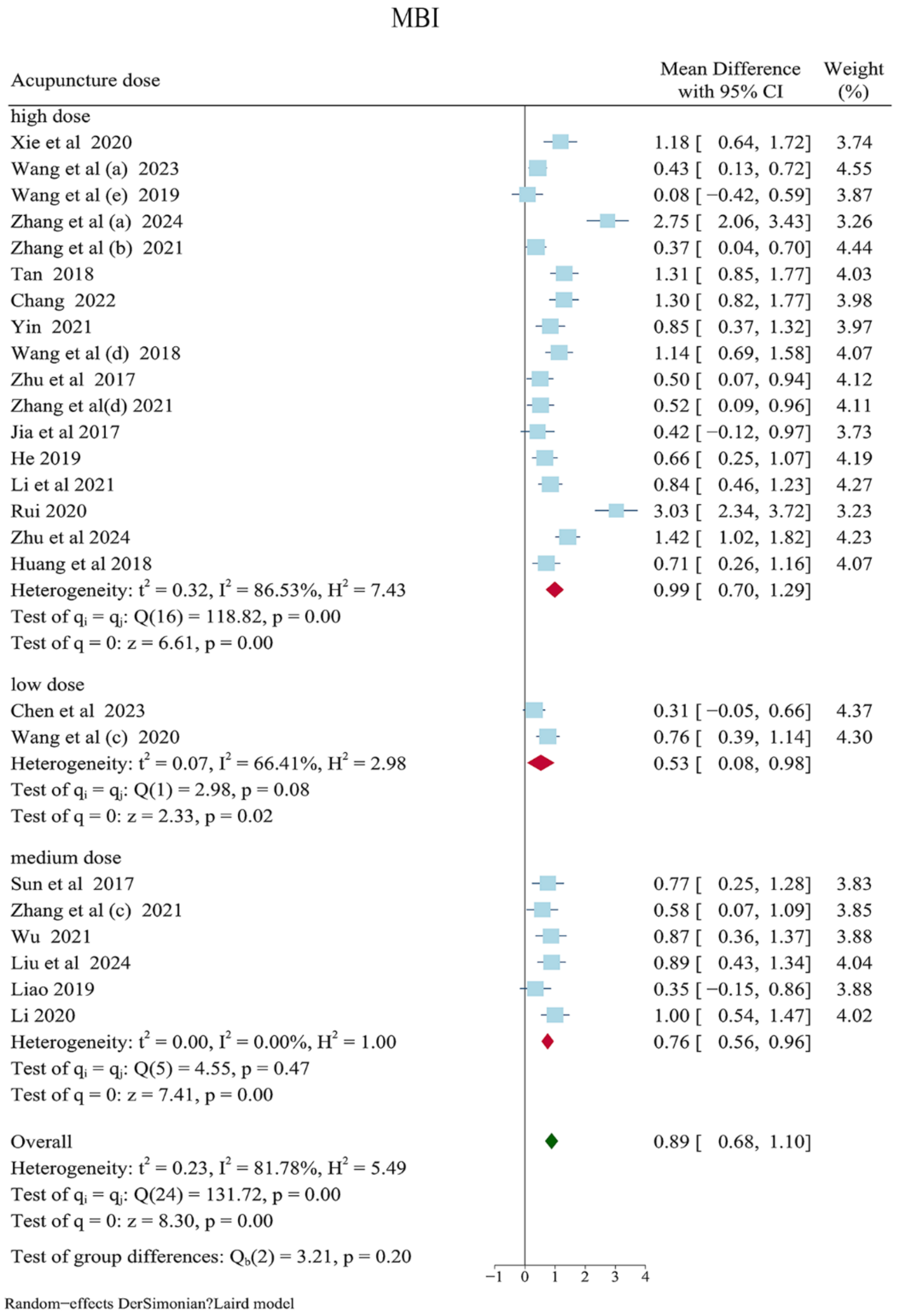

Based on 25 randomized trials. Calculated by using a random-effects model. Between-group difference: *P*=0.20, I^2=81.78%. CI=confidence interval.

Figure S8. Standard mean effect sizes of Spasticity – under High dose vs. Medium dose vs. Low dose

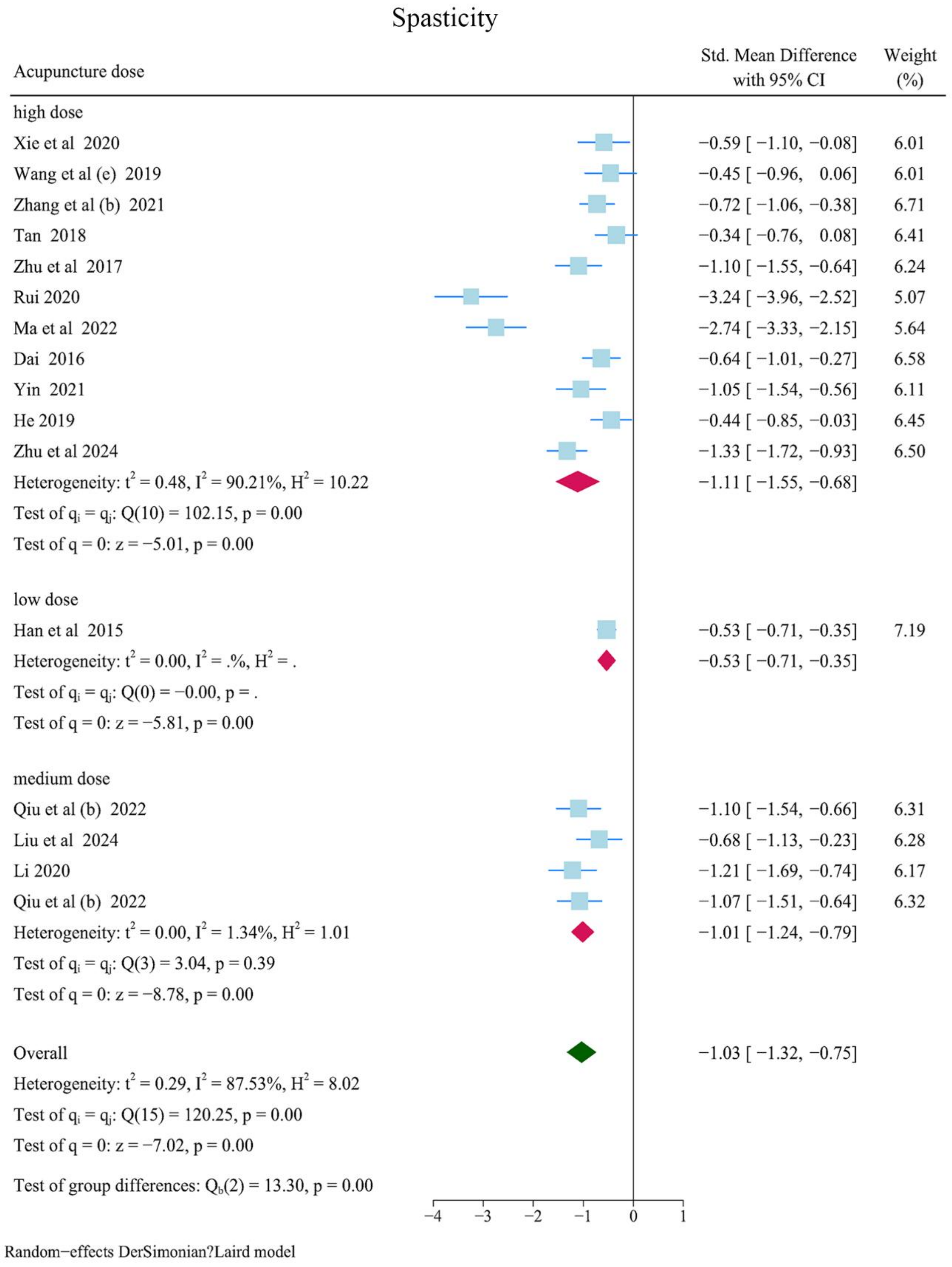

Based on 16 randomized trials. Calculated by using a random-effects model. Between-group difference: P < 0.001, I^2=87.53%. CI=confidence interval. Std.=standard

Supplemental file 6.Subgroup analyses:Results stratified by Outcomes

Figure S9. Standard mean effect sizes of Spasticity – under MAS vs. CSI

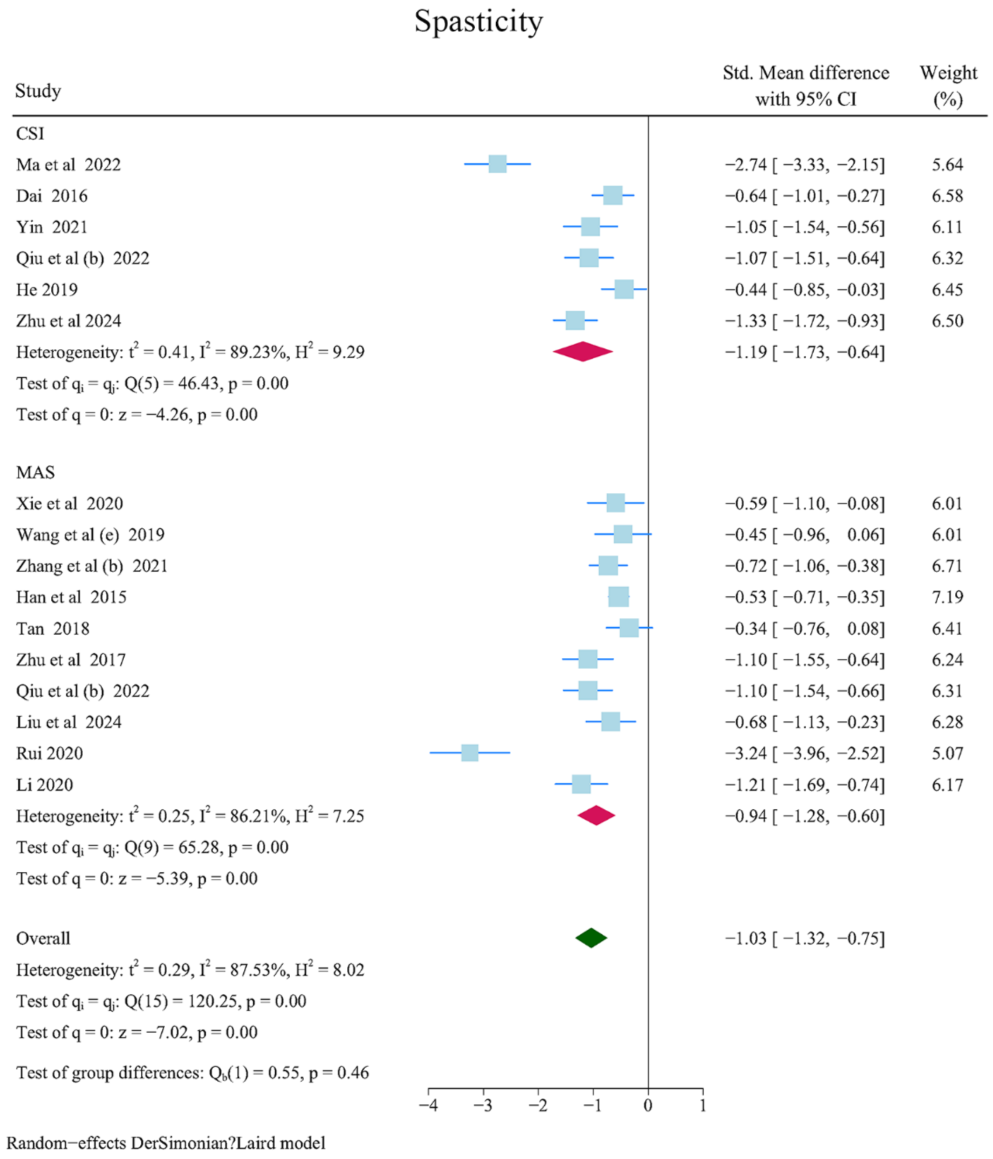

Based on 16 randomized trials. Calculated by using a random-effects model. Between-group difference: *P*=0.46, I^2=87.53%. CI=confidence interval. Std.=standard

Supplemnetal file 7 Subgroup analyses:Results stratified by Stroke subtypes

Figure S10. Standard mean effect sizes of FMA-U – under IS vs. IS+HS

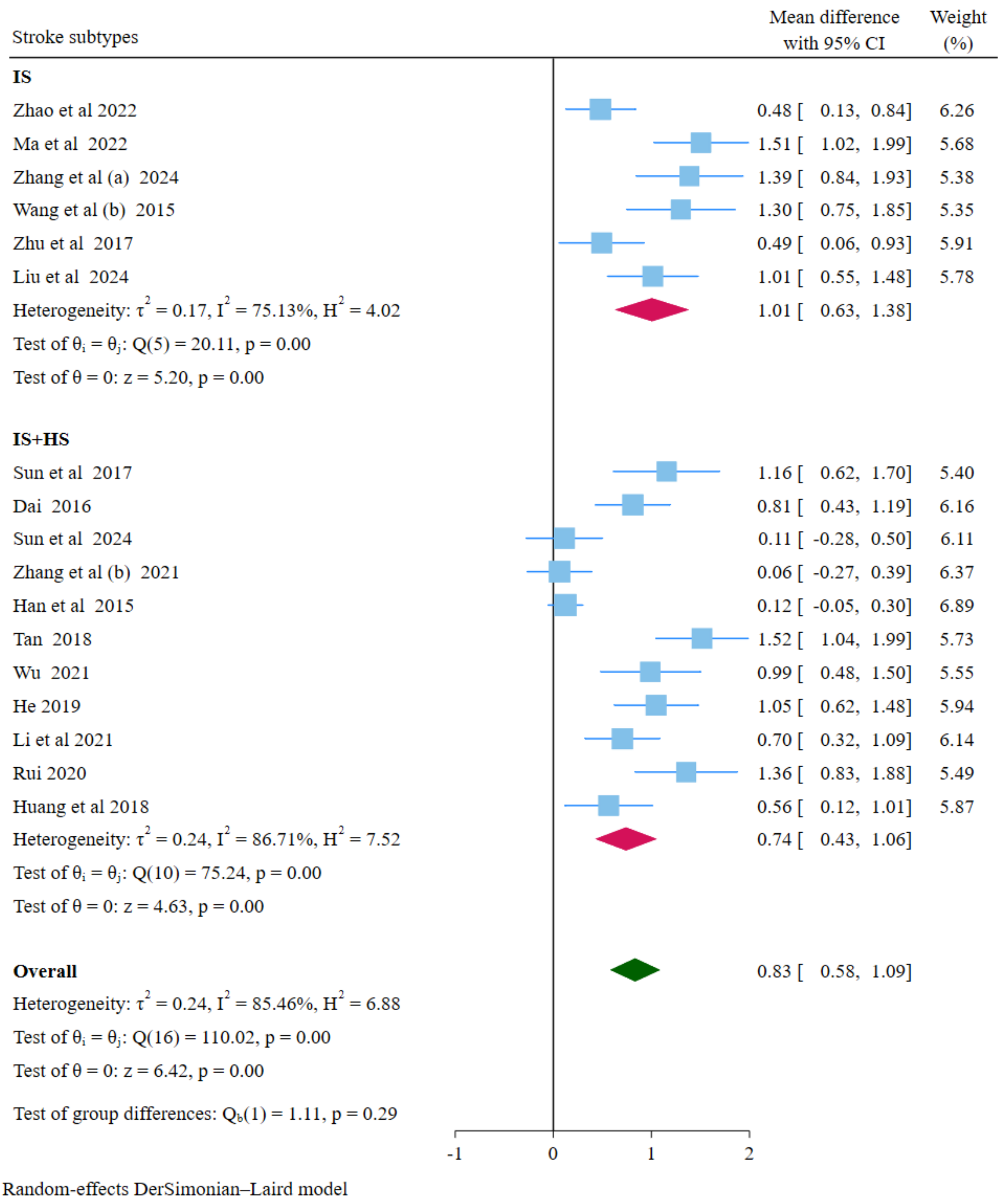

Based on 17 randomized trials. Calculated by using a random-effects model. Between-group difference: $P < 0.001$, I^2=85.46%. CI=confidence interval. Std.=standard

Figure S11. Standard mean effect sizes of FMA-L – under IS vs. IS+HS

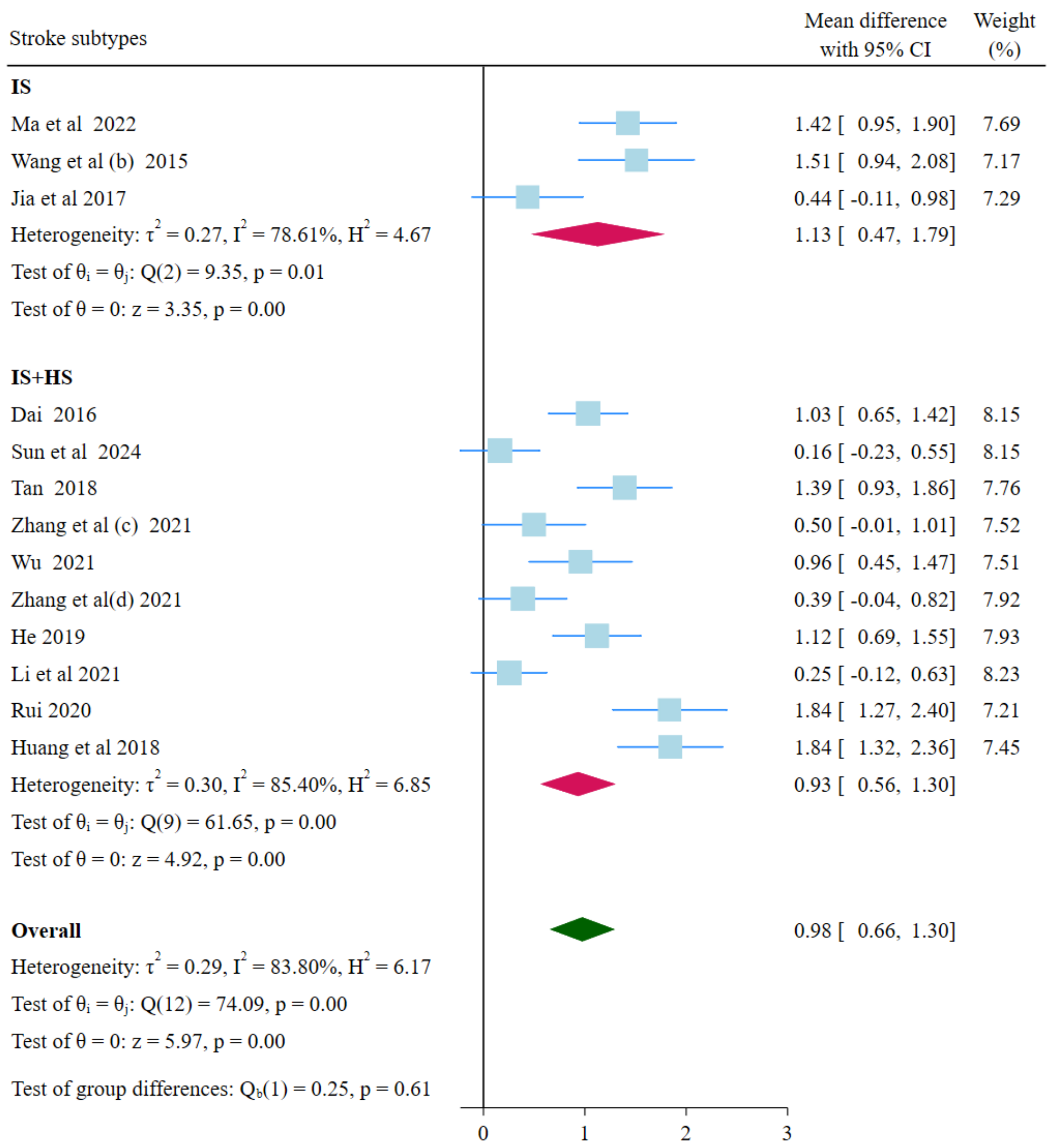

Random-effects DerSimonian–Laird model

Based on 13 randomized trials. Calculated by using a random-effects model. Between-group difference: $P < 0.001$, I^2=83.80%. CI=confidence interval. Std.=standard

Figure S12. Standard mean effect sizes of MBI – under IS vs. IS+HS

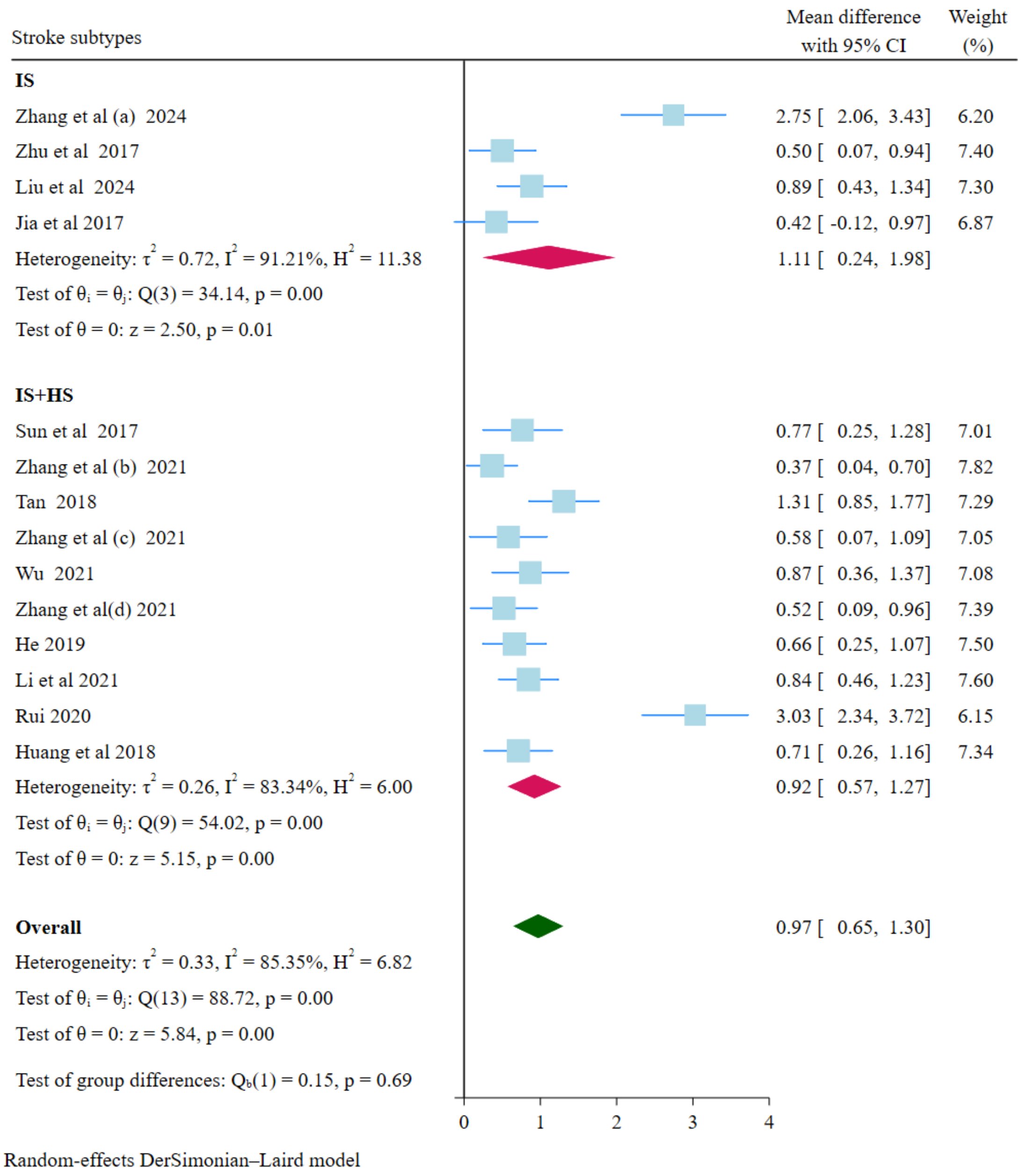

Based on 14 randomized trials. Calculated by using a random-effects model. Between-group difference: $P < 0.001$, I^2=85.35%. CI=confidence interval. Std.=standard

Figure S13. Standard mean effect sizes of Spasticity – under IS vs. IS+HS

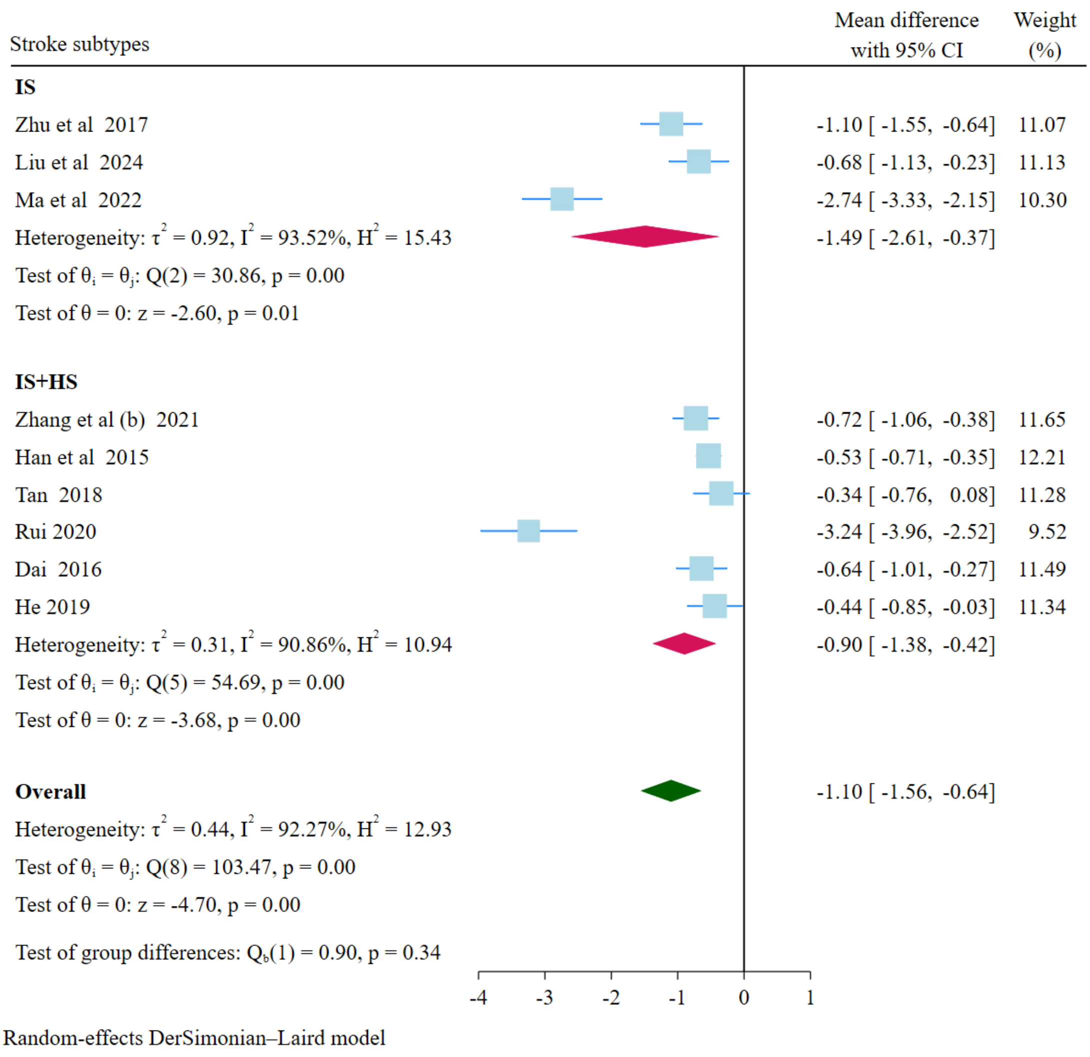

Based on 9 randomized trials. Calculated by using a random-effects model. Between-group difference: $P < 0.001$, I^2=92.27%. CI=confidence interval. Std.=standard

Supplemnetal file 8 Subgroup analyses:Results stratified by disease course

Figure S14. Standard mean effect sizes of FMA-U – 30~90 vs. ＞90 vs. ＜30

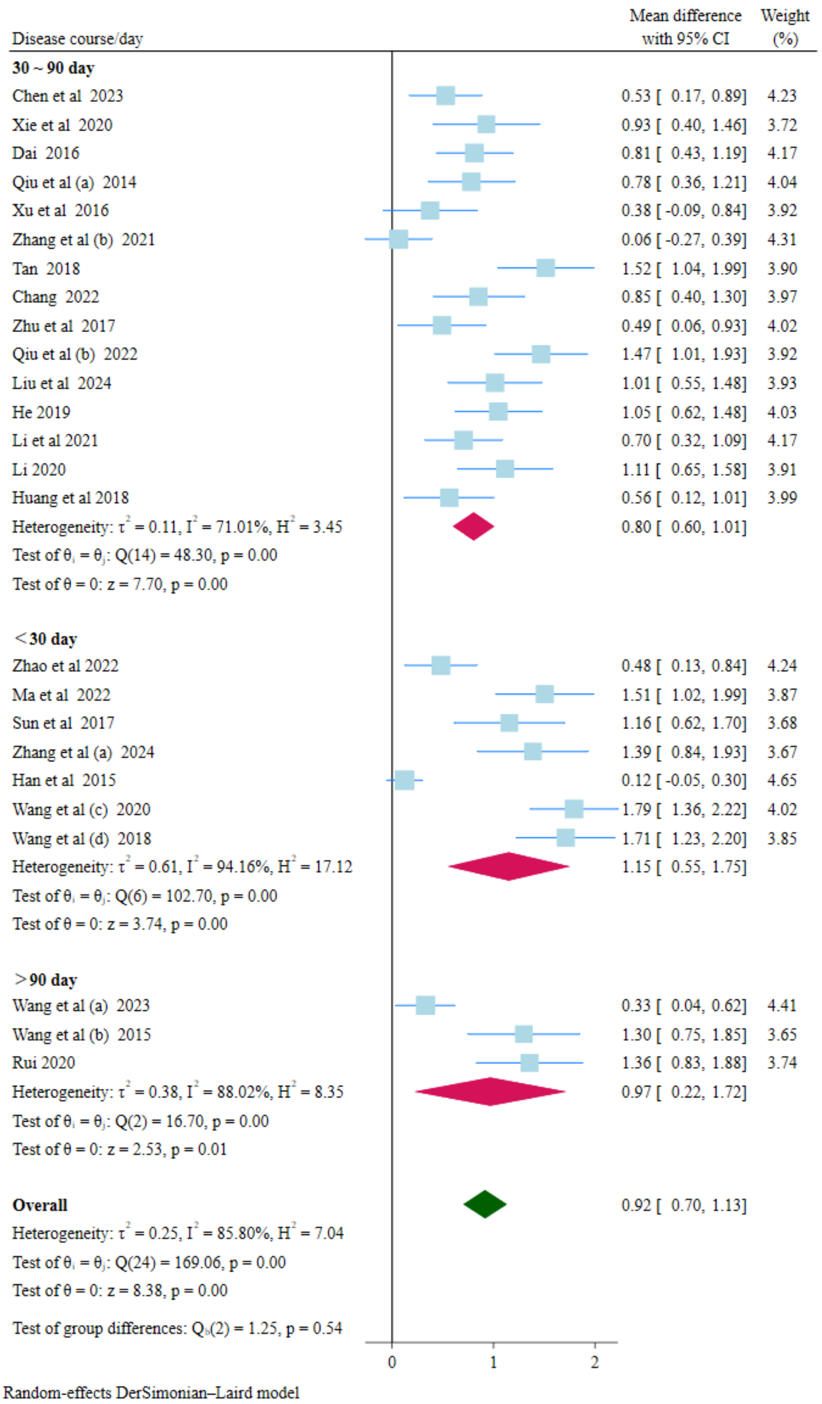

Based on 25 randomized trials. Calculated by using a random-effects model. Between-group difference: $P < 0.001$, I^2=85.80%. CI=confidence interval. Std.=standard

frontiers

Figure S15. Standard mean effect sizes of FMA-L – 30~90 vs. ＞90 vs. ＜30

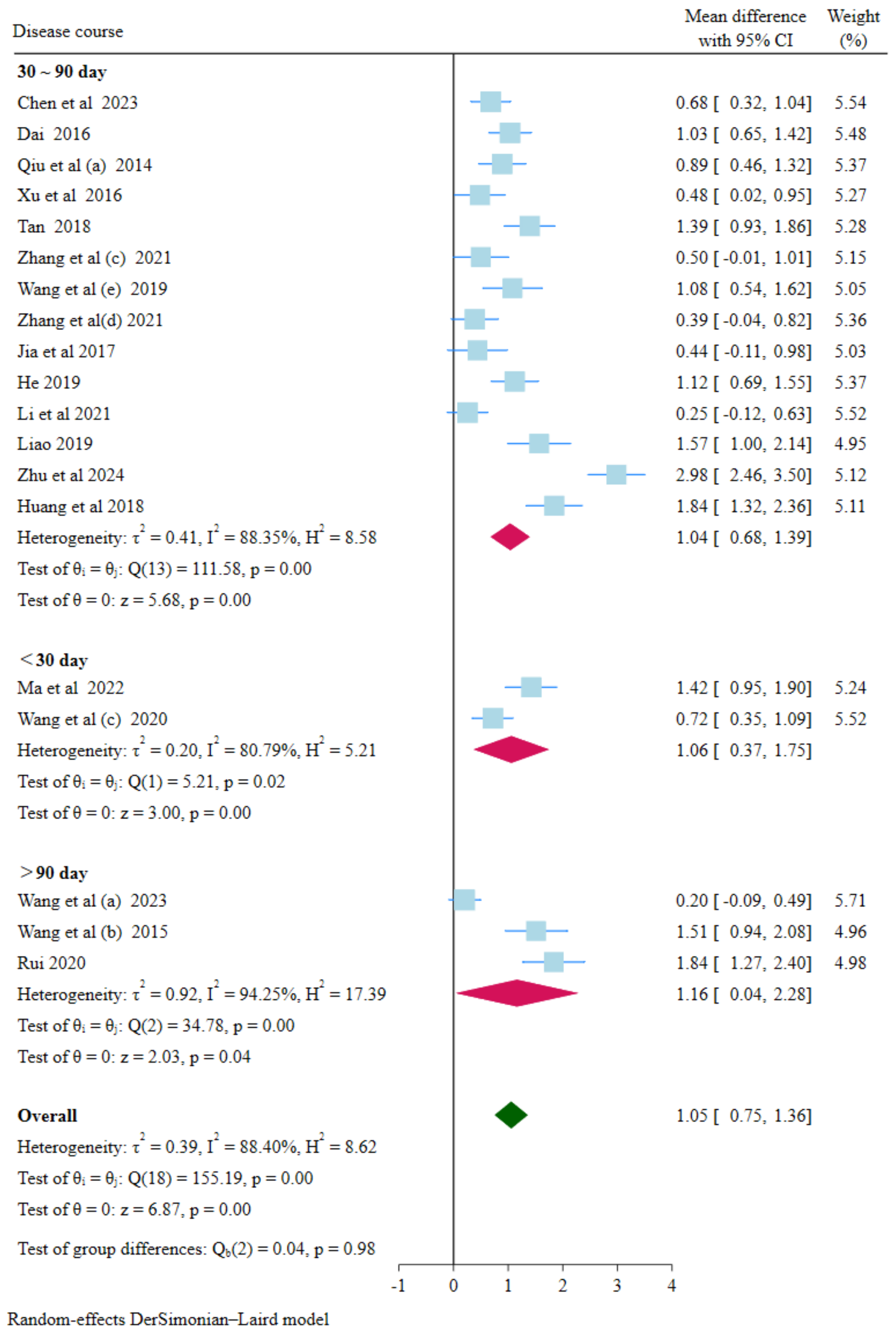

Based on 19 randomized trials. Calculated by using a random-effects model. Between-group difference: $P < 0.001$, I^2=88.40%. CI=confidence interval. Std.=standard

Figure S16. Standard mean effect sizes of MBI – 30~90 vs. ＞90 vs. ＜30

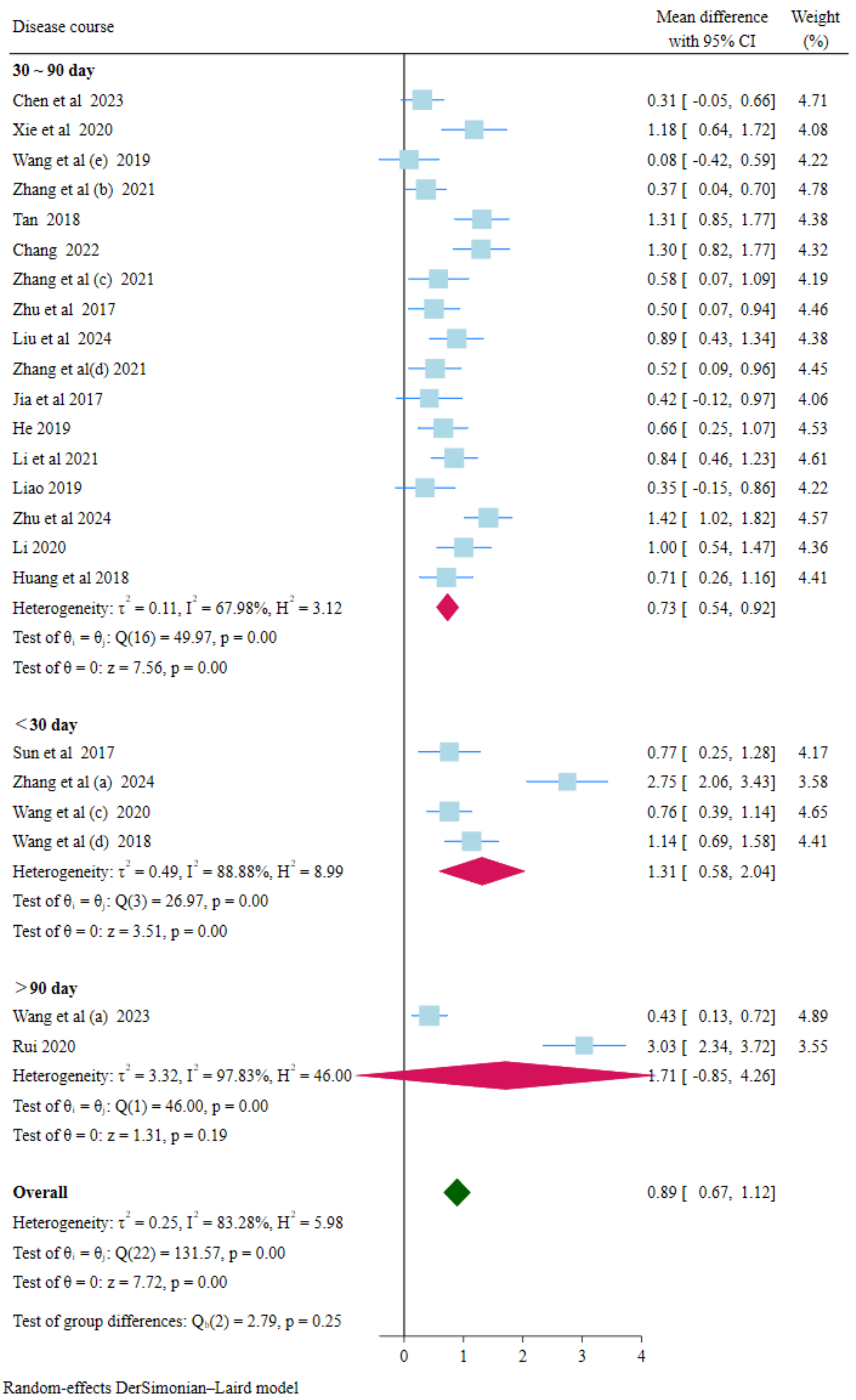

Based on 23 randomized trials. Calculated by using a random-effects model. Between-group difference: $P < 0.001$, I^2=88.40%. CI=confidence interval. Std.=standard

Figure S17. Standard mean effect sizes of Spasticity – 30~90 vs. ＞90 vs. ＜30

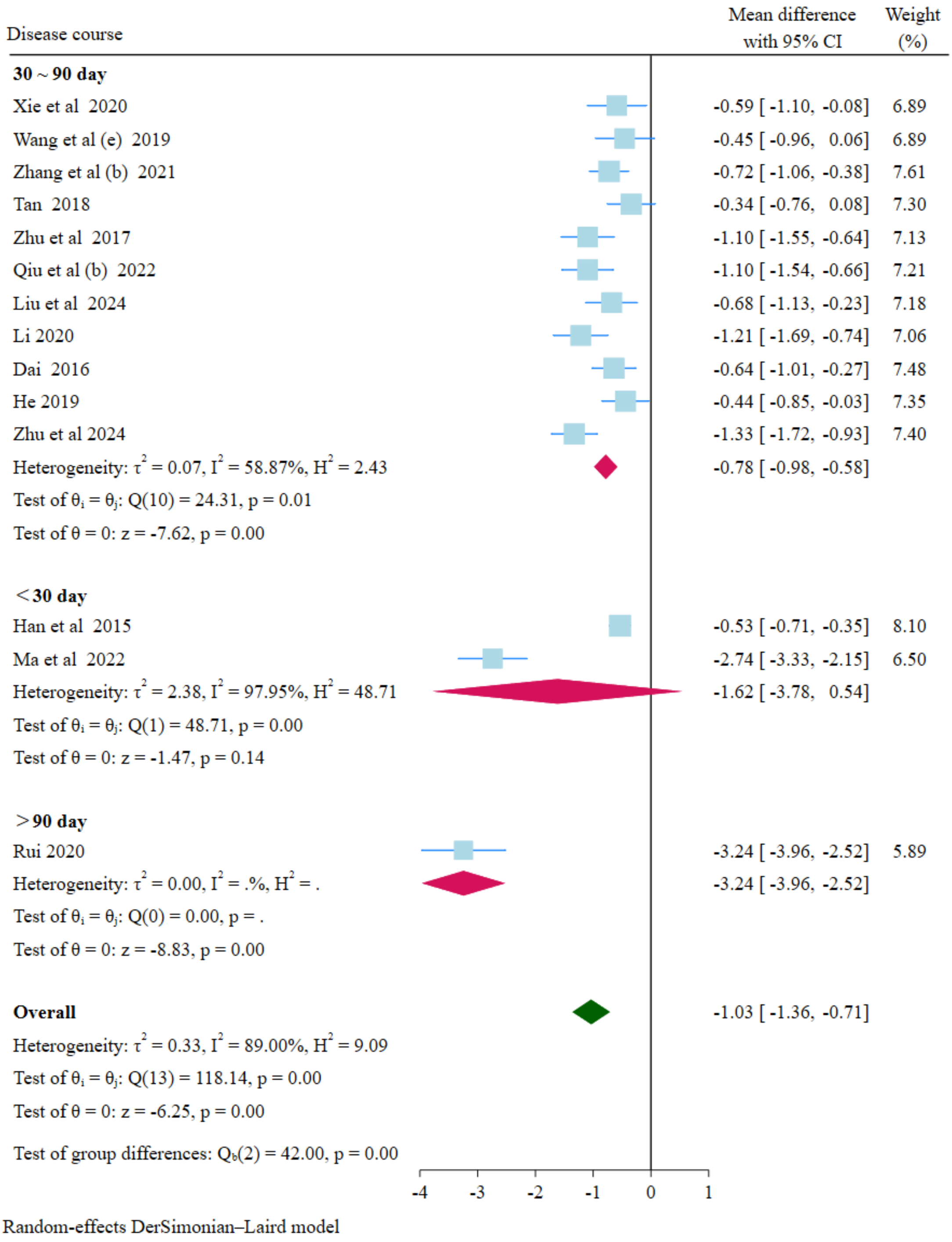

Based on 14 randomized trials. Calculated by using a random-effects model. Between-group difference: $P < 0.001$, I^2=89.00%. CI=confidence interval. Std.=standard

Supplemnetal file 9 Subgroup analyses:Results stratified by mean age

Figure S17. Standard mean effect sizes of FMA-U –>60 vs. <60

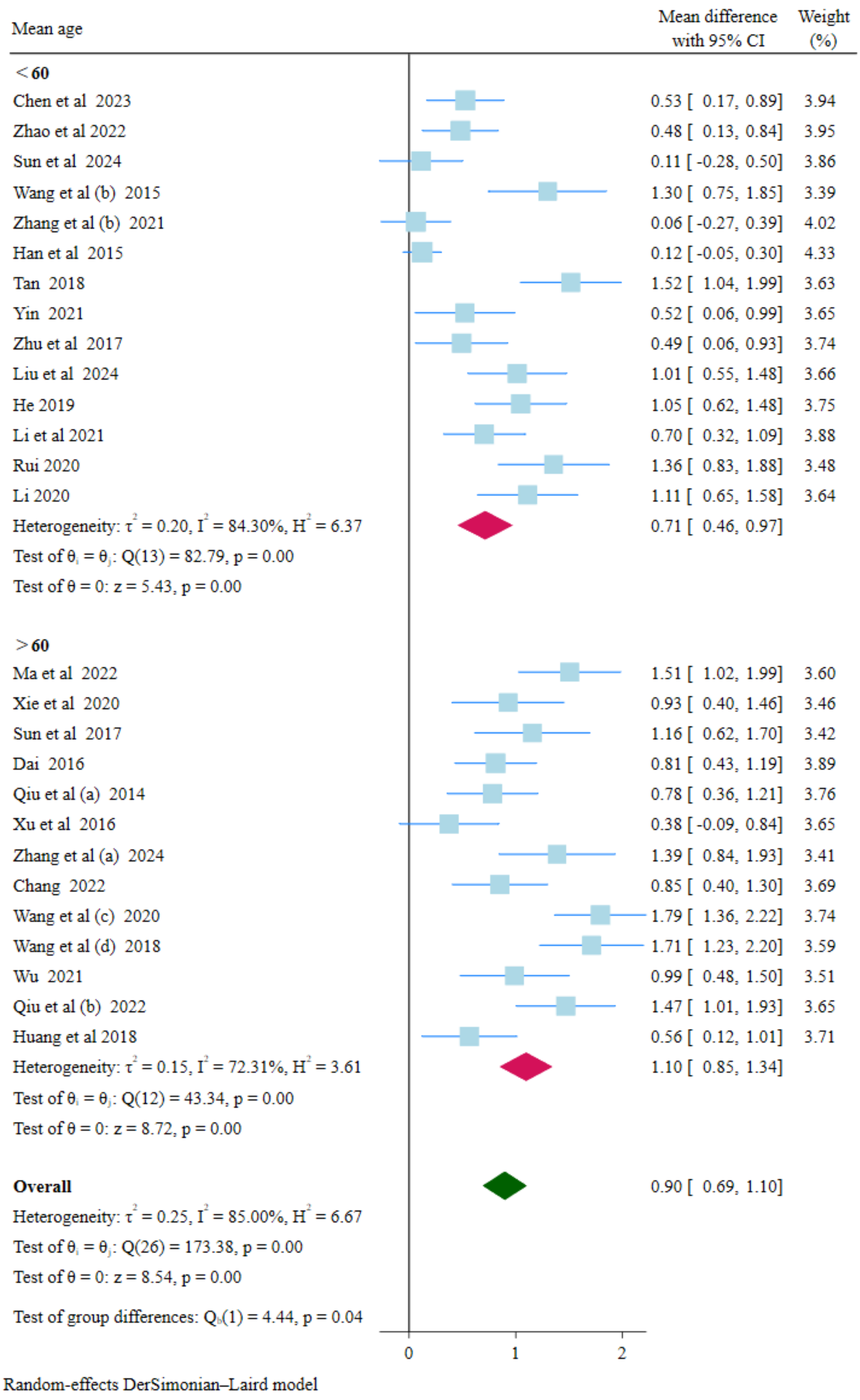

Based on 27 randomized trials. Calculated by using a random-effects model. Between-group difference: $P < 0.001$, I^2=85.00%. CI=confidence interval. Std.=standard

Figure S18. Standard mean effect sizes of FMA-L –＞60 vs. ＜60

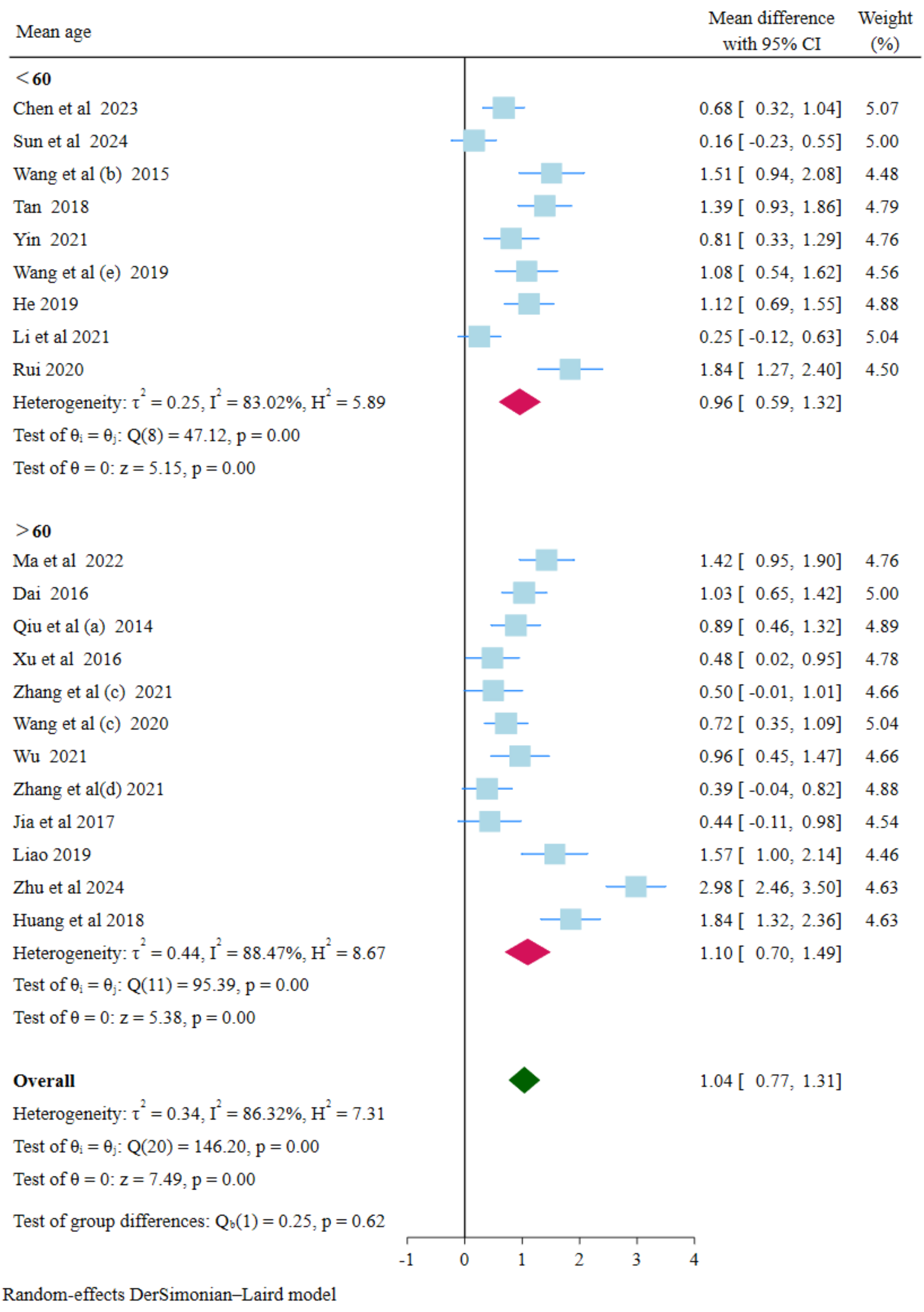

Based on 21 randomized trials. Calculated by using a random-effects model. Between-group difference: $P < 0.001$, I^2=86.32%. CI=confidence interval. Std.=standard

frontiers

Figure S19. Standard mean effect sizes of MBI –>60 vs. ＜60

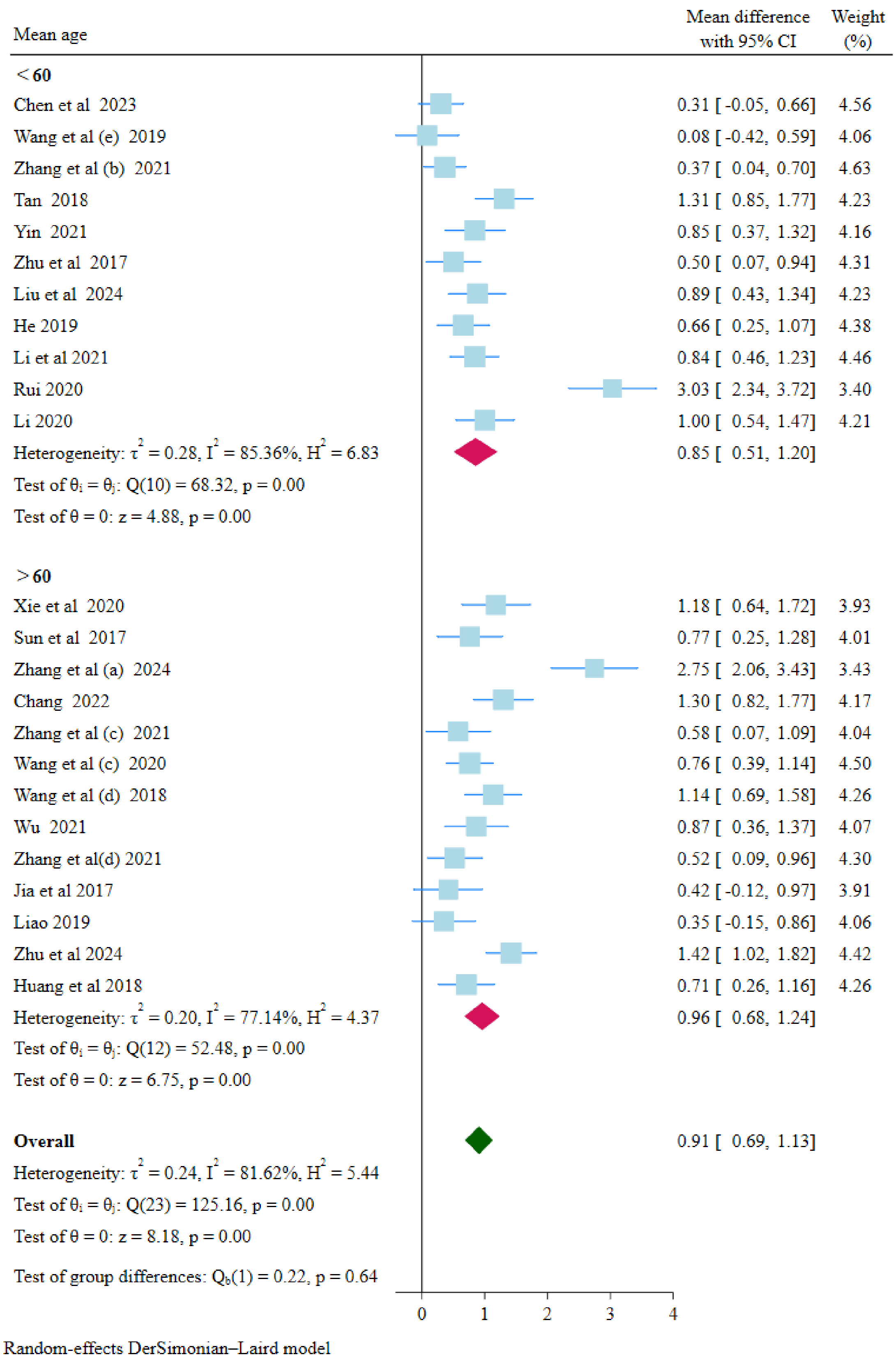

Based on 24 randomized trials. Calculated by using a random-effects model. Between-group difference: $P < 0.001$, I^2=81.62%. CI=confidence interval. Std.=standard

Figure S20. Standard mean effect sizes of Spasticity –＞60 vs. ＜60

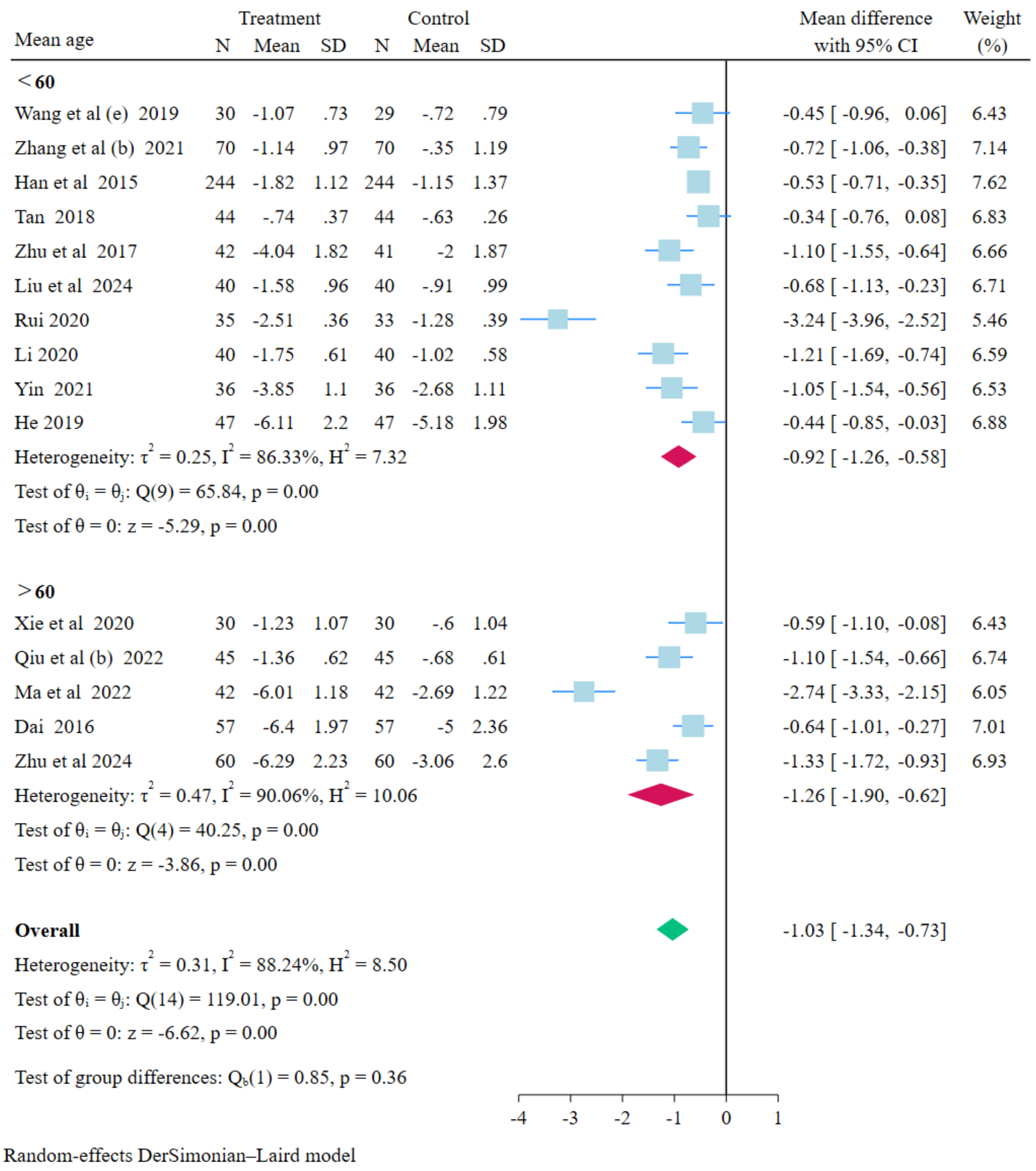

Based on 15 randomized trials. Calculated by using a random-effects model. Between-group difference: $P < 0.001$, I^2=88.24%. CI=confidence interval. Std.=standard

Supplemental file 10. Subgroup analyses:Results stratified by frequency (time/week)

Figure S21 Standard mean effect sizes of FMA-U with 3-5 times/week vs. 6-7 times/week

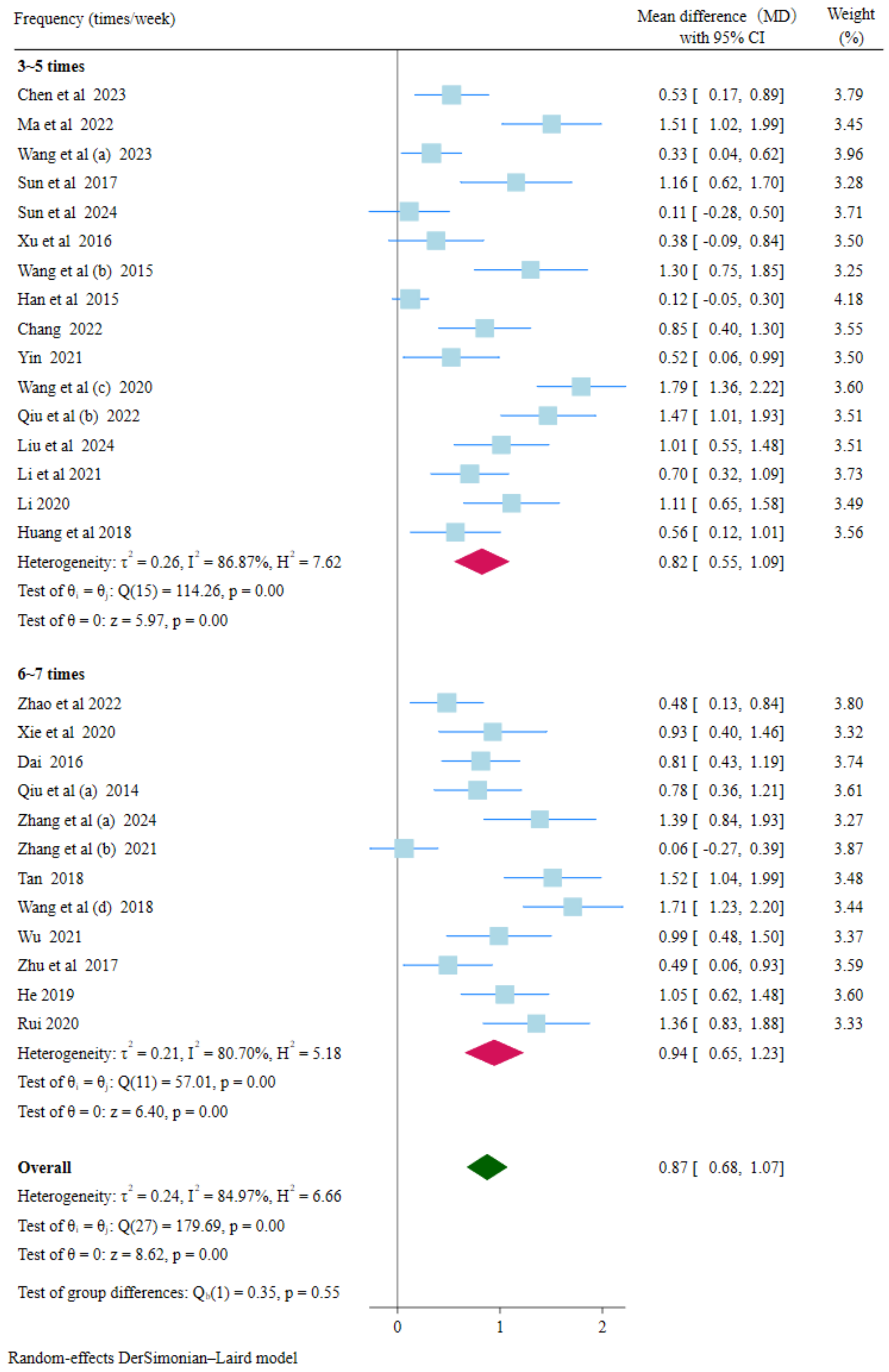

Based on 28 randomized trials. Calculated by using a random-effects model. Between-group difference: *P* < 0.001, I^2=84.97%. CI=confidence interval. Std.=standard

Figure S22 Standard mean effect sizes of FMA-L with 3-5 times/week vs. 6-7 times/week

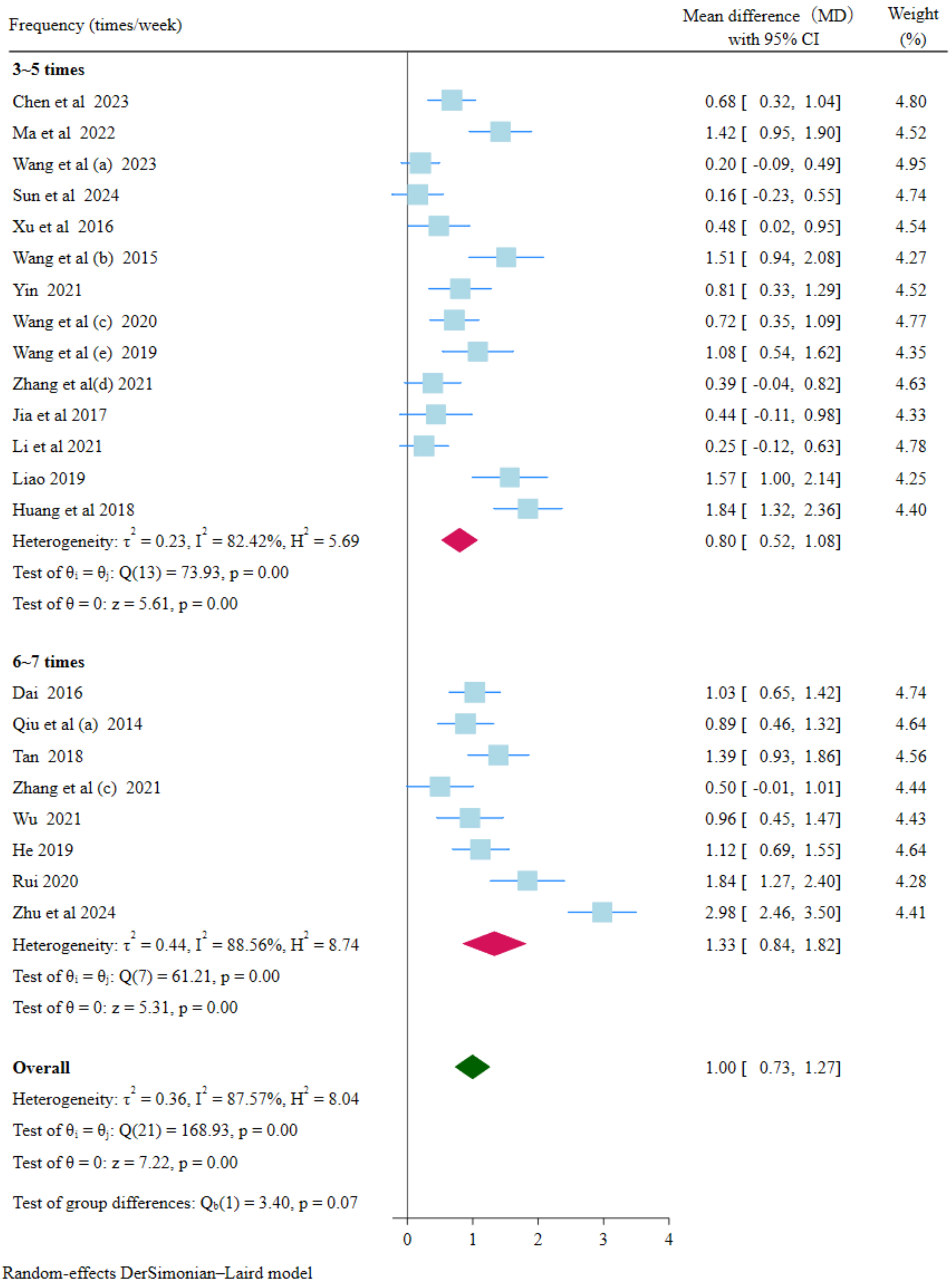

Based on 22 randomized trials. Calculated by using a random-effects model. Between-group difference: $P < 0.001$, I^2=87.57%. CI=confidence interval. Std.=standard

Figure S22 Standard mean effect sizes of MBI with 3-5 times/week vs. 6-7 times/week

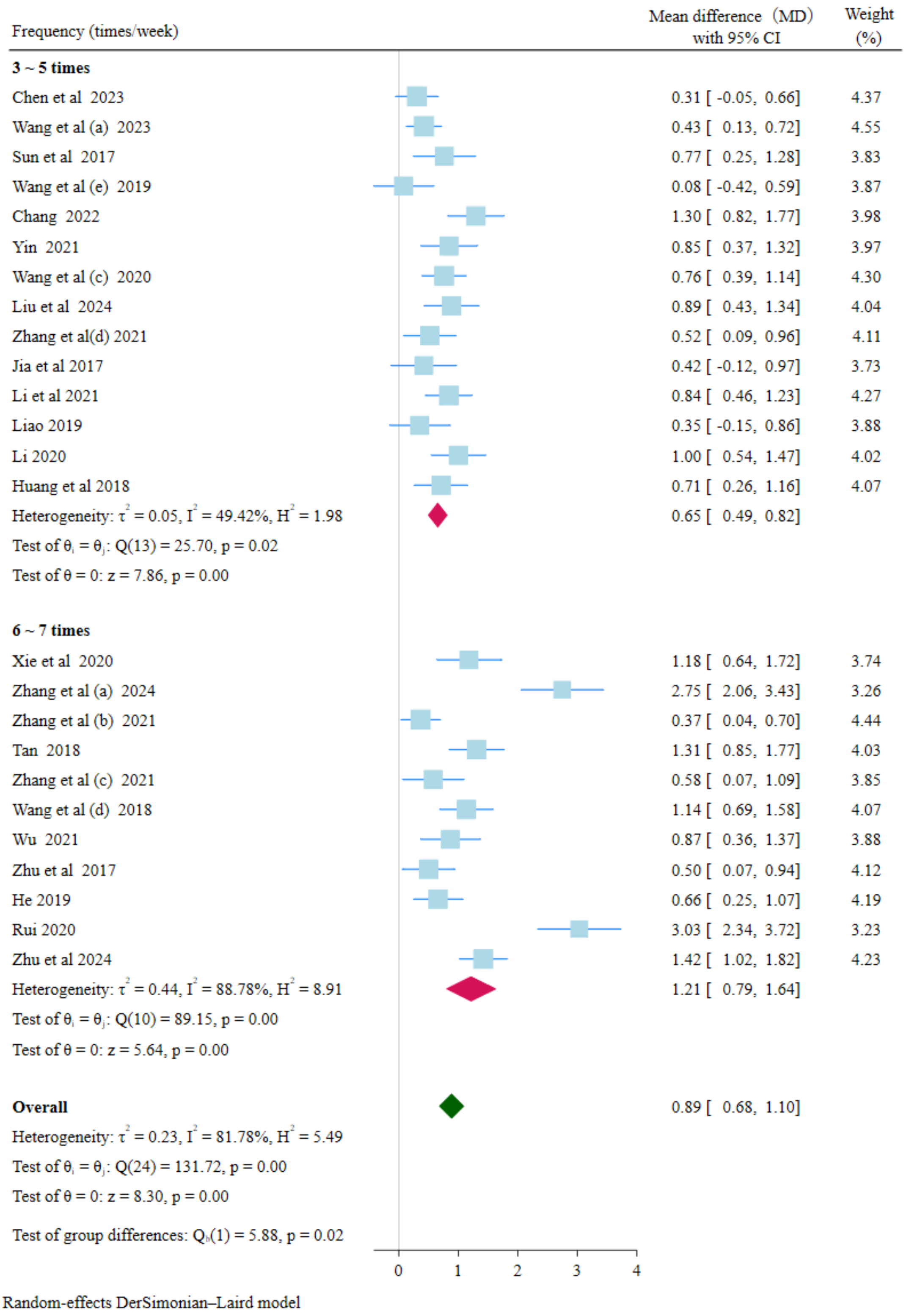

Based on 25 randomized trials. Calculated by using a random-effects model. Between-group difference: $P < 0.001$, I^2=81.78%. CI=confidence interval. Std.=standard

Figure S23 Standard mean effect sizes of Spasticity with 3-5 times/week vs. 6-7 times/week

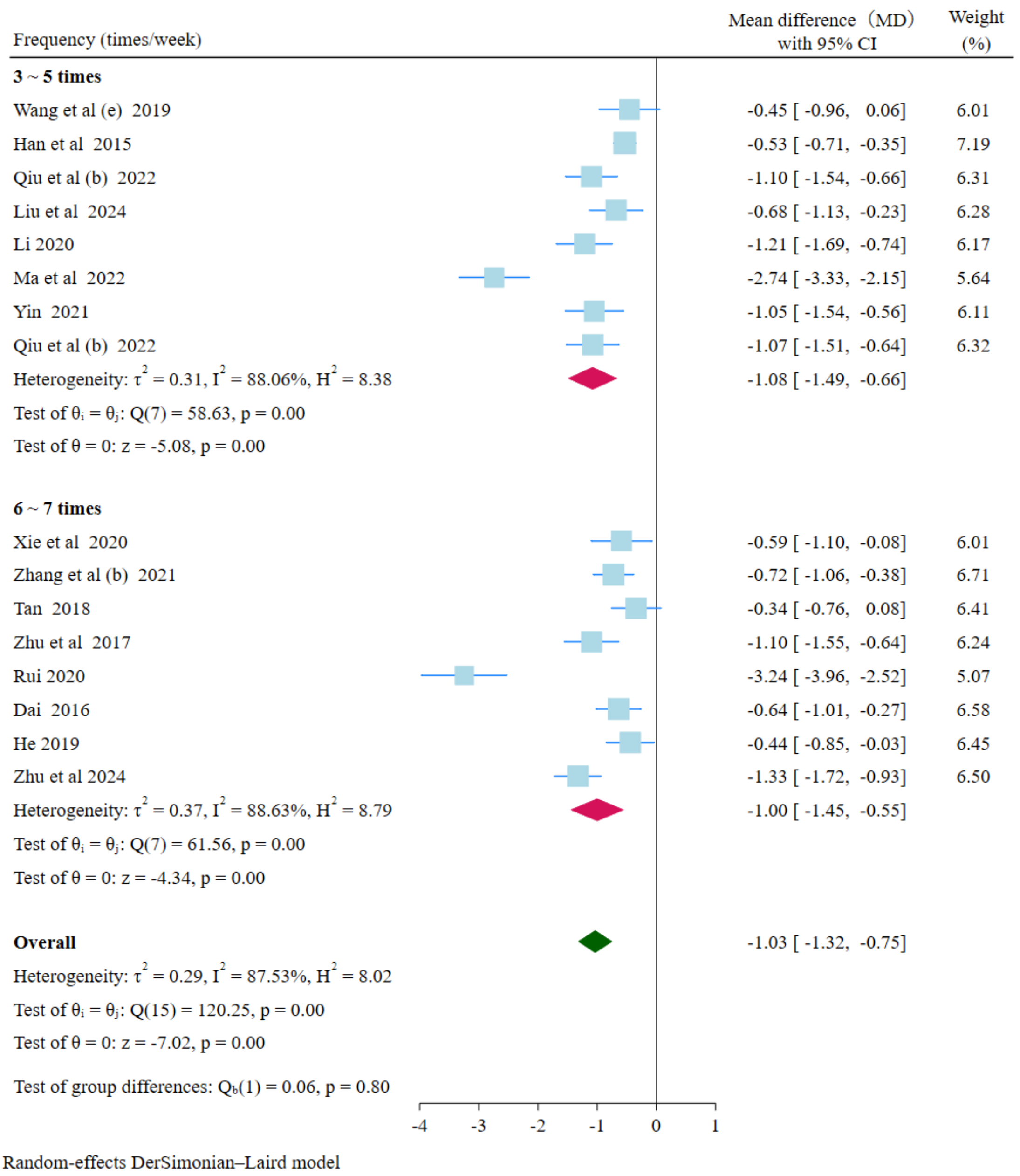

Based on 16 randomized trials. Calculated by using a random-effects model. Between-group difference: $P < 0.001$, I^2=87.53%. CI=confidence interval. Std.=standard

Supplemental file 11. Subgroup analyses:Results stratified by course day (day)

Figure S24 Standard mean effect sizes of FMA-U with 20≤day<30 vs. 30≤day<40 vs. 40≤day

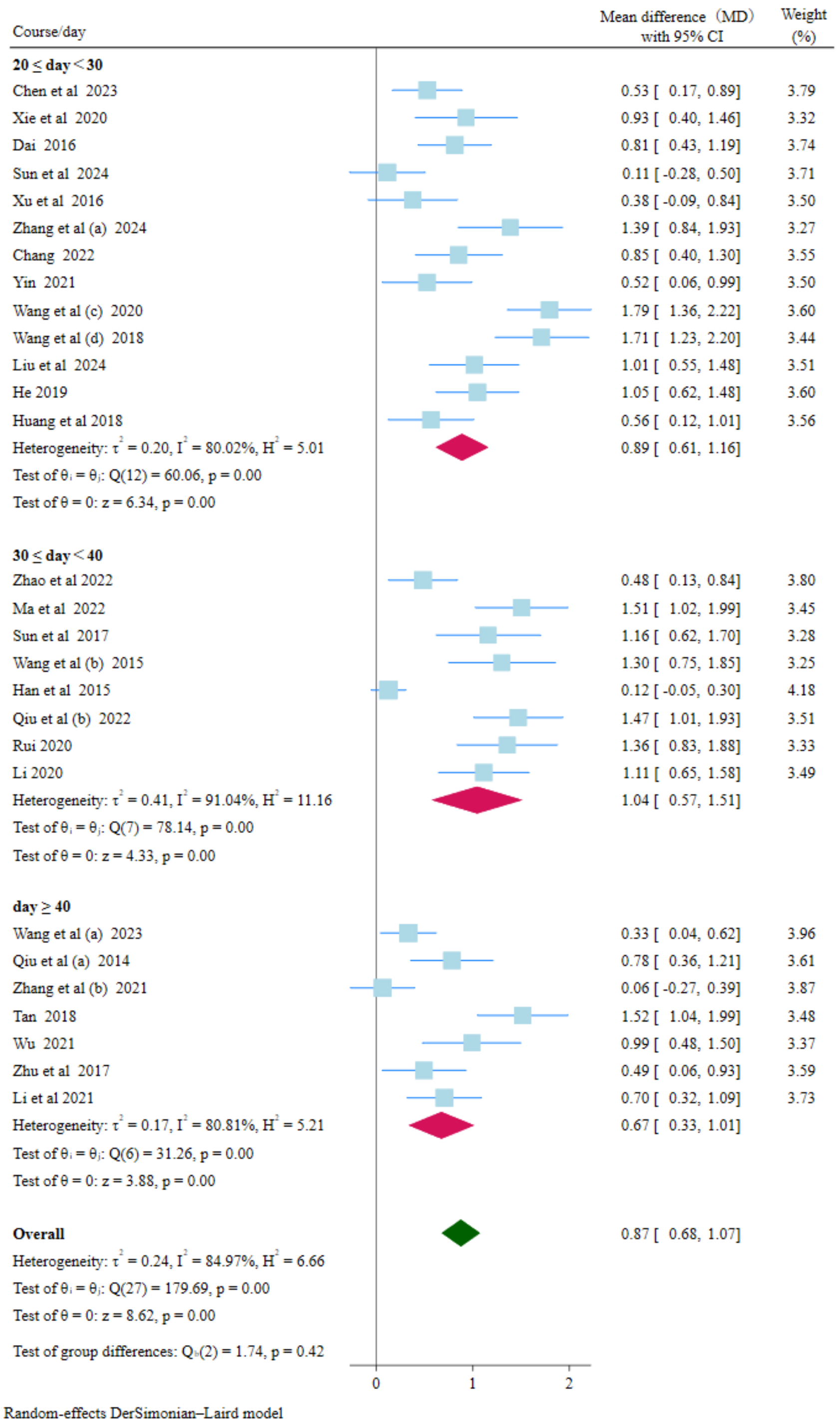

Based on 28 randomized trials. Calculated by using a random-effects model. Between-group difference: $P < 0.001$, I^2=84.97%. CI=confidence interval. Std.=standard

Figure S24 Standard mean effect sizes of FMA-L with 20≤day<30 vs. 30≤day<40 vs. 40≤day

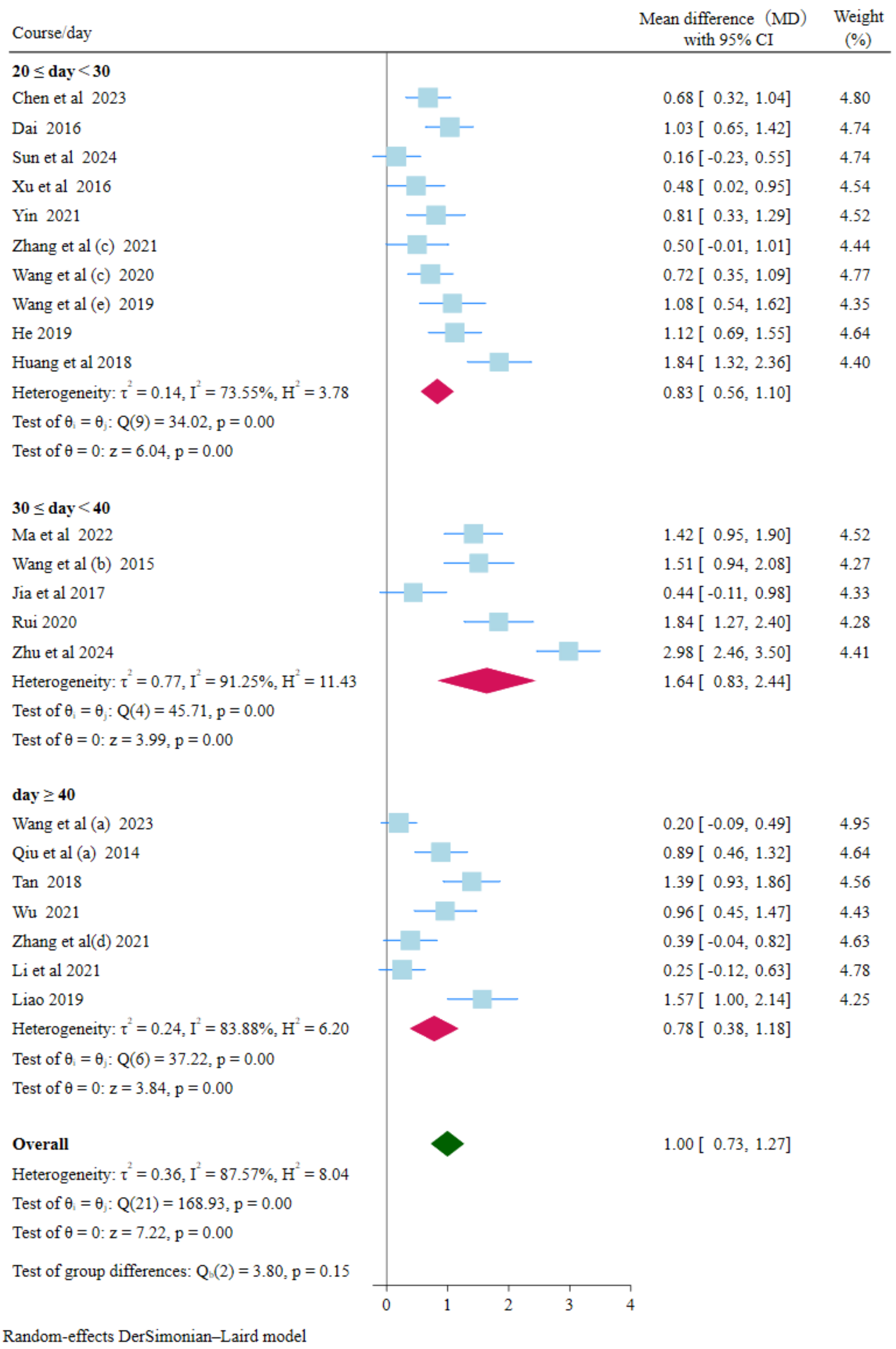

Based on 22 randomized trials. Calculated by using a random-effects model. Between-group difference: $P < 0.001$, I^2=87.57%. CI=confidence interval. Std.=standard

Figure S25 Standard mean effect sizes of MBI with 20≤day<30 vs. 30≤day<40 vs. 40≤day

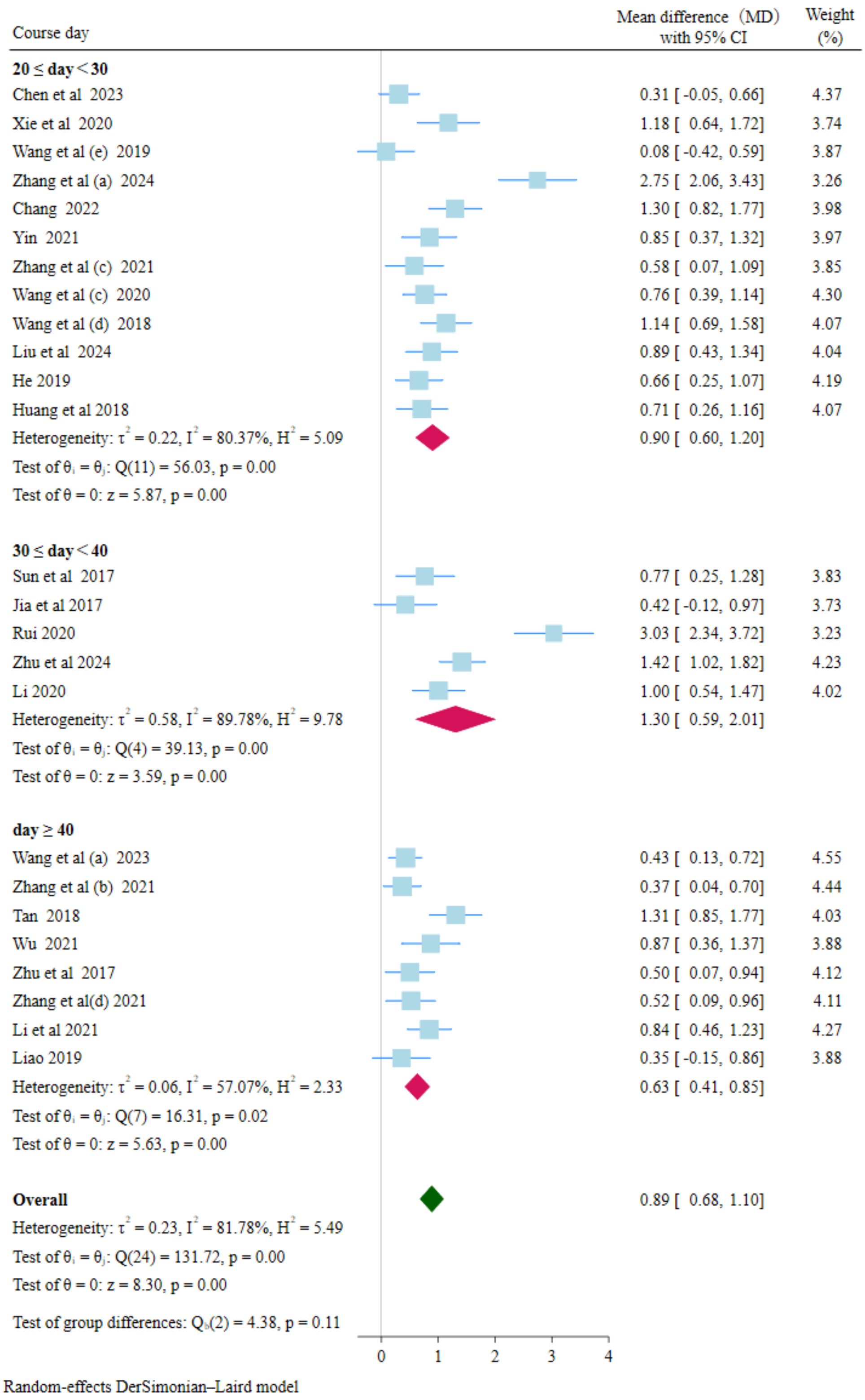

Based on 25 randomized trials. Calculated by using a random-effects model. Between-group difference: $P < 0.001$, I^2=81.78%. CI=confidence interval. Std.=standard

Figure S25 Standard mean effect sizes of Spasticity with 20≤day<30 vs. 30≤day<40 vs. 40≤day

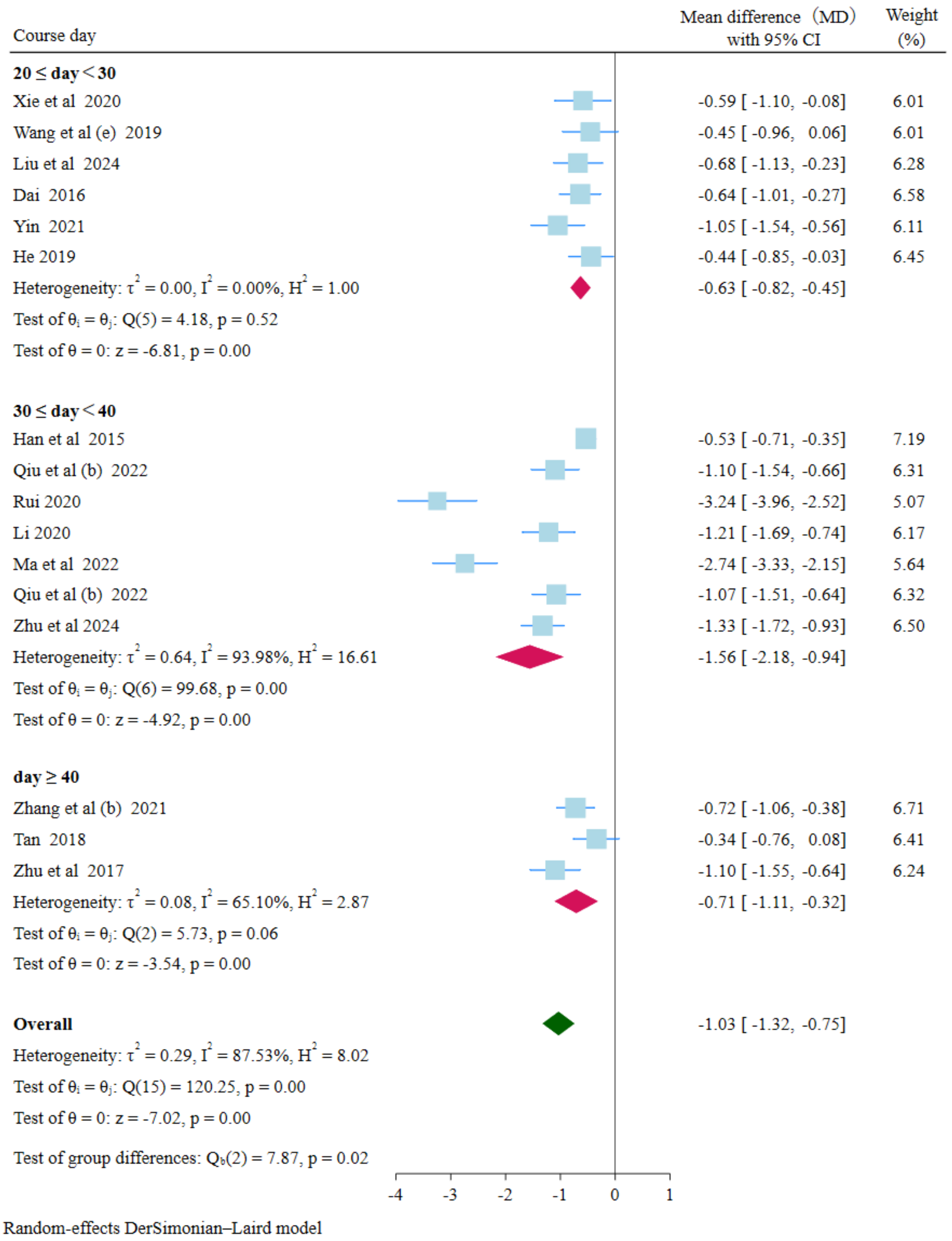

Based on 16 randomized trials. Calculated by using a random-effects model. Between-group difference: $P < 0.001$, I^2=87.53%. CI=confidence interval. Std.=standard

Supplemental file 12 Sensitivity analyses

Figure S26. Sensitivity analysis of effect on FMA-U – leave-one-out analysis

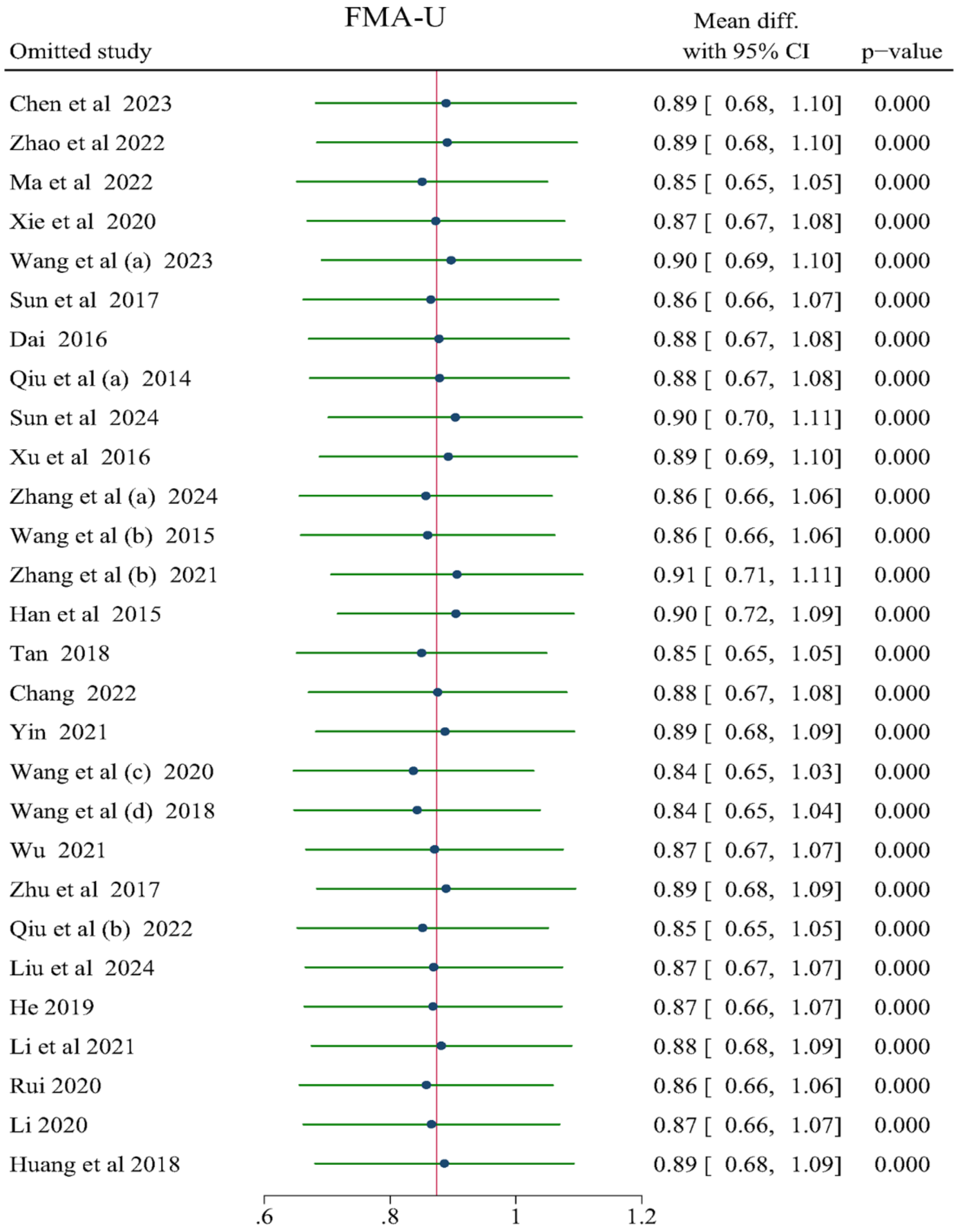

Based on 28 randomized trials. The study indicated to the left is the study excluded in the analysis. All *P*-values<0.001. CI=confidence interval; diff.=difference.

Figure S27. Sensitivity analysis of effect on FMA-L – leave-one-out analysis

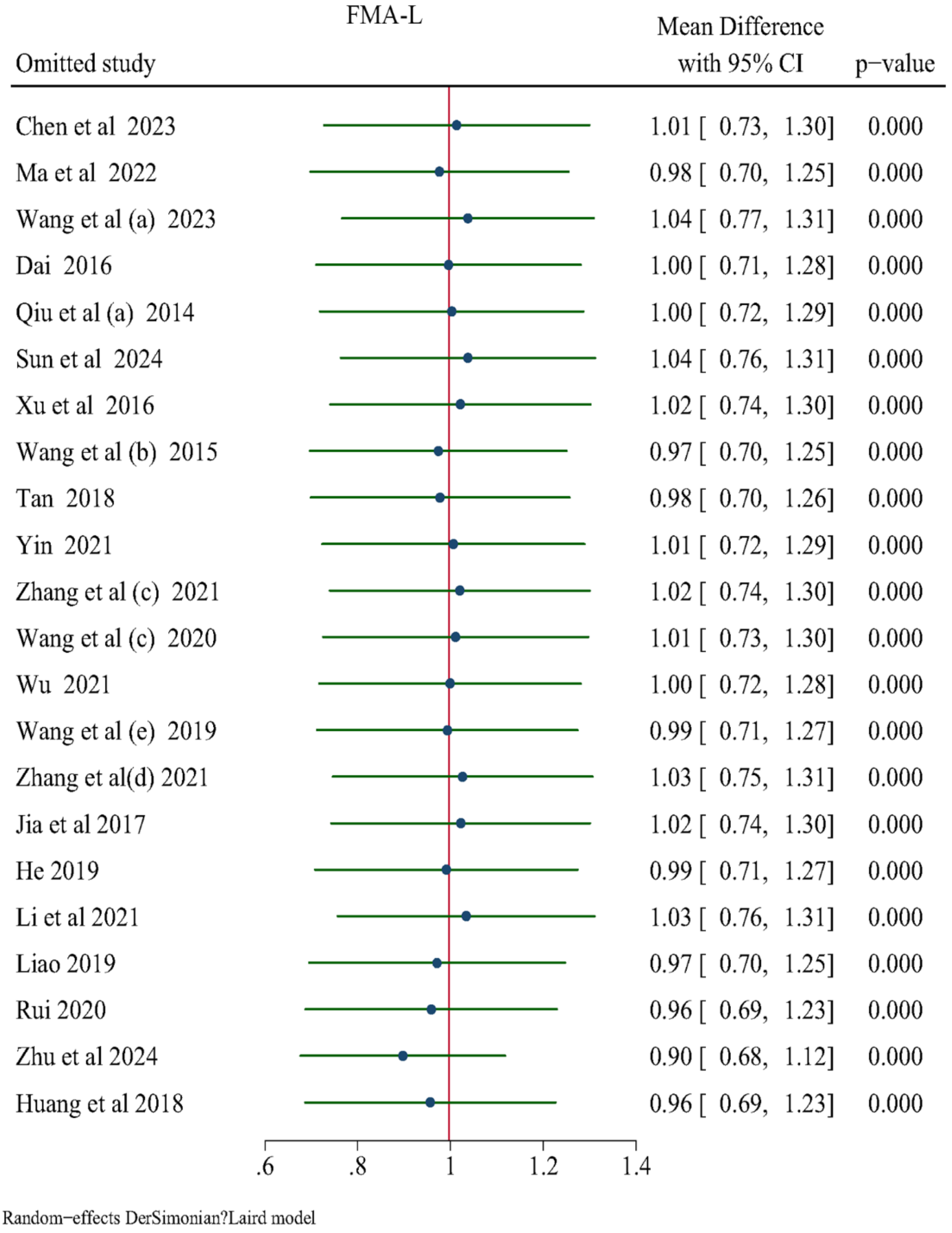

Based on 22 randomized trials. The study indicated to the left is the study excluded in the analysis. All *P*-values<0.001. CI=confidence interval.

Figure S28. Sensitivity analysis of effect on MBI – leave-one-out analysis

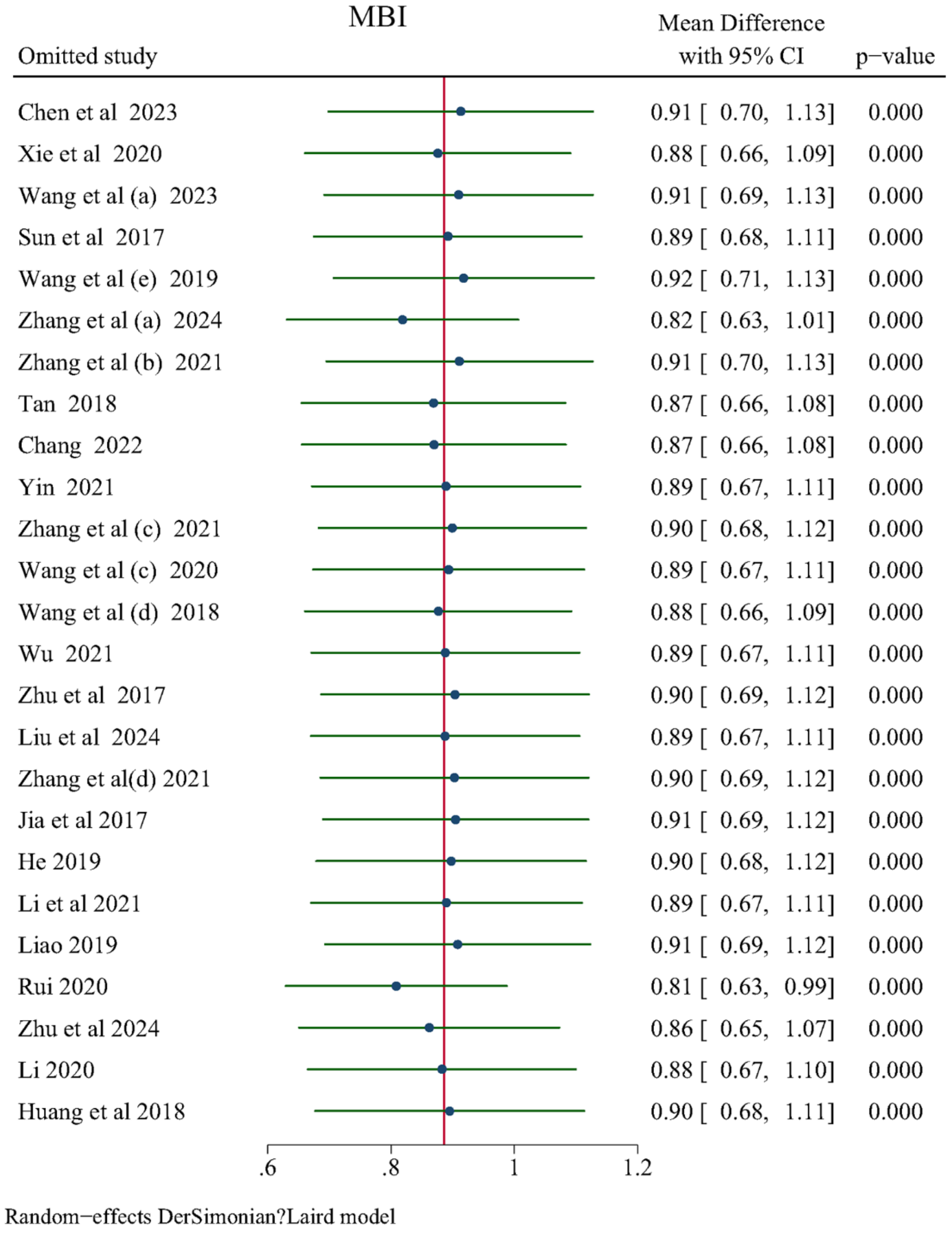

Based on 25 randomized trials. The study indicated to the left is the study excluded in the analysis. All *P*-values<0.001. CI=confidence interval.

Figure S29. Sensitivity analysis of effect on Spasticity – leave-one-out analysis

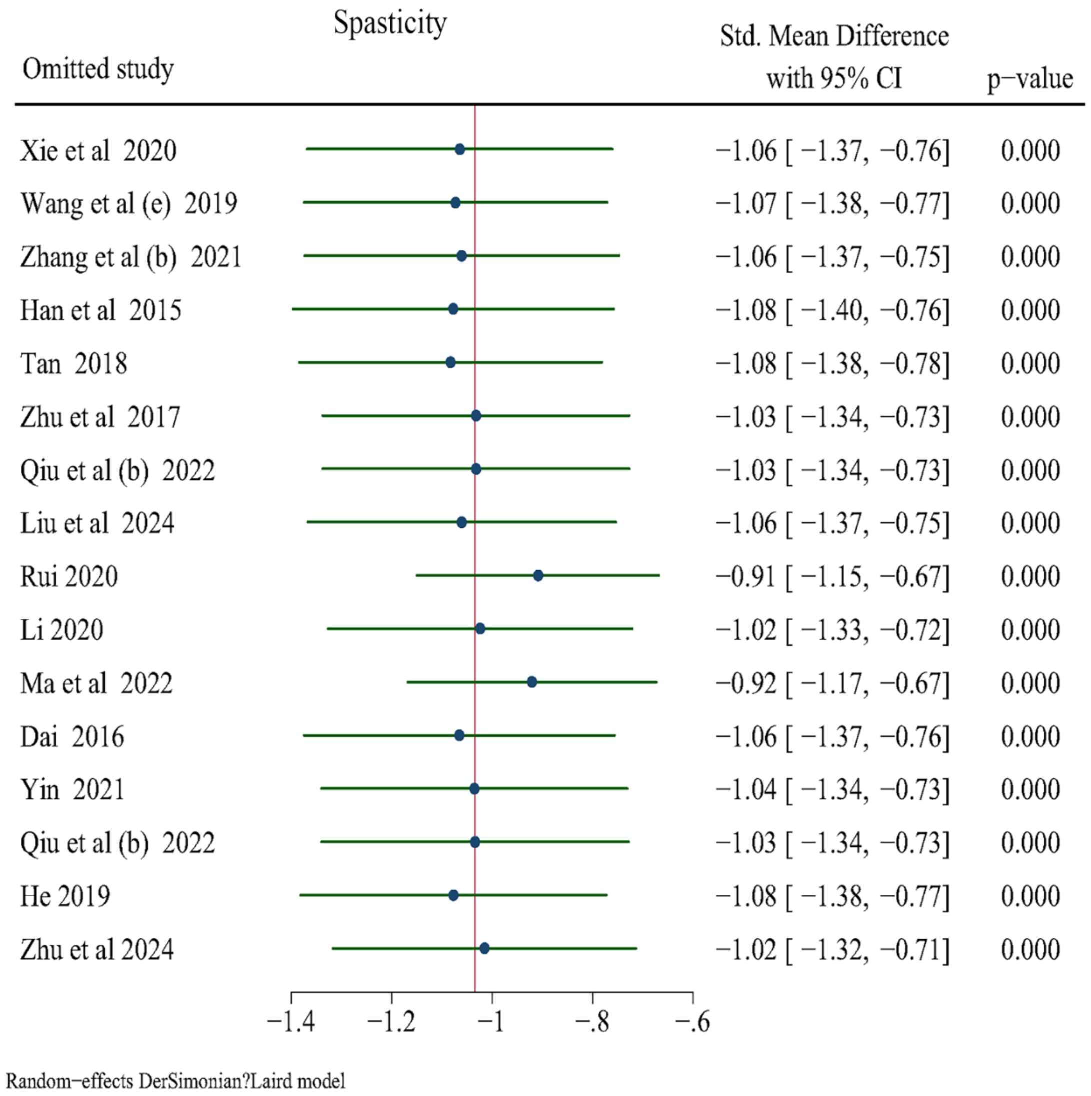

Based on 25 randomized trials. The study indicated to the left is the study excluded in the analysis. All *P*-values<0.001. CI=confidence interval.

Supplemental file 13.Funnel plots and “trim and fill” plots

Figure S30.Fuuuel plot of the effect on FMA-U

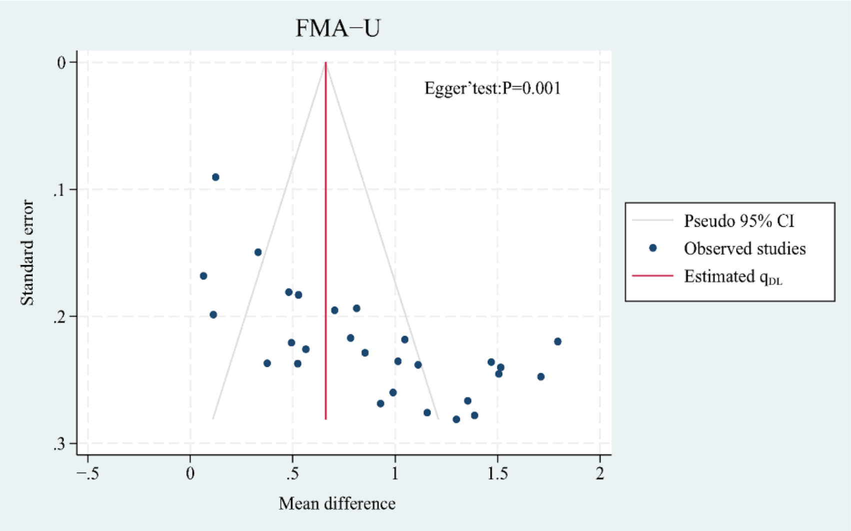

Figure S31. Trim and fill plot for the effect on FMA-U

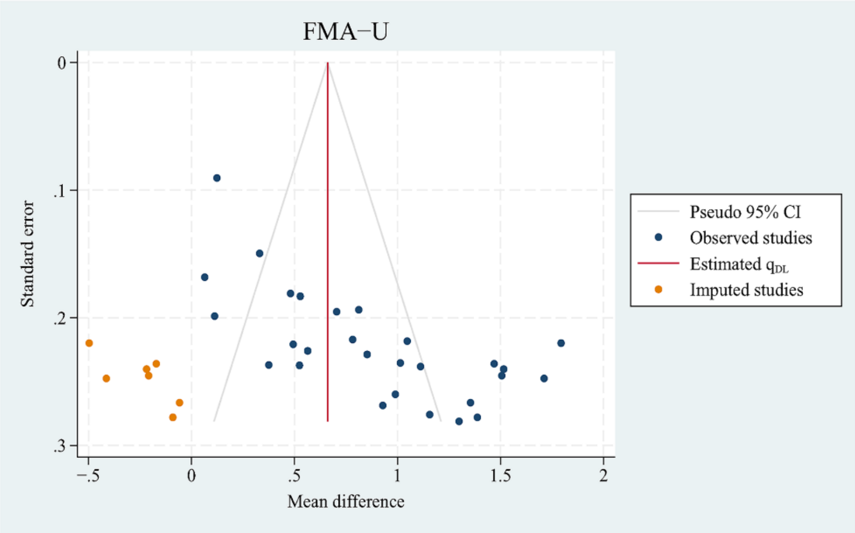

Based on 35 studies: 28 randomized trials and 7 imputed trials.

Figure S32.Fuuuel plot of the effect on FMA-L

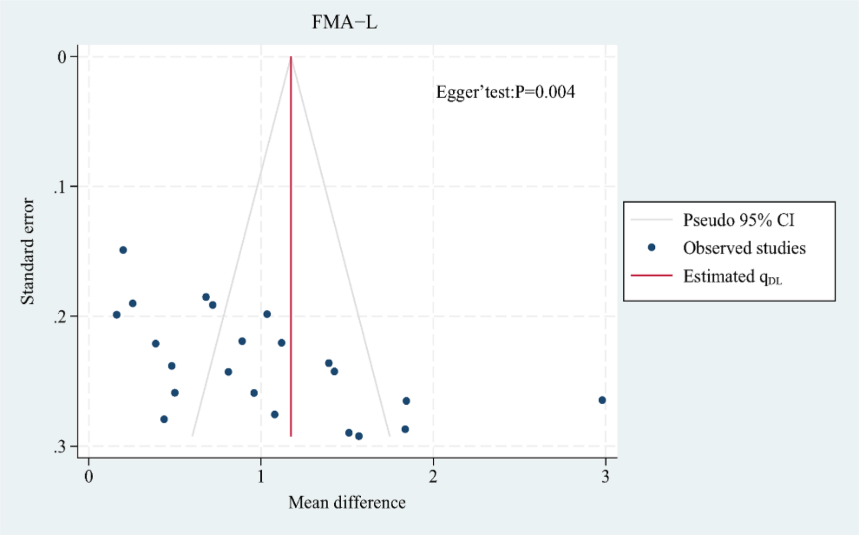

Figure S33. Trim and fill plot for the effect on FMA-L

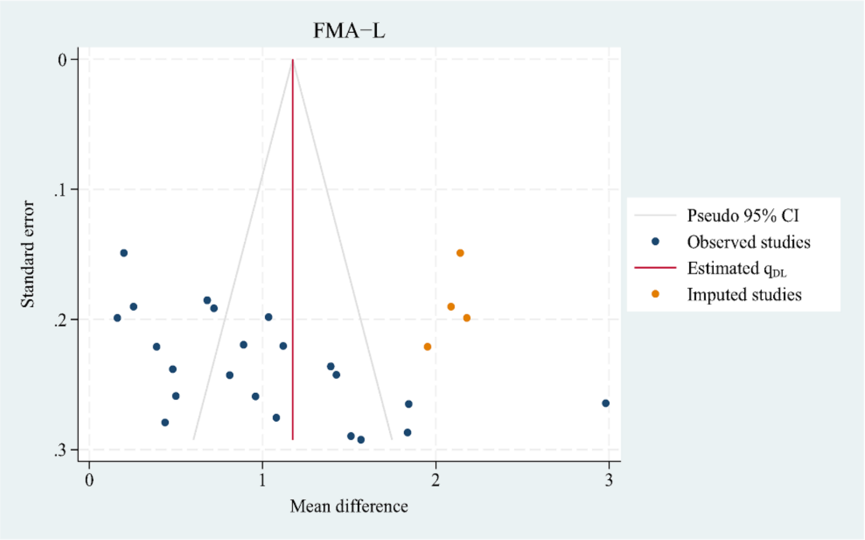

Based on 26 studies: 22 randomized trials and 4 imputed trials.

Figure S34.Fuuuel plot of the effect on MBI

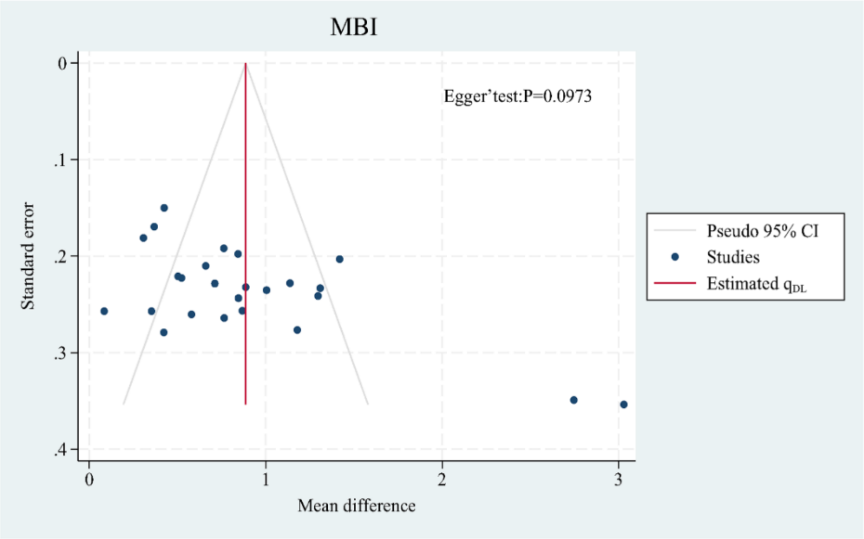

Figure S35. Trim and fill plot for the effect on MBI

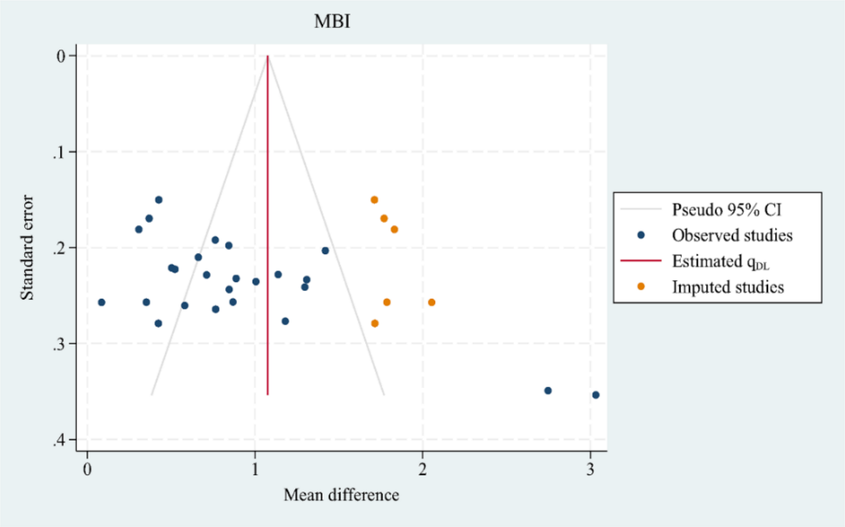

Based on 31 studies: 25 randomized trials and 6 imputed trials.

Figure S36.Fuuuel plot of the effect on Spasticity

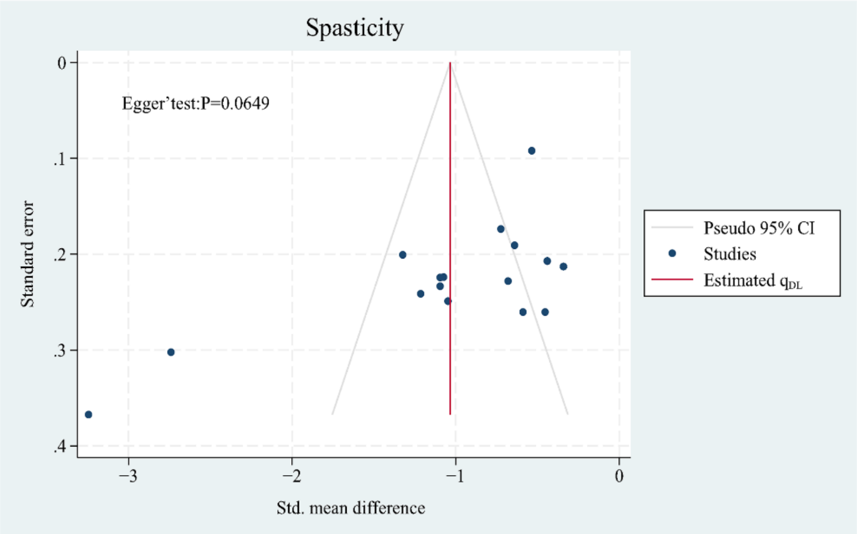

Figure S37. Trim and fill plot for the effect on Spasticity

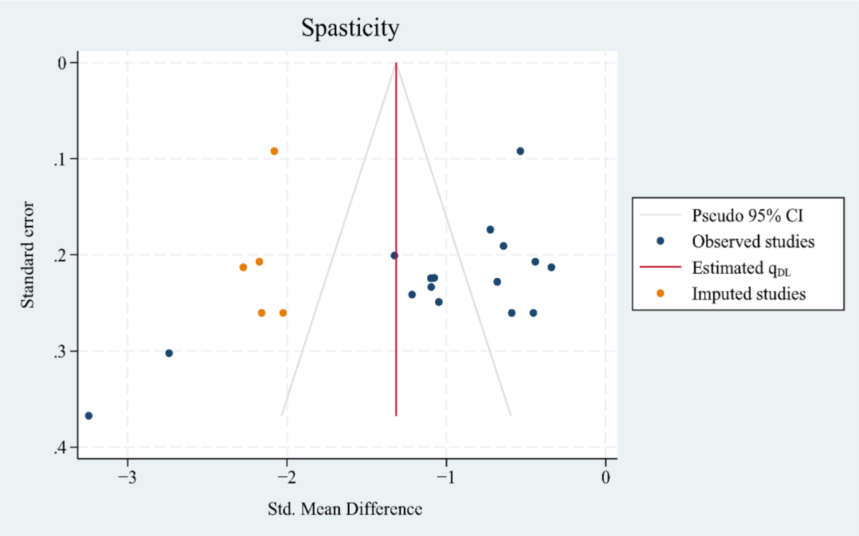

Based on 21 studies: 16 randomized trials and 5 imputed trials.

Supplemental file 14.Evaluation of Evidence Quality Based on GRADE

We used GRADE to evaluate the quality of research evidence, and when using the GRADE methodology, the quality of evidence is typically categorized into four grades: high, moderate, low, and very low.Factors affecting the quality rating of the evidence include, but are not limited to, study design, risk of bias, imprecision, non-directivity, and publication bias, and with the evaluation of each of these factors, included trials or studies may be downgraded due to the presence of the above issues , or remain unchanged or even be upgraded due to characteristics such as significant effects or dose-response gradients. Based on this principle, the quality evaluation of acupuncture types and acupuncture dose from the dimensions of FMA-U, FMA-L, MBI, and Spasticity resulted in the following summary results.

Table 3. Evaluation of the quality of research evidence based on the FMA-U

Based on FMA-U for Post-stroke spasticity

Patient or population: patients with Post-stroke spasticity

Settings:

Intervention: Acupuncture

Outcomes	Illustrative comparative risks* (95% CI)		Relative effect (95% CI)	No of Participants (studies)	Quality of the evidence (GRADE)	Comments
	Assumed risk	Corresponding risk				
	Control	FMA-U				
**Acupuncture types**		The mean acupuncture types in the intervention groups was **6.37 higher** (4.95 to 7.79 higher)		2951 (28 studies)	⊕⊕⊖⊖ **low**[1,2]	

**Acupuncture types - Electro-acupuncture**		The mean acupuncture types - electro-acupuncture in the intervention groups was **6.94 higher** (5.31 to 8.56 higher)	378 (4 studies)	⊕⊕⊕⊕ **high**[1]
**Acupuncture types - Fire acupuncture**		The mean acupuncture types - fire acupuncture in the intervention groups was **5.85 higher** (1.67 to 10.02 higher)	247 (3 studies)	⊕⊕⊖⊖ **low**[1,3]
**Acupuncture types - Manual acupuncture**		The mean acupuncture types - manual acupuncture in the intervention groups was **7.28 higher** (4.95 to 9.61 higher)	1708 (14 studies)	⊕⊖⊖⊖ **very low**[1,3,4]
**Acupuncture types - Scalp acupuncture**		The mean acupuncture types - scalp acupuncture in the intervention groups was **5.46 higher** (0.07 lower to 10.98 higher)	318 (3 studies)	⊕⊕⊕⊖ **moderate**[3,5]
**Acupuncture types - Warm acupuncture**		The mean acupuncture types - warm acupuncture in the intervention groups was **4.14 higher** (1.38 to 6.89 higher)	300 (4 studies)	⊕⊕⊖⊖ **low**[1,3,5]
**Acupuncture dose**		The mean acupuncture dose in the intervention groups was **6.37 higher** (4.95 to 7.79 higher)	2951 (28 studies)	⊕⊕⊖⊖ **low**[1,2,3]
**Acupuncture dose - High dose**		The mean acupuncture dose - high dose in the intervention groups was	1851 (20 studies)	⊕⊕⊖⊖ **low**[1,2,3]

		**5.84 higher**<br>(4.26 to 7.43 higher)			
**Acupuncture dose - Medium dose**		The mean acupuncture dose - medium dose in the intervention groups was<br>**9.72 higher**<br>(6.4 to 13.03 higher)		375<br>(5 studies)	⊕⊕⊕⊝<br>**moderate**[1]
**Acupuncture dose - Low dose**		The mean acupuncture dose - low dose in the intervention groups was<br>**4.71 higher**<br>(0.38 lower to 9.8 higher)		725<br>(3 studies)	⊕⊝⊝⊝<br>**very low**[1,2,4]

*The basis for the **assumed risk** (e.g. the median control group risk across studies) is provided in footnotes. The **corresponding risk** (and its 95% confidence interval) is based on the assumed risk in the comparison group and the **relative effect** of the intervention (and its 95% CI).

CI: Confidence interval;

GRADE Working Group grades of evidence

High quality: Further research is very unlikely to change our confidence in the estimate of effect.

Moderate quality: Further research is likely to have an important impact on our confidence in the estimate of effect and may change the estimate.

Low quality: Further research is very likely to have an important impact on our confidence in the estimate of effect and is likely to change the estimate.

Very low quality: We are very uncertain about the estimate.

[1] Studies above 2/3 of the information comes from moderate bias

[2] 95% Confidence Interval (CI) crosses the line

[3] Differences in inconsistency may be due to the magnitude of the effect, but this does not affect the conclusions

[4] Heterogeneity exceeds 75%

[5] Total sample size is less than 400

Table 4. Evaluation of the quality of research evidence based on the FMA-L

Based on FMA-L for Post-stroke spasticity

Patient or population: patients with Post-stroke spasticity

Settings:

Intervention: Acupuncture

Outcomes	Illustrative comparative risks* (95% CI)		Relative effect (95% CI)	No of Participants (studies)	Quality of the evidence (GRADE)	Comments
	Assumed risk	Corresponding risk				
	Control	FMA-L				
**Acupuncture types**		The mean acupuncture types in the intervention groups was **4.31 higher** (3.22 to 5.4 higher)		1945 (22 studies)	⊕⊕⊖⊖ **low**[1,2]	
**Acupuncture types-Electro-acupuncture**		The mean acupuncture types-electro-acupuncture in the intervention groups was **4.64 higher** (2.83 to 6.45 higher)		378 (4 studies)	⊕⊖⊖⊖ **very low**[1,2,3,4]	
**Acupuncture types - Fire acupuncture**		The mean acupuncture types - fire acupuncture in the intervention groups was **4.49 higher** (1.6 to 7.38 higher)		247 (3 studies)	⊕⊕⊖⊖ **low**[1,4]	
**Acupuncture types - Manual acupuncture**		The mean acupuncture types - manual acupuncture in the intervention groups was		732 (8 studies)	⊕⊕⊖⊖ **low**[1,5]	

		**3.63 higher**<br>(2.36 to 4.9 higher)		
**Acupuncture types - Scalp acupuncture**		The mean acupuncture types - scalp acupuncture in the intervention groups was<br>**4.41 higher**<br>(2.46 to 6.36 higher)	248<br>(3 studies)	⊕⊕⊕⊝<br>**moderate**[2,4]
**Acupuncture types - Warm acupuncture**		The mean acupuncture types - warm acupuncture in the intervention groups was<br>**4.75 higher**<br>(1.49 to 8 higher)	340<br>(4 studies)	⊕⊝⊝⊝<br>**very low**[1,3,4]
**Acupuncture dose**		The mean acupuncture dose in the intervention groups was<br>**4.31 higher**<br>(3.22 to 5.4 higher)	1945<br>(22 studies)	⊕⊕⊝⊝<br>**low**[1,2]
**Acupuncture dose - High dose**		The mean acupuncture dose - high dose in the intervention groups was<br>**4.46 higher**<br>(3.13 to 5.8 higher)	1523<br>(17 studies)	⊕⊕⊝⊝<br>**low**[1,2,3]
**Acupuncture dose - Medium dose**		The mean acupuncture dose - medium dose in the intervention groups was<br>**4.37 higher**<br>(1.47 to 7.26 higher)	185<br>(3 studies)	⊕⊕⊕⊝<br>**moderate**[2,4,6]
**Acupuncture dose - Low dose**		The mean acupuncture dose - low dose in the intervention groups was<br>**3.03 higher**<br>(1.42 to 4.63 higher)	237<br>(2 studies)	⊕⊕⊕⊝<br>**moderate**[6]

*The basis for the **assumed risk** (e.g. the median control group risk across studies) is provided in footnotes. The **corresponding risk** (and its 95% confidence interval) is based on the assumed risk in the comparison group and the **relative effect** of the intervention (and its 95% CI).

CI: Confidence interval;

GRADE Working Group grades of evidence

High quality: Further research is very unlikely to change our confidence in the estimate of effect.

Moderate quality: Further research is likely to have an important impact on our confidence in the estimate of effect and may change the estimate.

Low quality: Further research is very likely to have an important impact on our confidence in the estimate of effect and is likely to change the estimate.

Very low quality: We are very uncertain about the estimate.

[1] Studies above 2/3 of the information comes from moderate bias

[2] Differences in inconsistency may be due to the magnitude of the effect, but this does not affect the conclusions

[3] Heterogeneity exceeds 75%

[4] Total sample size is less than 400

[5] All treatment outcomes were positive, with no negative outcomes observed

[6] Heterogeneity exceeds 50% (but is less than 75%)

Table 5. Evaluation of the quality of research evidence based on the MBI

Based on MBI for Post-stroke spasticity

Patient or population: patients with Post-stroke spasticity

Settings:

Intervention: Acupuncture

Outcomes	Illustrative comparative risks* (95% CI)		Relative effect (95% CI)	No of Participants (studies)	Quality of the evidence (GRADE)	Comments
	Assumed risk	Corresponding risk				
	Control	MBI				
**Acupuncture types**		The mean acupuncture types in the intervention groups was **8.78 higher** (6.93 to 10.63 higher)		2162 (25 studies)	⊕⊕⊖⊖ **low**[1,2,3]	
**Acupuncture types - Electro-acupuncture**		The mean acupuncture types - electro-acupuncture in the intervention groups was **8.35 higher** (4.27 to 12.43 higher)		324 (4 studies)	⊕⊕⊕⊖ **moderate**[4]	
**Acupuncture types - Fire acupuncture**		The mean acupuncture types - fire acupuncture in the intervention groups was **5.52 higher** (2.68 to 8.36 higher)		187 (2 studies)	⊕⊖⊖⊖ **very low**[1,3,5]	
**Acupuncture types - Manual acupuncture**		The mean acupuncture types - manual acupuncture in the intervention groups was		780 (9 studies)	⊕⊕⊖⊖ **low**[1,2,3]	

		**10.46 higher**<br>(8.71 to 12.2 higher)			
**Acupuncture types - Scalp acupuncture**		The mean acupuncture types - scalp acupuncture in the intervention groups was<br>**8.22 higher**<br>(3.05 to 13.38 higher)		451<br>(5 studies)	⊕⊕⊕⊖<br>**moderate**[2,6]
**Acupuncture types - Warm acupuncture**		The mean acupuncture types - warm acupuncture in the intervention groups was<br>**9.07 higher**<br>(4.11 to 14.02 higher)		420<br>(5 studies)	⊕⊖⊖⊖<br>**very low**[1,5,6,7]
**Acupuncture dose**		The mean acupuncture dose in the intervention groups was<br>**8.78 higher**<br>(6.93 to 10.63 higher)		2162<br>(25 studies)	⊕⊕⊖⊖<br>**low**[1,2]
**Acupuncture dose - High dose**		The mean acupuncture dose - high dose in the intervention groups was<br>**9.34 higher**<br>(6.9 to 11.77 higher)		1520<br>(17 studies)	⊕⊕⊖⊖<br>**low**[1,2,7]
**Acupuncture dose - Medium dose**		The mean acupuncture dose - medium dose in the intervention groups was<br>**7.14 higher**<br>(5.25 to 9.04 higher)		405<br>(6 studies)	⊕⊕⊕⊖<br>**moderate**[1,3]
**Acupuncture dose - Low dose**		The mean acupuncture dose - low dose in the intervention groups was<br>**6.56 higher**<br>(1.2 to 11.92 higher)		237<br>(2 studies)	⊕⊖⊖⊖<br>**very low**[1,2,4,7]

*The basis for the **assumed risk** (e.g. the median control group risk across studies) is provided in footnotes. The **corresponding risk** (and its 95% confidence interval) is based on the assumed risk in the comparison group and the **relative effect** of the intervention (and its 95% CI).

CI: Confidence interval;

GRADE Working Group grades of evidence

High quality: Further research is very unlikely to change our confidence in the estimate of effect.

Moderate quality: Further research is likely to have an important impact on our confidence in the estimate of effect and may change the estimate.

Low quality: Further research is very likely to have an important impact on our confidence in the estimate of effect and is likely to change the estimate.

Very low quality: We are very uncertain about the estimate.

[1] Studies above 2/3 of the information comes from moderate bias

[2] Differences in inconsistency may be due to the magnitude of the effect, but this does not affect the conclusions

[3] Heterogeneity exceeds 50% (but is less than 75%)

[4] Total sample size is less than 400

[5] All treatment outcomes were positive, with no negative outcomes observed

[6] 95% Confidence Interval (CI) crosses the line

[7] Heterogeneity exceeds 75%

Table 6. Evaluation of the quality of research evidence based on the Spasticity

Based on Spasticity for Post-stroke spasticity

Patient or population: patients with Post-stroke spasticity

Settings:

Intervention: Acupuncture

Outcomes	Illustrative comparative risks* (95% CI)		Relative effect (95% CI)	No of Participants (studies)	Quality of the evidence (GRADE)	Comments
	Assumed risk	Corresponding risk				
	Control	Spasticity				
**Acupuncture types**		The mean acupuncture types in the intervention groups was **1.03 standard deviations lower** (1.32 to 0.74 lower)		1810 (15 studies)	⊕⊕⊖⊖ **low**[1,2]	SMD -1.03 (-1.32 to -0.74)
**Acupuncture types - Electro-acupuncture**		The mean acupuncture types - electro-acupuncture in the intervention groups was **0.56 standard deviations lower** (0.8 to 0.31 lower)		268 (3 studies)	⊕⊕⊕⊖ **moderate**[3]	SMD -0.56 (-0.8 to -0.31)
**Acupuncture types - Manual acupuncture**		The mean acupuncture types - manual acupuncture in the intervention groups was **0.97 standard deviations lower** (1.38 to 0.56 lower)		1062 (7 studies)	⊕⊕⊖⊖ **low**[1,3]	SMD -0.97 (-1.38 to -0.56)
**Acupuncture types - Scalp acupuncture**		The mean acupuncture types - scalp acupuncture in the intervention groups was		140 (1 study)	⊕⊕⊖⊖ **low**[3,4]	SMD -0.72 (-1.07 to -0.38)

		**0.72 standard deviations lower**<br>(1.07 to 0.38 lower)				
**Acupuncture types - Warm Acupuncture**		The mean acupuncture types - warm acupuncture in the intervention groups was<br>**1.66 standard deviations lower**<br>(2.41 to 0.9 lower)		340<br>(4 studies)	⊕⊖⊖⊖<br>**very low**[1,3,4]	SMD -1.66 (-2.41 to -0.9)
**Acupuncture dose**		The mean acupuncture dose in the intervention groups was<br>**1.03 standard deviations lower**<br>(1.32 to 0.74 lower)		1810<br>(15 studies)	⊕⊕⊖⊖<br>**low**[1,2]	SMD -1.03 (-1.32 to -0.74)
**Acupuncture dose - High dose**		The mean acupuncture dose - high dose in the intervention groups was<br>**1.11 standard deviations lower**<br>(1.54 to 0.68 lower)		982<br>(11 studies)	⊕⊖⊖⊖<br>**very low**[1,2,3]	SMD -1.11 (-1.54 to -0.68)
**Acupuncture dose - Medium dose**		The mean acupuncture dose - medium dose in the intervention groups was<br>**1.01 standard deviations lower**<br>(1.24 to 0.79 lower)		340<br>(3 studies)	⊕⊕⊕⊖<br>**moderate**[4]	SMD -1.01 (-1.24 to -0.79)
**Acupuncture dose - Low dose**		The mean acupuncture dose - low dose in the intervention groups was<br>**0.53 standard deviations lower**<br>(0.72 to 0.35 lower)		488<br>(1 study)	⊕⊖⊖⊖<br>**very low**[2,3,4]	SMD -0.53 (-0.72 to -0.35)
**Outcomes**		The mean outcomes in the intervention groups was<br>**1.03 standard deviations lower**<br>(1.32 to 0.74 lower)		1810<br>(15 studies)	⊕⊖⊖⊖<br>**very low**[1,2,3]	SMD -1.03 (-1.32 to -0.74)

**Outcomes - MAS**		The mean outcomes - mas in the intervention groups was **0.94 standard deviations lower** (1.28 to 0.6 lower)	1236 (10 studies)	⊕⊝⊝⊝ **very low**[1,2,3]	SMD -0.94 (-1.28 to -0.6)
**Outcomes - CSI**		The mean outcomes - csi in the intervention groups was **1.19 standard deviations lower** (1.73 to 0.64 lower)	574 (6 studies)	⊕⊝⊝⊝ **very low**[1,2,3]	SMD -1.19 (-1.73 to -0.64)

*The basis for the **assumed risk** (e.g. the median control group risk across studies) is provided in footnotes. The **corresponding risk** (and its 95% confidence interval) is based on the assumed risk in the comparison group and the **relative effect** of the intervention (and its 95% CI).

CI: Confidence interval;

GRADE Working Group grades of evidence

High quality: Further research is very unlikely to change our confidence in the estimate of effect.

Moderate quality: Further research is likely to have an important impact on our confidence in the estimate of effect and may change the estimate.

Low quality: Further research is very likely to have an important impact on our confidence in the estimate of effect and is likely to change the estimate.

Very low quality: We are very uncertain about the estimate.

[1] Studies above 2/3 of the information comes from moderate bias

[2] Asymmetric funnel plot

[3] Differences in inconsistency may be due to the magnitude of the effect, but this does not affect the conclusions

[4] Total sample size is less than 400

Supplemental file 15. The cumulative ranking curve rank by SCURA

Table 7. The SCURA based on different acupuncture types

Acupuncture types	SUCRA(%)
Warm acupuncture	94.6
Manual acupuncture	62.6
Scalp acupuncture	47.7
Electro-acupuncture	38.9
Conventional therapy	6.1

Figure S38. Surface under the cumulative ranking curve rank for acupuncture types

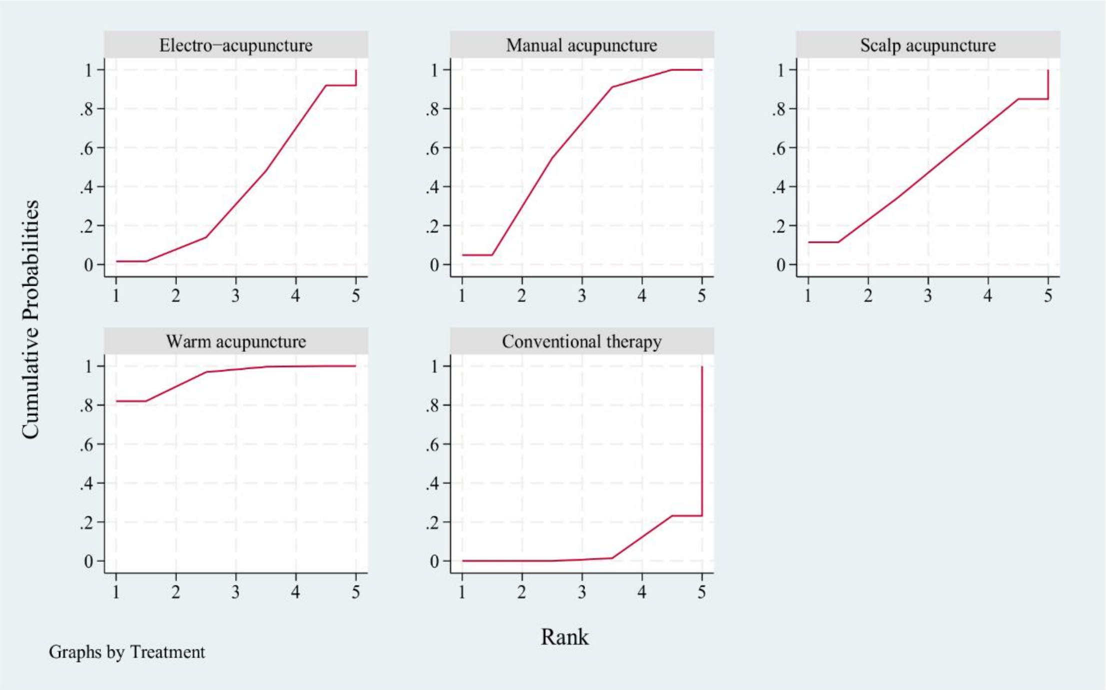

Table 7. The SCURA based on different acupuncture dose

Acupuncture types	SUCRA(%)
High dose	78.5
Medium dose	70.9
Low dose	42.1
Conventional therapy	8.5

Figure S39. Surface under the cumulative ranking curve rank for acupuncture dose

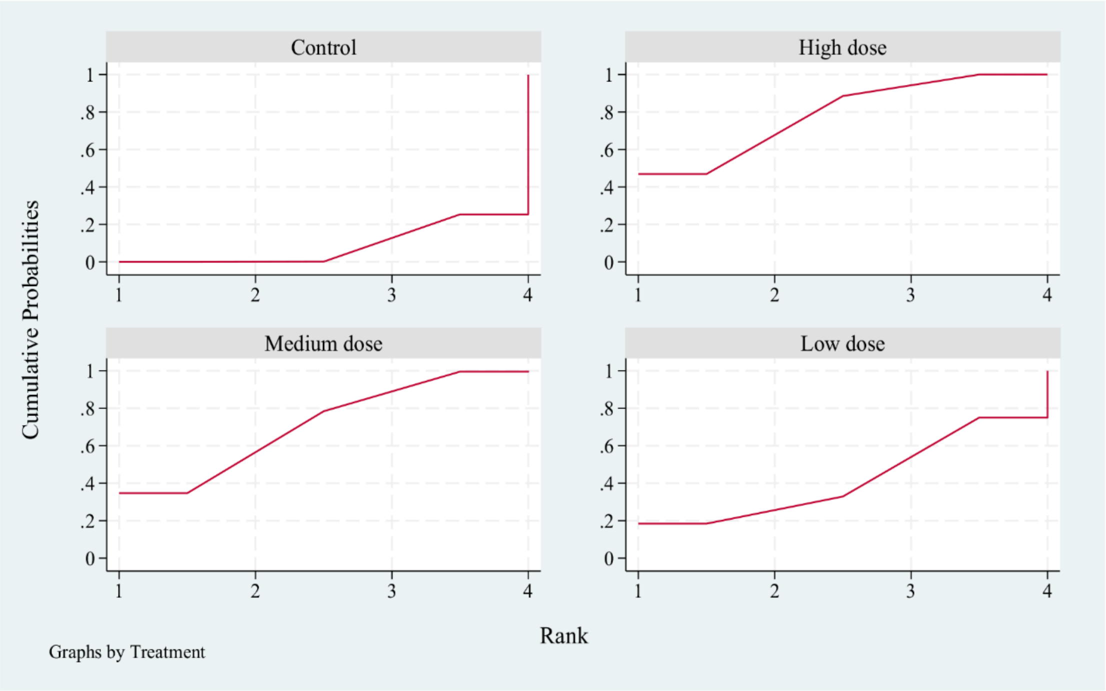

Supplemental file 16. The League tables for the effect of acupuncture on spasticity with PSS patients

(A) Acupuncture types

**Warm acupuncture**	0.67 (-0.21,1.55)	0.93 (-0.64,2.49)	1.10 (0.00,2.19)	1.65 (0.93,2.38)
-0.67 (-1.55,0.21)	**Manual acupuncture**	0.26 (-1.22,1.73)	0.42 (-0.53,1.38)	0.98 (0.48,1.48)
-0.93 (-2.49,0.64)	-0.26 (-1.73,1.22)	**Scalp acupuncture**	0.17 (-1.44,1.78)	0.72 (-0.66,2.11)
**-1.10 (-2.19,-0.00)**	-0.42 (-1.38,0.53)	-0.17 (-1.78,1.44)	**Electro-acupuncture**	0.56 (-0.26,1.37)
**-1.65 (-2.38,-0.93)**	**-0.98 (-1.48,-0.48)**	-0.72 (-2.11,0.66)	-0.56 (-1.37,0.26)	**Conventional therapy**

(B)Acupuncture dose

**High dose**	0.10 (-0.82,1.02)	0.58 (-1.00,2.16)	1.12 (0.64,1.59)
-0.10 (-1.02,0.82)	**Medium dose**	0.48 (-1.22,2.18)	1.02 (0.23,1.80)
-0.58 (-2.16,1.00)	-0.48 (-2.18,1.22)	**Low dose**	0.53 (-0.98,2.04)
**-1.12 (-1.59,-0.64)**	**-1.02 (-1.80,-0.23)**	-0.53 (-2.04,0.98)	**Conventional threapy**

Supplemental file 17. Sensitivity analysis of effect on high-dose

Figure S40. Sensitivity analysis of effect on high-dose

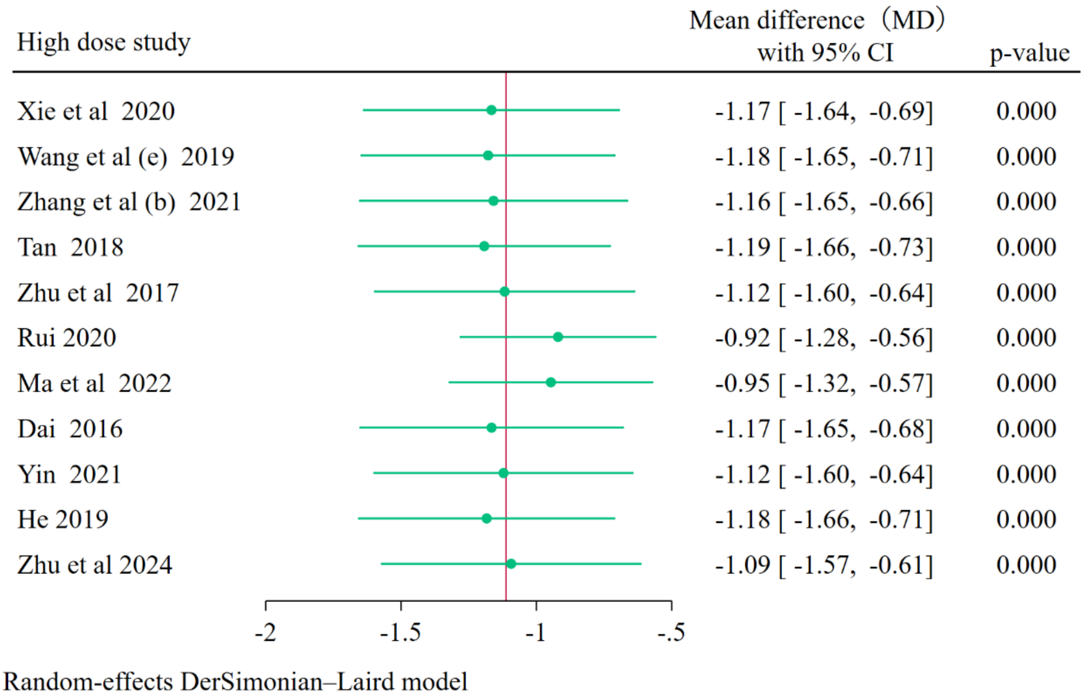

Figure S41. Sensitivity analysis of effect on frequency≥2week

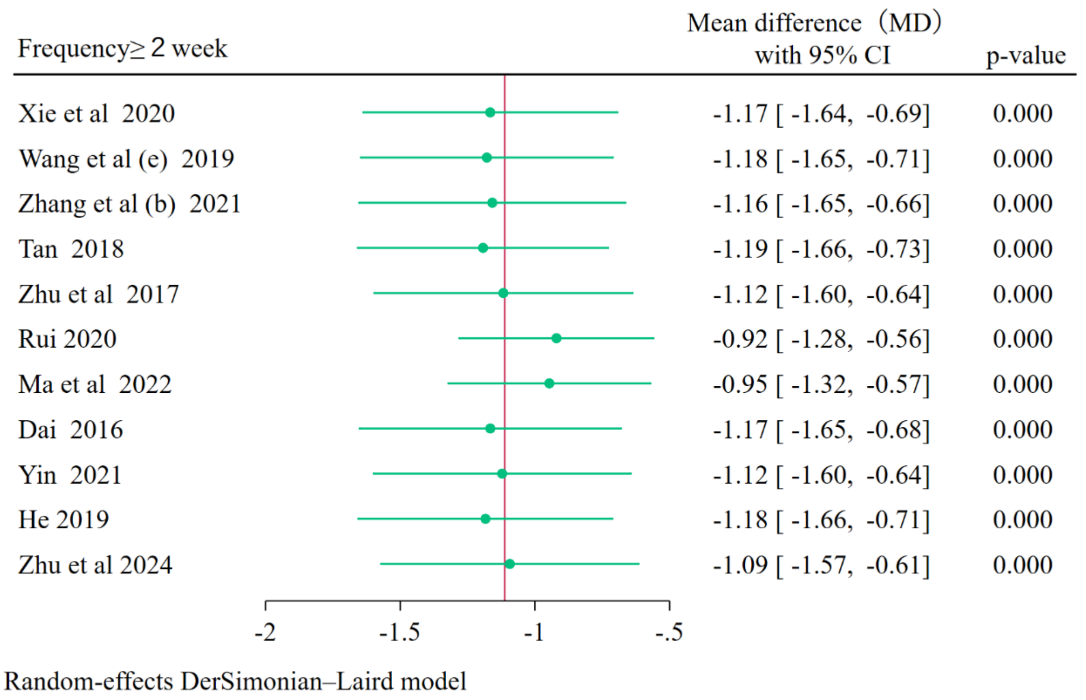

Figure S42. Sensitivity analysis of effect on sessions≥8times

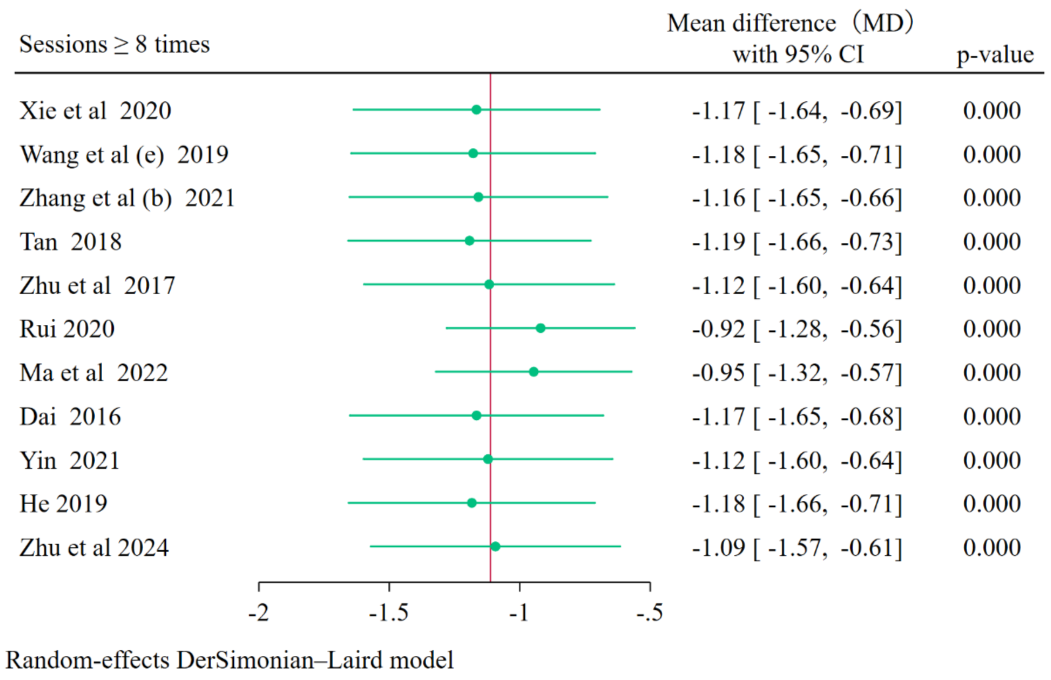

Figure S43. Sensitivity analysis of effect on acupionts≥9

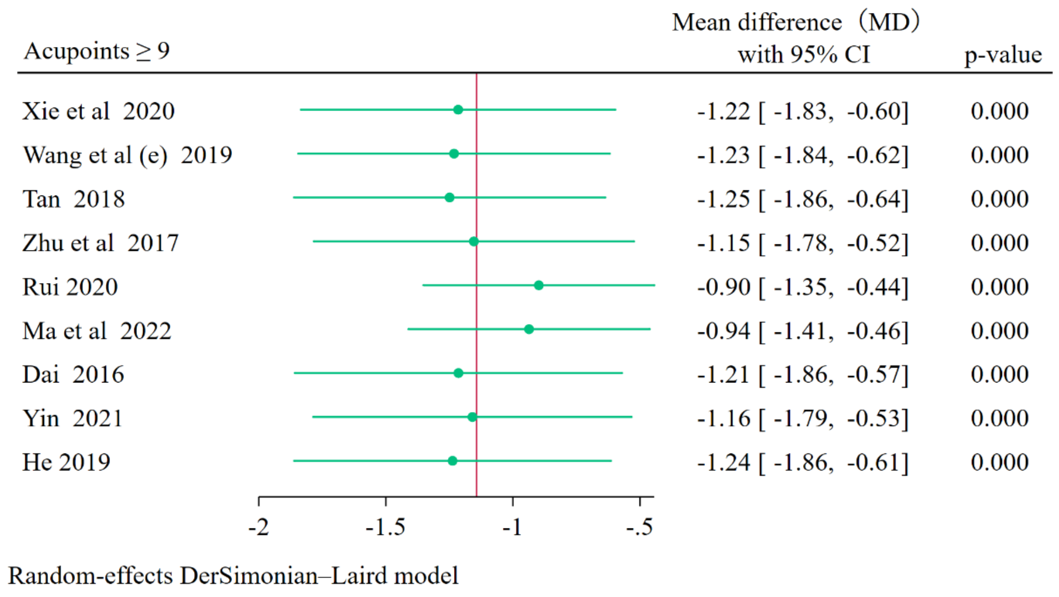

Figure S44. Sensitivity analysis of effect on Deqi

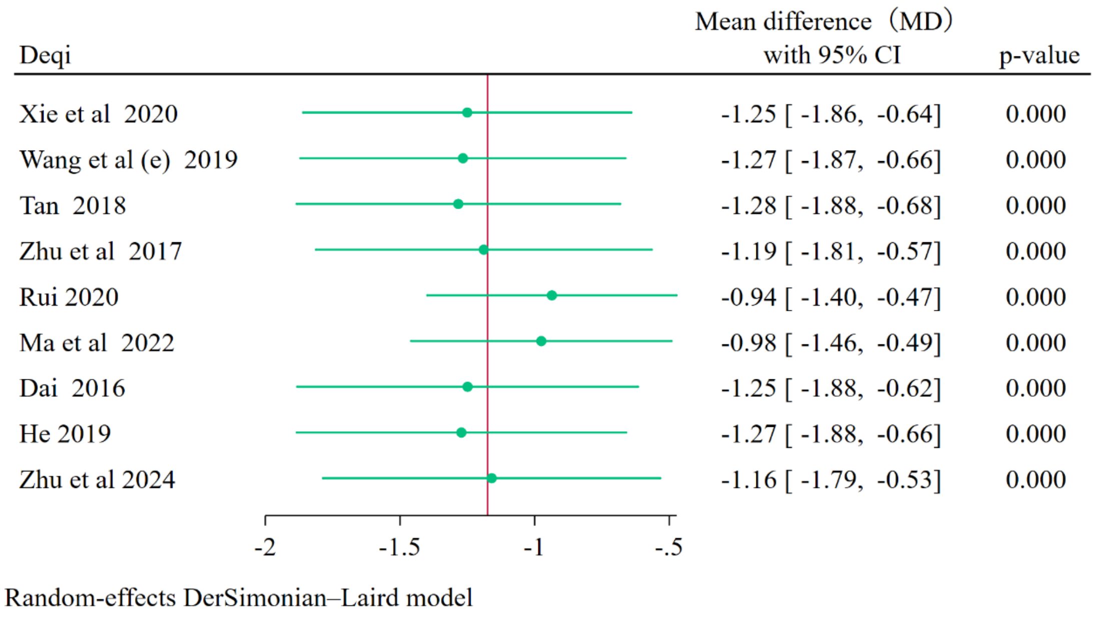

Based on Figure S40 to 44, leave-one-out sensitivity analyses were conducted for each component of high-dose acupuncture. The results demonstrated that the findings for each component were robust.
